# Samae Dam chicken: a variety of the Pradu Hang Dam breed revealed from microsatellite genotyping data

**DOI:** 10.5713/ab.24.0161

**Published:** 2024-06-25

**Authors:** Nivit Tanglertpaibul, Trifan Budi, Chien Phuoc Tran Nguyen, Worapong Singchat, Wongsathit Wongloet, Nichakorn Kumnan, Piangjai Chalermwong, Anh Huynh Luu, Kantika Noito, Thitipong Panthum, Pish Wattanadilokchatkun, Anuphong Payopat, Natthamon Klinpetch, Aingorn Chaiyes, Kanithaporn Vangnai, Chotika Yokthongwattana, Chomdao Sinthuvanich, Syed Farhan Ahmad, Narongrit Muangmai, Kyudong Han, Mitsuo Nunome, Akihiko Koga, Prateep Duengkae, Sompon Waipanya, Yoichi Matsuda, Kornsorn Srikulnath

**Affiliations:** 1 Animal Genomics and Bioresource Research Unit (AGB Research Unit), Faculty of Science, Kasetsart University, Bangkok 10900, Thailand; 2 Interdisciplinary Graduate Program in Bioscience, Faculty of Science, Kasetsart University, Bangkok 10900, Thailand; 3 Special Research Unit for Wildlife Genomics (SRUWG), Department of Forest Biology, Faculty of Forestry, Kasetsart University, Bangkok 10900, Thailand; 4 School of Agriculture and Cooperatives, Sukhothai Thammathirat Open University, Nonthaburi 11120, Thailand; 5 Department of Food Science and Technology, Faculty of Agro-Industry, Kasetsart University, Bangkok, 10900, Thailand; 6 Department of Biochemistry, Faculty of Science, Kasetsart University, Bangkok 10900, Thailand; 7 Department of Fishery Biology, Faculty of Fisheries, Kasetsart University, Bangkok 10900, Thailand; 8 Department of Microbiology, Dankook University, Cheonan 31116, Korea; 9 Center for Bio-Medical Engineering Core-Facility, Dankook University, Cheonan 31116, Korea; 10 Smart Animal Bio Institute, Dankook University, Cheonan 31116, Korea; 11 Department of Zoology, Faculty of Science, Okayama University of Science, Kita-ku, Okayama 700-0005, Japan; 12 Department of Livestock Development, Ministry of Agriculture and Cooperative, Bangkok 10900, Thailand; 13 Laboratory of Animal Cytogenetics and Comparative Genomics (ACCG), Department of Genetics, Faculty of Science, Kasetsart University, Bangkok 10900, Thailand

**Keywords:** Breed, Domestication, Genetic Structure, Indigenous Chicken, Microsatellite, Variety

## Abstract

**Objective:**

The remarkable adaptability to the environment, high growth rate, meat with good taste and aroma, and ornamental appearance of the Pradu Hang Dam (PDH) and Samae Dam (SD) chickens make them valuable for improvement of poultry production to enhance food security. However, despite their close phenotypic similarity, distinct classification of PDH and SD chickens remains controversial. Thus, this study aimed to clarify genetic origins and variation between PDH and SD chickens, genetic diversity and structures of PDH and SD chickens.

**Methods:**

This study analyzed 5 populations of PDH and 2 populations of SD chickens using 28 microsatellite markers and compared with those of other indigenous and local chicken breeds using Thailand’s “The Siam Chicken Bioresource Project” database.

**Results:**

Considerably high genetic variability was observed within PDH (370 total alleles; 4.086±0.312 alleles/locus) and SD chickens (179 total alleles; 3.607±0.349 alleles/locus). A partial overlap of gene pools was observed between SD chickens from the Department of Livestock, Uthai Thani (SD1) and PDH chickens, suggesting a potentially close relationship between the two chicken breeds. A gene pool that partially overlapped with that of the red junglefowl was observed in the SD chicken population from the Sanhawat Farm Uthai Thani population (SD2). Distinct subclusters were observed within SD chickens, indicating the possibility that genetic differentiation occurred early in the process of establishment of SD chickens.

**Conclusion:**

These findings could offer valuable insights into genetic verification of Thai local chicken breeds and their sustainable conservation and utilization.

## INTRODUCTION

Diverse genetic resources support the sustainable development of livestock production. These resources are essential for providing resilience and adaptability to challenges, such as climate change, disease outbreaks, variation of feed and water demands, and changes in market needs. However, these resources are often potentially endangered by poor management and threats. Local livestock breeds are often lost due to economic, ecological or social changes, government’s pressure, and misconceptions about short-term profit versus long-term profit [1]. Urgent action is therefore recommended to enhance the sustainable management of these resources. Indigenous and local chicken breeds in Thailand exhibit adaptations to various agro-climatic conditions in the tropical area, which provides value to them as genetic resources. Their flexible adaptability to the environments allows them to improve thermal resistance, disease tolerance, and mortality in the tropical region [2,3].

The Pradu Hang Dam (PDH) chicken, an indigenous breed in Thailand, has been attracting significant attention from farmers owing to its distinctive traits that meet market demands [4]. It is characterized by distinctive black tail feathers, body feathers mixed with red, a red and black face, and a pea comb. Male body weight is 2.5 to 2.8 kg while female body weight is 1.8 to 2.0 kg [5]. Each hen produces approximately 95 chicks in 100 days and an average of 175 eggs in one year [5]. Its popularity in farming is primarily due to its good growth performance and tasty meat, and its attractive external morphology is also popular with chicken lovers in this country. Based on large-scale population genetics studies of indigenous and local chicken breeds and red junglefowl in Thailand using microsatellite markers, PDH chickens are classified into type II [6], with a variety of desired traits maintained by fanciers. The genetic footprint of PDH chickens is thought to be the result of rapid human-led selection in small populations [6].

Samae Dam (SD) chickens, which are closely related to PDH chickens, are bred in the Uthai Thani province, Central Thailand [7]. They are believed to have been favored by King Ramkhamhaeng, the Great from the Kingdom of Sukhothai (an ancient city in Thailand) [7]. The distinctive characteristics of this breed is an appearance covered entirely in black including bones and skin, such as black beak, shanks, spurs, eyes, earlobes, and wing and tail feathers except for burnt tamarind-colored hackle and collars [7]. Males and females of SD chickens weigh 3.0 to 3.5 kg and 2.0 to 3.0 kg, respectively. They also have an entirely black appearance, with females’ face and skin being darker than males. The classification of PDH and SD chickens remains controversial (Sompon Waipanya, personal communication). Some communities suggest to include SD chickens in PDH chickens as a subgroup, whereas the local community of Uthai Thani advocates to distinguish SD chickens as a distinct breed from their meat quality and ornamental appearance.

The conservation and promotion of SD chickens as an established breed could boost the growth of the economy in the Uthai Thani community. Currently, only two chicken farms preserve SD chickens, and they are at risk of near extinction in this country. Despite their significance to the local economy, the origins and breeding processes of SD chickens remain unclear. The value of SD chickens as a genetic resource can be assessed by comparing their genetic diversity and structure with those of red junglefowl and other indigenous and local chicken breeds in Thailand, such as PDH chickens. In the present study, in order to investigate the genetic origin and genetic structure of SD chickens and genetic difference between PDH and SD chickens, the genetic diversity of SD chickens was examined using 28 microsatellite markers.

To prevent sampling bias and errors, the samples of PDH chickens were collected from multiple populations across Thailand, and the following hypotheses were tested: i) distinct gene pools of PDH and SD chickens, which proves that they are differentiated breeds, are revealed through genetic testing, and ii) a high level of inbreeding is observed in the small number of chicken farms maintaining SD chickens. The results were compared with the data of an extensive gene pool library obtained from “The Siam Chicken Bioresource Project, SCBP” (https://www.sci.ku.ac.th/scbp/ and Dryad dataset: https://datadryad.org/stash/share/x2qlPmboMgCROXO8, accessed on January 15, 2024) to examine the genetic structures of PDH and SD chickens [6,8–13]. With the explicit goal of preserving genetic diversity in genetic stocks, these chicken breeds may be considered to serve as vital repositories of invaluable genetic information.

## MATERIALS AND METHODS

### Ethical statement

The experimental protocol for this study was approved by the Animal Experiment Committee of Kasetsart University (approval numbers: ACKU65-SCI-021 and ACKU65-SCI-023) and was conducted in accordance with the Regulations on Animal Experiments at Kasetsart University and the ARRIVE guidelines (https://arriveguidelines.org).

### Specimen collection and DNA extraction

Whole blood samples were collected from five populations of PDH chickens and two populations of SD chickens. The data of one population of PDH chickens (Phitsanulok 1: PDH1) was obtained from our previous study [8] (Supplementary Table S1). The PDH2 population was derived from Phitsanulok 2, PDH3 was from Chiang Mai, PDH4 was from Nakhon Pathom, and PDH5 was from Nonthaburi. SD1 population of SD chickens was derived from the Department of Livestock, Uthai Thani population, and SD2 was derived from Sanhawat Farm, Uthai Thani. Specimen collection, DNA extraction, and DNA qualification and quantification were performed as described previously [10].

### Microsatellite genotyping and data analysis

Twenty-eight microsatellite primer sets were selected based on the recommendations of the Food and Agriculture Organization for assessing the biodiversity of chicken populations (Supplementary Table S2). The 5′-end of the forward primer of each primer set was labeled with fluorescein amidite (6-FAM) or hexachloro-fluorescein (HEX) (Macrogen Inc., Seoul, Korea). Microsatellite PCR amplification was performed using a 15 μL reaction mixture containing approximately 1× Apsalagen PCR buffer (Apsalagen Co., Ltd., Bangkok, Thailand), 25 ng genomic DNA, 1.5 mM MgCl_2_, 0.2 mM dNTPs, 0.5 μM of each primer, and 0.5 U *Taq* polymerase (Apsalagen, Thailand). The PCR conditions were as follows: initial denaturation at 95°C for 10 min, followed by 35 cycles at 95°C for 30 s, 55°C for 30 s, and 72°C for 30 s, and a final extension for 5 min at 72°C, as described previously [10]. To avoid false allele amplification, each individual sample was run in triplicate.

Genetic diversity parameters, such as allelic richness (AR) were calculated in FSTAT version 2.9.4 [14], polymorphic information content (PIC) was calculated in Excell Microsatellite Toolkit [15], number of alleles per population (*N*_a_), observed and expected heterozygosity (*H*_o_ and *H*_e_), F-statistics (*F*_IS_ and *F*_ST_), relatedness (*r*), and pairwise Nei’s genetic distance were calculated in GenAlEx version 6.5 [16]. Population structure analyses, such as analysis of molecular variance (AMOVA) and principal coordinate analysis (PCoA) were estimated in GenAlEx version 6.5 [16], discriminant analysis of principal components (DAPC) was performed using package ADEGENET 2.0 [17] in R version 4.1.2 [18], and STRUCTURE analysis was performed in STRUCTURE version 2.3 [19] following Budi et al [10]. The Wilcoxon signed-rank test for detecting recent population bottlenecks was performed using a two-phased mutation model (TPM) and stepwise mutation model (SMM) to assess the probability of excess heterozygosity due to small sample sizes. Tests for relative long-term bottleneck events based on *M* ratio were performed following Budi et al [10]. To investigate the genetic selective sweeps in PDH and SD chickens, a selective sweep analysis was performed as described previously [10]. *H*_e_ and *F*_IS_ values of populations were plotted for each microsatellite locus (28 loci in total). High *F*_IS_ and low *H*_e_ values reflect sweeping or purifying selection signatures, whereas low *F*_IS_ and high *H*_e_ values indicates neutral or balanced selection.

To examine the occurrence of genetic exchange between PDH and SD chicken populations based on the microsatellite genotyping dataset, Bayesian interference analysis implemented in BayesAss version 3.0.5 [20] which is commonly used to determine recent migration rates between populations was performed. The Markov Chain Monte Carlo (MCMC) analysis was conducted for 10 million generations after a burn in a period of 1 million generations and sampled every 100 generations. The mixing parameters associated with migration rates (*m*), allele frequencies (*a*), and inbreeding coefficients (*f*) were optimized to satisfy 20% to 60% posterior distribution acceptance rates according to the recommended guideline [20]. Similarly, to assess the historical genetic exchange between PDH and SD chicken populations, migration rates between the populations and their effective population sizes were estimated by Bayesian analysis using MIGRATE-N version 4.4.3 [21,22]. In these analyses, uniform prior distributions were used for the basic microsatellite model, and 5,000 steps were recorded every 100 generations using the MCMC procedure. The first 100,000 generations were discarded as burn-ins. Estimates were calculated for the mutation-scaled immigration rate (*M*) and mutation-scaled population size (*Θ*). The number of individuals entering populations (*N*_m_) was calculated, and the presence of gene flow between populations in the past was determined using the formula *N*_mi–>j_ = Θ_j_*M_i–>j_/4, where *N*_mi–>j_ represents the effective number of immigrants or gene flow rate from population i to population j per generation. Circos (version 0.69–8) was used to visualize the genetic connectivity among populations [23]. The genotypic data generated in this study have been stored in the Dryad digital repository dataset (https://doi.org/10.5061/dryad.hhmgqnkm0, accessed on 15 January 2024).

### Investigation of the genetic origins of Samae Dam and Pradu Hang Dam chickens

The genetic origins of PDH and SD chickens were investigated using the microsatellite genotyping data of chickens available under the SCBP (https://www.sci.ku.ac.th/scbp/; https://datadryad.org/stash/share/x2qlPmboMgCROXO8, accessed on January 15, 2024), including red junglefowl and indigenous and local chicken breeds in Thailand. Two populations of SD chickens and five populations of PDH chickens were treated as separate data sets and compared the data with those of other indigenous and local chicken breeds, as well as with red junglefowl, which was considered a different data set. Pairwise genetic distances between populations and clustering analyses using PCoA, DAPC, and STRUCTURE were performed as described previously [10].

## RESULTS

### Genetic diversity of the Pradu Hang Dam and Samae Dam chicken populations based on microsatellite genotyping data

A total of 370 alleles were observed in the five populations of PDH chickens, with the mean number of alleles per locus being 4.086±0.312, whereas 179 alleles were found in the two populations of SD chickens, whose mean number of alleles per locus was 3.607±0.349 (Supplementary Table S3). All allelic frequencies showed a significant departure from the Hardy–Weinberg equilibrium of the population in both PDH and SD chicken populations, with evidence for linkage disequilibrium (Supplementary Tables S4–S10). Null alleles were frequently found for the MCW0034, MCW0014, MCW 0067, ADL0112, and MCW0069 loci; nevertheless, all the markers were used with no distinction for estimating the genetic diversity. Three populations of PDH chickens (PDH3, PDH4, and PDH5) and two populations of SD chickens each exhibited negative *F* values, whereas the other two populations of PDH (PDH1 and PDH2), exhibited positive *F* values. The PIC of all PDH and SD chicken populations ranged from 0.137 to 0.652, whereas the Shannon’s information index (*I*) was from 0.061 to 1.442. The mean *H*_o_ and *H*_e_ values were 0.591±0.052 and 0.594±0.030, respectively, for PDH chickens, and 0.668±0.050 and 0.503±0.036, respectively, for SD chickens. The mean *AR* value of PDH chickens and SD chickens were 3.771±1.471 and 2.526±0.873, respectively. The standard genetic diversity indices are summarized in Table 1. All pairwise differences in *H*_o_ and *H*_e_ values between PDH and SD chicken populations were not significant, except for the PDH3 and SD1 populations (Supplementary Table S11).

The mean pairwise *r* values in PDH and SD chickens were −0.013±0.070 and −0.022±0.106, respectively, whereas the *F*_IS_ values were −0.001±0.029 and −0.374±0.060, respectively (Supplementary Tables S12 to S20). AMOVA revealed that genetic variation accounted for 0% of the total variance within the populations of PDH chickens, whereas it accounted for 8% within the populations of SD chickens, and 24% among the populations of PDH chickens, and 29% among the populations of SD chickens, (Supplementary Table S21). Nei’s genetic distances values were ranged from 0.301 to 2.549 among the five populations of PDH chickens, and 1.236 between the two populations of SD chickens (Supplementary Table S22). Pairwise differences (*F*_ST_) based on microsatellite data ranged from 0.043 to 0.440 among populations in PDH and SD chickens (Supplementary Table S23). The Wilcoxon signed-rank tests generated the values of 0.582 and 0.973 under SMM and a TPM for PDH1 (normal L-shaped distribution), 0.493 and 0.537 for PDH3 (normal L-shaped distribution), and 0.695 and 0.327 for PDH4 (normal L-shaped distribution), 0.711 and 0.779 for SD1 (normal L-shaped distribution), and 0.425 and 0.248 for SD2 (shifted mode), respectively. The Wilcoxon signed-rank test could not be calculated for PDH2 and PDH5 due to their small sample sizes. The *M* ratios of all populations were below 0.68, reflecting a historical decline of populations [24] (Table 1).

**Figure 1:**
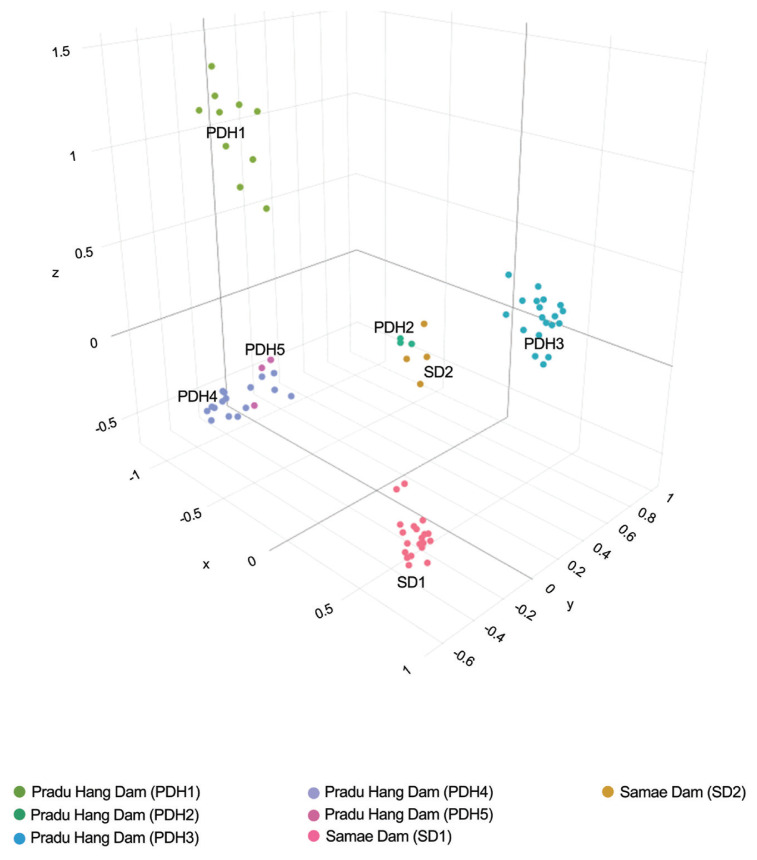
Principal coordinate analysis (PCoA) of Pradu Hang Dam chickens derived from Phitsanulok 1 (PDH1), Phitsanulok 2 (PDH2), Chiang Mai (PDH3), Nakhon Pathom (PDH4), and Nonthaburi (PDH5) populations, and Samae Dam chickens derived from Department of Livestock Uthai Thani (SD1) and Sanhawat Farm Uthai Thani (SD2) populations based on the genotyping data of 28 microsatellite loci. Each population is represented with a different color, and each point represents an individual.

**Figure 2:**
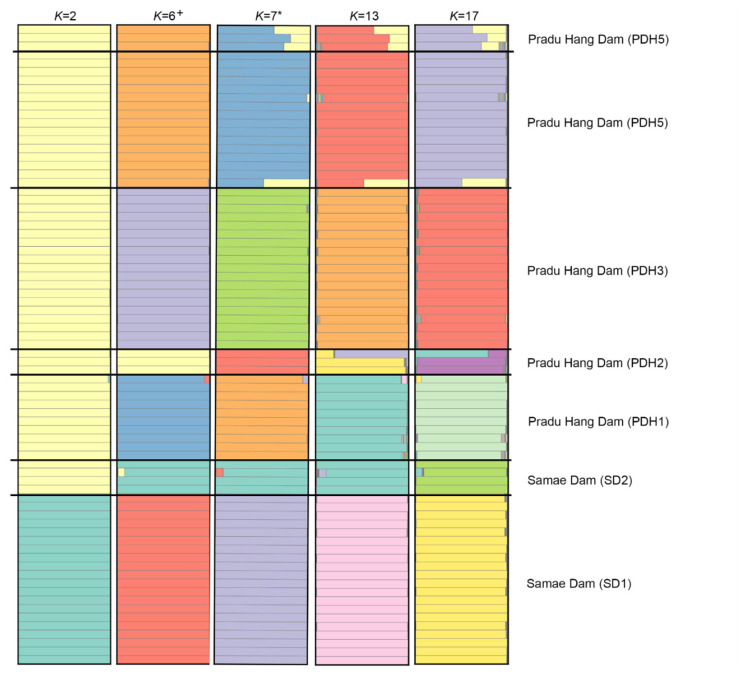
Population structures of Pradu Hang Dam chickens derived from Phitsanulok 1 (PDH1), Phitsanulok 2 (PDH2), Chiang Mai (PDH3), Nakhon Pathom (PDH4), and Nonthaburi (PDH5) populations, and Samae Dam chickens derived from Department of Livestock Uthai Thani (SD1) and Sanhawat Farm Uthai Thani (SD2) populations based on the genotyping data of 28 microsatellite loci. Thick horizontal black lines indicate the boundaries between populations. Each horizontal bar represents an individual, with its color proportion constituting the posterior probability of assignment to genetic clusters. The plus symbol indicates the appropriate *K* value based on Evano’s Δ*K*, whereas the asterisk represents the appropriate *K* value based on ln P(*K*).

PCoA and DAPC classified five PDH chicken populations into four clusters, whereas two SD chicken populations were classified separately into two clusters (Figure 1; Supplementary Figure S1). A Bayesian clustering analysis with STRUCTURE showed that PDH and SD chickens exhibited different population structure patterns at various *K*-values (*K* = 2 to 25) (Figure 2; Supplementary Figure S2). Based on Evano’s Δ*K* values, the optimized structure pattern was assigned to six clusters at *K* = 6, whereas based on ln P(K), the optimized structure pattern found at *K* = 7. At *K* = 6, all populations exhibited different gene pool patterns, except for PDH4 and PDH5. Similarly, at *K* = 7 or more than 7, all populations, except for PDH4 and PDH5 showed different gene pool patterns. Genetic selective sweep analysis revealed neutral or balanced selection occurred in all populations of PDH and SD chickens, which was reflected by a relatively low *F*_IS_ coupled with a high *H*_e_ (Supplementary Figure S3). Recent genetic gene exchange estimated using BayesAss revealed that migration rates range were 0.699 to 0.923 within populations and 0.0123 to 0.134 among populations in PDH and SD chickens. The asymmetric mutation rate (*M*) derived from MIGRATE-N ranged from 7.000 to 777.670, with the highest value observed from PDH3 to PDH2. The median scaled mutation rates (*Θ*) ranged from 0 (PDH4) to 0.099 (PDH1) (Supplementary Table S25). A diverse range of *N*_m_ values (0 to 0.501) was observed in PDH and SD populations (Supplementary Table S26).

### Genetic differences among Pradu Hang Dam and Samae Dam chickens, red junglefowl, and other indigenous and local chicken breeds in Thailand

Multiple population clusters were observed based on the PCoA and DAPC results (Supplementary Figure S4–S5). The PDH and SD chickens tended to cluster together with indigenous and local chicken breeds and red junglefowl. The gene pool patterns of PDH and SD chickens were compared with those of the indigenous and local chicken breeds and red junglefowl in Thailand using the dataset of microsatellite genotypes in our previous studies (SCBP, https://www.sci.ku.ac.th/scbp/). STRUCTURE analysis revealed the highest posterior probability with one peak at *K* = 2 based on Evano’s Δ*K*, whereas the mean ln P(*K*) showed a different peak at *K* = 25 (Supplementary Figure S6 to S7). At *K* = 2, the two populations of SD chickens shared the similar gene pool with all PDH populations, except for PDH1 population. The peak at a high *K* value (*K* = 25) indicated the presence of multiple clusters in SD and PDH populations. The SD2 population shared a partial gene pool with red junglefowl (*G. gallus gallus*) derived from Roi Et and Sisaket, whereas the SD1 population partially shared the gene pool with those of the PDH3, PDH1, and Lueng Hang Khao, Chee, Khaew Paree, and Decoy population (Supplementary Figure S6). The PDH4 and PDH5 populations shared similar gene pools with those of the Lao Pa Koi, Prama, Wein Chang, Chee Fah (Mae Hong Son), Fah Luang (Mae Hong Son), Mae Hong Son, and Dong Tao (Udon Thani) population. The PDH2 population showed a distinctive gene pool compared to other chicken populations. Additionally, a small portion of the gene pool of PDH2 population was observed in SD2 and PDH3 populations. The gene pools of SD and PDH populations were closely related to those of other indigenous and local chicken breeds and red junglefowl in Thailand, and no detectable genetic selective sweeps were observed in any chicken breeds and red junglefowl.

## DISCUSSION

To address the argument about the origins and genetic assignment of PDH and SD chickens, comparison of their genetic diversity and structure was considered. It is also required to evaluate their values as genetic resources [10]. The microsatellite genotyping data showed that the number of alleles per loci ranged from 1.00 to 16.00 (average, 3.95±0.16), and their *F*_ST_ values ranged from 0.043 to 0.440 (average, 0.287±0.083) in PDH and SD chickens. Notably, the clustering success rate of over 90% was attained for more than 15 individuals per population in at least 15 highly variable microsatellite DNA markers [25]. If the *F*_ST_ values exceed 0.10 for more than 20 microsatellite DNA markers in STRUCTURE analysis, the genetic assignment of populations is almost 100% accuracy irrespective of the frequencies of null alleles [26]. Therefore, our genetic clustering data of PDH and SD chickens deemed to be accurate [26], despite some populations having fewer than 10 individuals. This reflected the actual situation, since farmers can only provide information based on the remaining stock. Lower potential for subpopulation (negative *F* value) was observed in most populations of PDH and SD chickens [27], although high genetic diversity was observed. This was also confirmed by the low inbreeding coefficient values in PDH and SD chickens. This suggests that PDH and SD chickens are maintained in an efficient way for managing low levels of inbreeding [28], even if the number of individuals is small in each population. Furthermore, these data confirmed that PDH and SD chicken populations analyzed in this study may have experienced only minor bottlenecks in the process of breeding and the samples were collected without bias for testing the genetic diversity of the two chicken breeds.

### Factors that separate Pradu Hang Dam and Samae Dam chickens as different breeds or varieties

Population structure analysis using STRUCTURE revealed that PDH and SD chicken populations are separated into several different clusters at *K* = 6 and *K* = 7, which was consistent with the results by PCoA and DAPC analyses. The SD1 chicken population exhibited a distinct gene pool that was different from those of the SD2 chicken population and PDH chickens through all *K*-values, which suggests that the two SD chicken populations have distinct genetic origins [10]. The SD1 population exhibited a strikingly gene pool pattern similar to those of PDH1 and PDH3 populations. Genetic exchange between the SD1 population and PDH chickens was confirmed using gene flow analysis based on Bayesian interference analysis of recent migration rates between populations (Supplementary Figure S8) [29]. This suggests that PDH and SD chickens may have been originated from the same breed known as the PDH chicken breed. PDH and SD chickens have similar body sizes and weight; however, they can be distinguished by the coloration of their eyes, face, combs, and hackles. PDH chickens have red eyes, faces, and combs, and red-brown colored hackles, whereas SD chickens have typically black eyes, faces, and combs, and brownish-oak colored hackles [4,5,7]. Therefore, SD chickens have been considered to be a variety of the PDH chicken breed. Long-term selective breeding. focusing on the production of high quality of poultry products and ease of breeding, has led to the improvement of the PDH chicken breed. The sub-group that differs in either external morphologies or plumage color or both, within a breed is designated as a “variety.” These variations are found in PDH chickens, such as the Pradu Hang Dam Chiang Mai and Pradu Hang Dam KKU55 populations [30,31]. Recent selection biases have resulted in the homogenization of morphological features of the PDH chicken breed. This led to the rapid reduction in genetic diversity and acceleration of genetic similarity between varieties as observed in SD chickens.

Notably, the SD1 and SD2 chickens were probably derived from different origins and evolved into distinct subgroups as different sub-varieties. The sub-group with specific desired traits improved by selective breeding, is referred to as “strain” [32]. The SD1 chicken population, which was raised for meat production, was genetically related to PDH chickens studied here, which were improved for meat production and cockfighting. Their gene pools were partially shared with that of the SD2 chicken population, which was improved for ornamental competition with red junglefowl derived from Roi Et and Si Sa Ket, which is why there is a genetic footprint from red junglefowl in SD chickens. Genetic variability in the variety of SD chickens may be influenced by the breeders’ purposes, shaping the selection and domestication processes. However, the microsatellite DNA markers used in this study could not identify the genetic traits linked to them in each strain or population. High-throughput genotyping, such as double digest restriction-site associated (ddRAD) or whole-genome sequencing, would be alternative options for the in-depth identification of genes or genetic markers associated with the chicken breed- or variety-specific biological features.

### Significance of conservation and utilization of indigenous and local chickens in the local community

Many chicken breeds have been developed for entertainment because of their specific morphological, external and behavioral features, such as body shapes and sizes, combs, wattles, tail feathers, plumage colors, crowing sounds, and rooster’s fighting behavior. However, according to the annual report of the Food and Agricultural Organization (FAO), 21% of 1,729 chicken breeds are on the verge of extinction [33]. These alarming statistics present the critical need to identify the factors that drive genetic erosion and extinction risks in chicken breeds, including the indigenous and local breeds and to take appropriate actions. Loss of genetic diversity within and between breeds, that is, genetic homogenization of chicken breeds, can occur due to various factors, such as economic pressure to make profit, ecological changes, social shifts, and government policies. To conserve the genetic diversity of chicken breeds effectively, determining these driving factors by population genetics analyses of genetic diversity, population structures, and population movement monitoring and taking appropriate actions are required. In the SCBP at Kasetsart University, the efforts to protect endangered breeds are enforced by collecting and preserving DNA resources of indigenous and local chicken breeds and red junglefowl in collaboration with local communities in Thailand. An alternative approach would be to emphasize the cultural and economic value of indigenous and local chicken breeds in local communities and encourage their active involvement in conservation. The SD chicken varieties, which have unique phenotypes and cultural significance are on the brink of extinction. Therefore, preserving them would not only ensure a stable supply to local communities but also potentially contribute to maintaining sustainable agriculture through the production of indigenous and local chicken breeds. Genetic resources of indigenous and local chicken breeds have the potential for meeting customers’ demands of breeding in the future, which would generate profits for both farmers and consumers. Employment generation and income distribution in local communities can be facilitated by implementing the strategies of effective conservation of useful chicken resources to support sustainable agriculture.

## CONCLUSION

Genetic monitoring using microsatellite DNA markers was performed to assess the genetic characteristics and diversity of indigenous Thai chickens, PDH and SD chickens. Genetic cluster analyses suggest that SD chickens are genetically positioned as a variety of the PDH chicken breed, which exhibits the phenotypic characteristics that are similar to those of SD chickens. Two SD chicken populations (SD1 and SD2) were recognized as different strains based on the difference of genetic characteristics between their genetic characteristics, indicating that their genetic origins were different. An action plan is being developed to preserve the SD chicken variety and ensure a stable supply to the local community in Uthai Thani Province, Thailand, for utilization.

## Figures and Tables

**Table 1:** Genetic diversity of five populations of 51 individuals of Pradu Hang Dam chickens and 24 individuals of two populations of Samae Dam chickens estimated with 28 microsatellite loci

Breed	Population		*N*_a_	*AR*	*N*_ea_	*I*	*H*_o_	*H*_e_	*M* ratio	PIC	*F*
Pradu Hang Dam	Phitsanulok 1	Mean	3.821	3.569	2.538	1.029	0.509	0.566	0.229	0.508	0.119
		SE	0.252	1.142	0.160	0.061	0.040	0.025	0.068	0.137	0.062
	Phitsanulok 2	Mean	2.964	2.507	2.632	0.934	0.429	0.546	0.184	0.480	0.217
		SE	0.233	0.759	0.207	0.083	0.074	0.039	0.059	0.204	0.126
	Chiang Mai	Mean	6.250	6.041	3.930	1.442	0.756	0.696	0.213	0.652	−0.109
		SE	0.553	2.712	0.360	0.083	0.042	0.024	0.064	0.138	0.064
	Nakhon Pathom	Mean	4.464	4.250	3.069	1.177	0.643	0.617	0.172	0.566	−0.046
		SE	0.335	1.583	0.264	0.076	0.040	0.030	0.046	0.158	0.044
	Nonthaburi	Mean	2.929	2.488	2.500	0.925	0.619	0.547	0.194	0.479	−0.131
		SE	0.185	0.667	0.175	0.066	0.063	0.030	0.055	0.163	0.095
	Total	Mean	4.086	3.771	2.934	1.101	0.591	0.594	0.199	0.537	0.010
		SE	0.312	1.471	0.233	0.074	0.052	0.030	0.058	0.160	0.078
Samae Dam	Department of livestock Uthaithani province	Mean	4.964	3.144	2.994	1.193	0.929	0.627	0.215	0.567	−0.491
		SE	0.523	1.301	0.195	0.075	0.037	0.025	0.084	0.142	0.048
	Sanhawat Farm Uthaithani	Mean	2.250	1.909	1.897	0.619	0.408	0.379	0.245	0.326	−0.119
		SE	0.175	0.649	0.151	0.081	0.064	0.047	0.080	0.218	0.109
	Total	Mean	3.607	2.526	2.446	0.906	0.668	0.503	0.230	0.447	−0.305
		SE	0.349	0.873	0.173	0.078	0.050	0.036	0.082	0.179	0.079

*N*_a_, number of alleles; *AR*, allelic richness; *N_ea_*, number of effective alleles; *I*, Shannon’s information index; *H*_o_, observed heterozygosity; *H*_e_, expected heterozygosity; *M* ratio, the mean ratio of the number of alleles to the range in allelic size; PIC, polymorphic information content; *F*, fixation index.

## SUPPLEMENTARY MATERIAL

**Supplementary Figure S1**. Discriminant analysis of principal components (DAPC) of Pradu Hang Dam chickens derived from Phitsanulok 1 (PDH1), Phitsanulok 2 (PDH2), Chiang Mai (PDH3), Nakhon Pathom (PDH4), and Nonthaburi (PDH5) populations, and Samae Dam chickens derived from Department of Livestock Uthai Thani (SD1) and Sanhawat Farm Uthai Thani (SD2) populations based on the genotyping data of 28 microsatellite loci.

**Supplementary Figure S2**. Different patterns of population structures of Pradu Hang Dam (PDH) and Samae Dam (SD) chickens based on the genotyping data of 28 microsatellite loci generated by the model-based Bayesian clustering algorithms implemented in STRUCTURE.

**Supplementary Figure S3**. Mapping of expected heterozygosity (He) and inbreeding coefficients (FIS).

**Supplementary Figure S4**. Principal coordinate analysis (PCoA) of Pradu Hang Dam derived from Phitsanulok 1 (PDH1), Phitsanulok 2 (PDH2), Chiang Mai (PDH3), Nakhon Pathom (PDH4), Nonthaburi (PDH5) populations, and Samae Dam derived from Department of Livestock Uthai Thani (SD1), and Sanhawat Farm Uthai Thani (SD2) populations with indigenous and local chicken breeds, and red junglefowl in Thailand that are deposited in the Siam Chicken Bioresource Consortium (SCBP) database based on genotyping data of 28 microsatellite loci.

**Supplementary Figure S5**. Discriminant analysis of principal components (DAPC) of Pradu Hang Dam chickens derived from Phitsanulok 1 (PDH1), Phitsanulok 2 (PDH2), Chiang Mai (PDH3), Nakhon Pathom (PDH4), and Nonthaburi (PDH5) populations, and Samae Dam chickens derived from Department of Livestock Uthai Thani (SD1) and Sanhawat Farm Uthai Thani (SD2) populations, and other indigenous and local chicken breeds and red junglefowl in Thailand that are deposited in the Siam Chicken Bioresource Consortium (SCBP) database based on genotyping data of 28 microsatellite loci.

**Supplementary Figure S6**. The population structure of Pradu Hang Dam derived from Phitsanulok 1 (PDH1), Phitsanulok 2 (PDH2), Chiang Mai (PDH3), Nakhon Pathom (PDH4), Nonthaburi (PDH5) populations, and Samae Dam derived from Department of Livestock Uthai Thani (SD1), and Sanhawat Farm Uthai Thani (SD2) populations with indigenous and local chicken breeds, and red junglefowl in Thailand that are deposited in the Siam Chicken Bioresource Consortium (SCBP) database based on genotyping data of 28 microsatellite loci.

**Supplementary Figure S7**. Population structures of Pradu Hang Dam (PDH) chickens, Samae Dam (SD) chickens, and other indigenous and local chicken breeds and red junglefowl in Thailand that are deposited in the Siam Chicken Bioresource Consortium (SCBP) database based on 28 microsatellite loci generated by model-based Bayesian clustering algorithms implemented in STRUCTURE.

**Supplementary Figure S8**. Schematic representation of the current migration rates among populations of Pradu Hang Dam chickens derived from Phitsanulok 1 (PDH1), Phitsanulok 2 (PDH2), Chiang Mai (PDH3), Nakhon Pathom (PDH4), and Nonthaburi (PDH5) populations, and Samae Dam chickens derived from Department of Livestock Uthai Thani (SD1) and Sanhawat Farm Uthai Thani (SD2) populations.

**Supplementary Table S1**. Detailed information of Pradu Hang Dam and Samae Dam chicken specimens

**Supplementary Table S2**. Microsatellite primers, primer sequences, and fragment sizes obtained from Pradu Hang Dam and Samae Dam chicken specimens

**Supplementary Table S3**. Genetic diversity of 75 individuals of Pradu Hang Dam and Samae Dam chicken breeds based on 28 microsatellite loci

**Supplementary Table S4**. Pairwise differentiation of linkage disequilibrium of Pradu Hang Dam chickens derived from Phitsanulok 1 population (PDH1) based on 28 microsatellite loci

**Supplementary Table S5**. Pairwise differentiation of linkage disequilibrium of Pradu Hang Dam chickens derived from Phitsanulok 2 population (PDH2) based on 28 microsatellite loci

**Supplementary Table S6**. Pairwise differentiation of linkage disequilibrium of Pradu Hang Dam chickens derived from Chiang Mai population (PDH3) based on 28 microsatellite loci

**Supplementary Table S7**. Pairwise differentiation of linkage disequilibrium of Pradu Hang Dam chickens derived from Nakhon Pathom population (PDH4) based on 28 microsatellite loci

**Supplementary Table S8**. Pairwise differentiation of linkage disequilibrium of Pradu Hang Dam chickens derived from Nonthaburi population (PDH5) based on 28 microsatellite loci

**Supplementary Table S9**. Pairwise differentiation of linkage disequilibrium of Samae Dam chickens derived from the Department of Livestock, Uthai Thani (SD1), based on 28 microsatellite loci

**Supplementary Table S10**. Pairwise differentiation of linkage disequilibrium of Samae Dam chickens derived from the Sanhawat Farm, Uthai Thani (SD2), based on 28 microsatellite loci

**Supplementary Table S11**. Comparison of the observed and expected heterozygosity of Pradu Hang Dam and Samae Dam chicken based on 28 microsatellite loci

**Supplementary Table S12**. Inbreeding coefficients (FIS) of Pradu Hang Dam (n = 10) derived from Phitsanulok 1 (PDH1)

**Supplementary Table S13**. Inbreeding coefficients (FIS) of Pradu Hang Dam (n = 3) derived from Phitsanulok 2 (PDH2)

**Supplementary Table S14**. Inbreeding coefficients (FIS) of Pradu Hang Dam (n = 19) derived from Chiang Mai (PDH3)

**Supplementary Table S15**. Inbreeding coefficients (FIS) of Pradu Hang Dam (n = 16) derived from Nakhon Pathom (PDH4)

**Supplementary Table S16**. Inbreeding coefficients (FIS) of Pradu Hang Dam (n = 3) derived from Nonthaburi (PDH5)

**Supplementary Table S17**. Inbreeding coefficients (FIS) of Samae Dam chickens (n = 20) derived from Department of Livestock, Uthai Thani (SD1)

**Supplementary Table S18**. Inbreeding coefficients (FIS) of Samae Dam chickens (n = 4) derived from Sanhawat Farm, Uthai Thani (SD2)

**Supplementary Table S19**. Pairwise genetic relatedness (r) amongst Pradu Hang Dam chicken populations derived from Phitsanulok 1 (PDH1), Phitsanulok 2 (PDH2), Chiang Mai (PDH3), Nakhon Pathom (PDH4), and Nonthaburi (PDH5)

**Supplementary Table S20**. Pairwise genetic relatedness (r) amongst Samae Dam chicken populations derived from the Department of Livestock, Uthai Thani (SD1) and the Sanhawat Farm, Uthai Thani (SD2)

**Supplementary Table S21**. Analysis of molecular variance (AMOVA) for Pradu Hang Dam (PDH) and Samae Dam (SD) chickens based on 28 microsatellite loci

**Supplementary Table S22**. Results of pairwise Nei’s genetic distance (D) among Pradu Hang Dam chicken derived from Phitsanulok 1 (PDH1), Phitsanulok 2 (PDH2), Chiang Mai (PDH3), Nakhon Pathom (PDH4), Nonthaburi (PDH5), and Samae Dam chicken derived from Department of Livestock Uthai Thani (SD1), and Sanhawat Farm Uthai Thani (SD2), based on 28 microsatellite loci

**Supplementary Table S23**. Pairwise population differentiation (FST) between Pradu Hang Dam chicken derived from Phitsanulok 1 (PDH1), Phitsanulok 2 (PDH2), Chiang Mai (PDH3), Nakhon Pathom (PDH4), Nonthaburi (PDH5), and Samae Dam chicken derived from Department of Livestock Uthai Thani (SD1), and Sanhawat Farm Uthai Thani (SD2) populations based on 28 microsatellite loci

**Supplementary Table S24**. Current migration rates among populations of Pradu Hang Dam chicken derived from Phitsanulok 1 (PDH1), Phitsanulok 2 (PDH2), Chiang Mai (PDH3), Nakhon Pathom (PDH4), Nonthaburi (PDH5), and Samae Dam chicken derived from Department of Livestock Uthai Thani (SD1), and Sanhawat Farm Uthai Thani (SD2) based on 28 microsatellite loci generated in BayesAss

**Supplementary Table S25**. Bayesian estimates of mutation-scaled effective population sizes (Θ) and asymmetric migration rates (M) among Pradu Hang Dam chicken derived from Phitsanulok 1 (PDH1), Phitsanulok 2 (PDH2), Chiang Mai (PDH3), Nakhon Pathom (PDH4), Nonthaburi (PDH5), and Samae Dam chicken derived from Department of Livestock Uthai Thani (SD1), and Sanhawat Farm Uthai Thani (SD2) based on 28 microsatellite loci generated in MIGRATE-N

**Supplementary Table S26**. Effective number of immigrants (Nm) from population i to population j per generation generated in MIGRATE-N
