# Supplementary material for: Samae Dam chicken: a variety of the Pradu Hang Dam breed revealed from microsatellite genotyping data
Source: Anim Biosci. 2024 Jun 25;37(12):2033–43. doi: 10.5713/ab.24.0161 (PMC11541018; doi:10.5713/ab.24.0161)
Supplement: Supplementary file 1 [file ab-24-0161-Supplementary-Fig-S1.pdf]

Supplementary Figures and Tables

Supplementary Figures

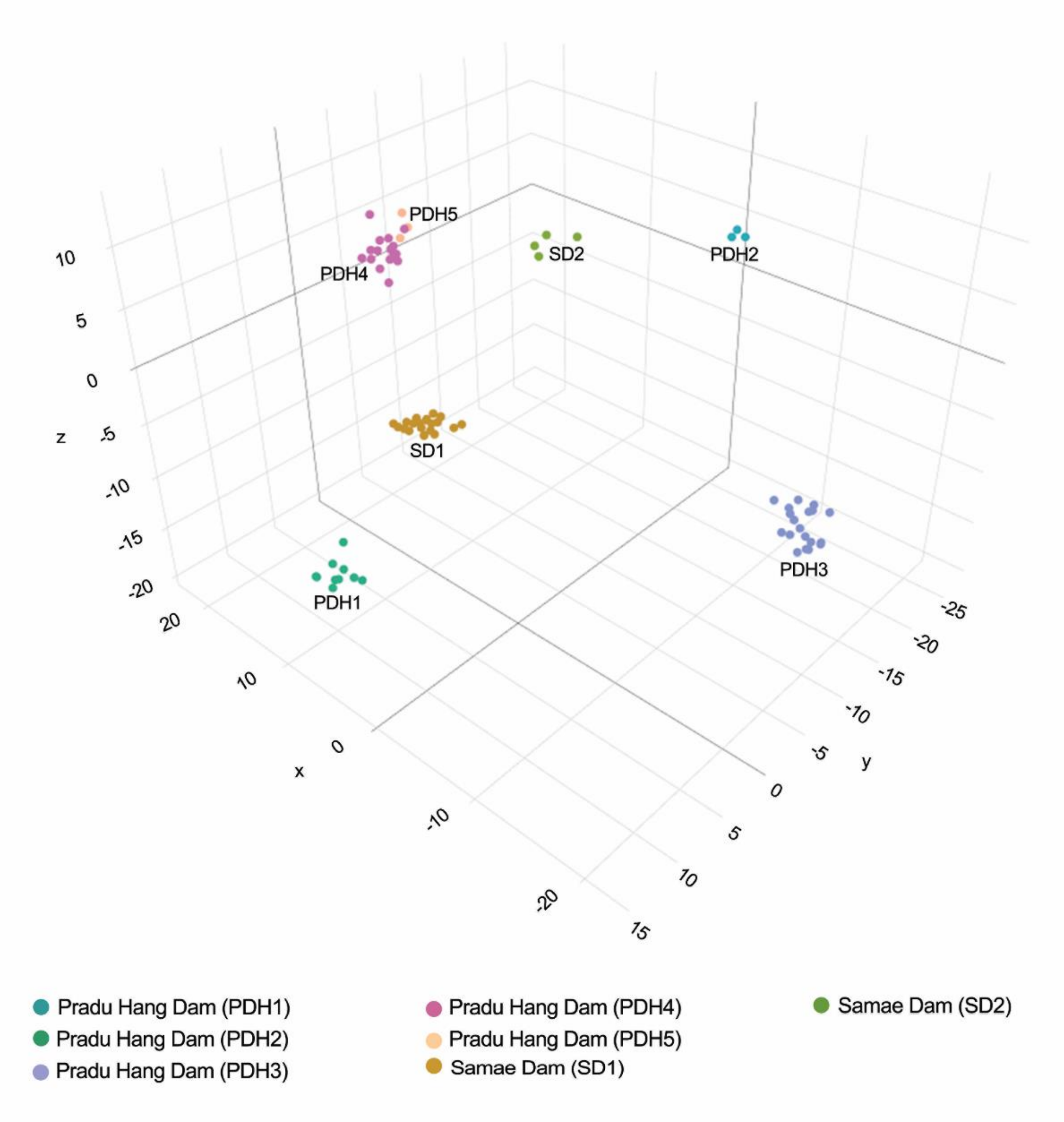

Figure S1. Discriminant analysis of principal components (DAPC) of Pradu Hang Dam chickens derived from Phitsanulok 1 (PDH1), Phitsanulok 2 (PDH2), Chiang Mai (PDH3), Nakhon Pathom (PDH4), and Nonthaburi (PDH5) populations, and Samae Dam chickens derived from Department of Livestock Uthai Thani (SD1) and Sanhawat Farm Uthai Thani (SD2) populations based on the genotyping data of 28 microsatellite loci. Each population is represented with a different color, and each point represents an individual.
