# Supplementary material for: Samae Dam chicken: a variety of the Pradu Hang Dam breed revealed from microsatellite genotyping data
Source: Anim Biosci. 2024 Jun 25;37(12):2033–43. doi: 10.5713/ab.24.0161 (PMC11541018; doi:10.5713/ab.24.0161)
Supplement: Supplementary file 2 [file ab-24-0161-Supplementary-Fig-S2.pdf]

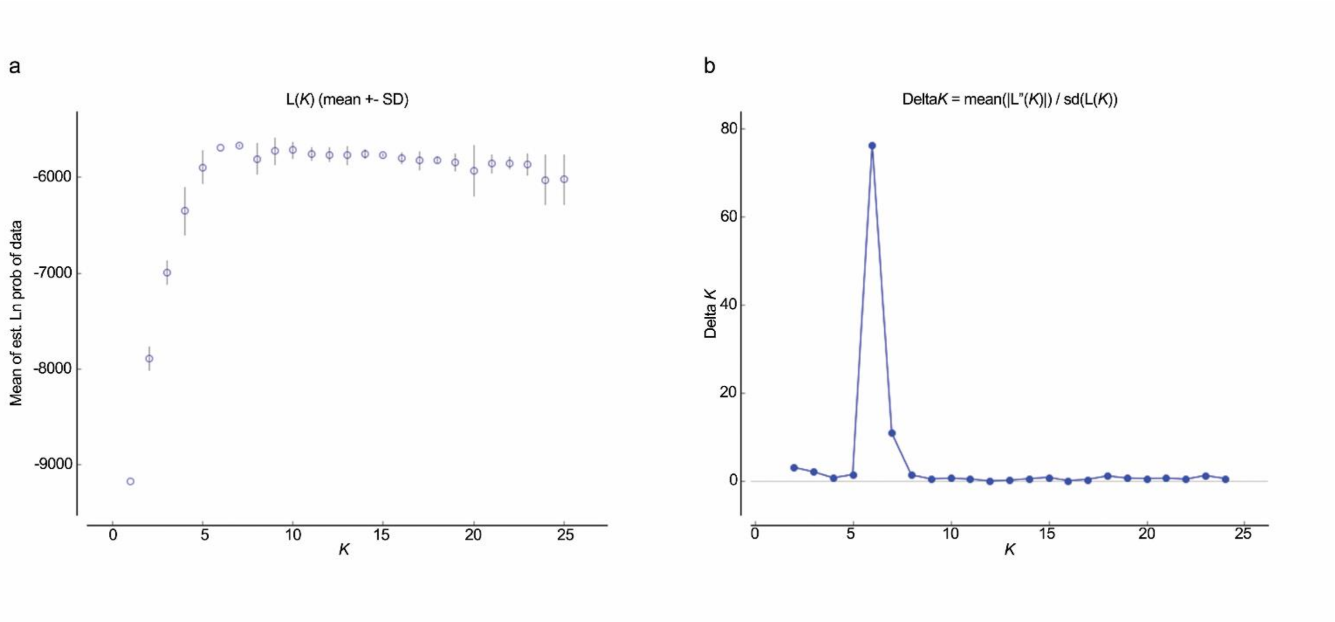

Figure S2. Different patterns of population structures of Pradu Hang Dam (PDH) and Samae Dam (SD) chickens based on the genotyping data of 28 microsatellite loci generated by the model-based Bayesian clustering algorithms implemented in STRUCTURE. (a) Plot based on ln P(K). (b) Plot based on Evano's ΔK.
