# Supplementary material for: Samae Dam chicken: a variety of the Pradu Hang Dam breed revealed from microsatellite genotyping data
Source: Anim Biosci. 2024 Jun 25;37(12):2033–43. doi: 10.5713/ab.24.0161 (PMC11541018; doi:10.5713/ab.24.0161)
Supplement: Supplementary file 4 [file ab-24-0161-Supplementary-Fig-S4.pdf]

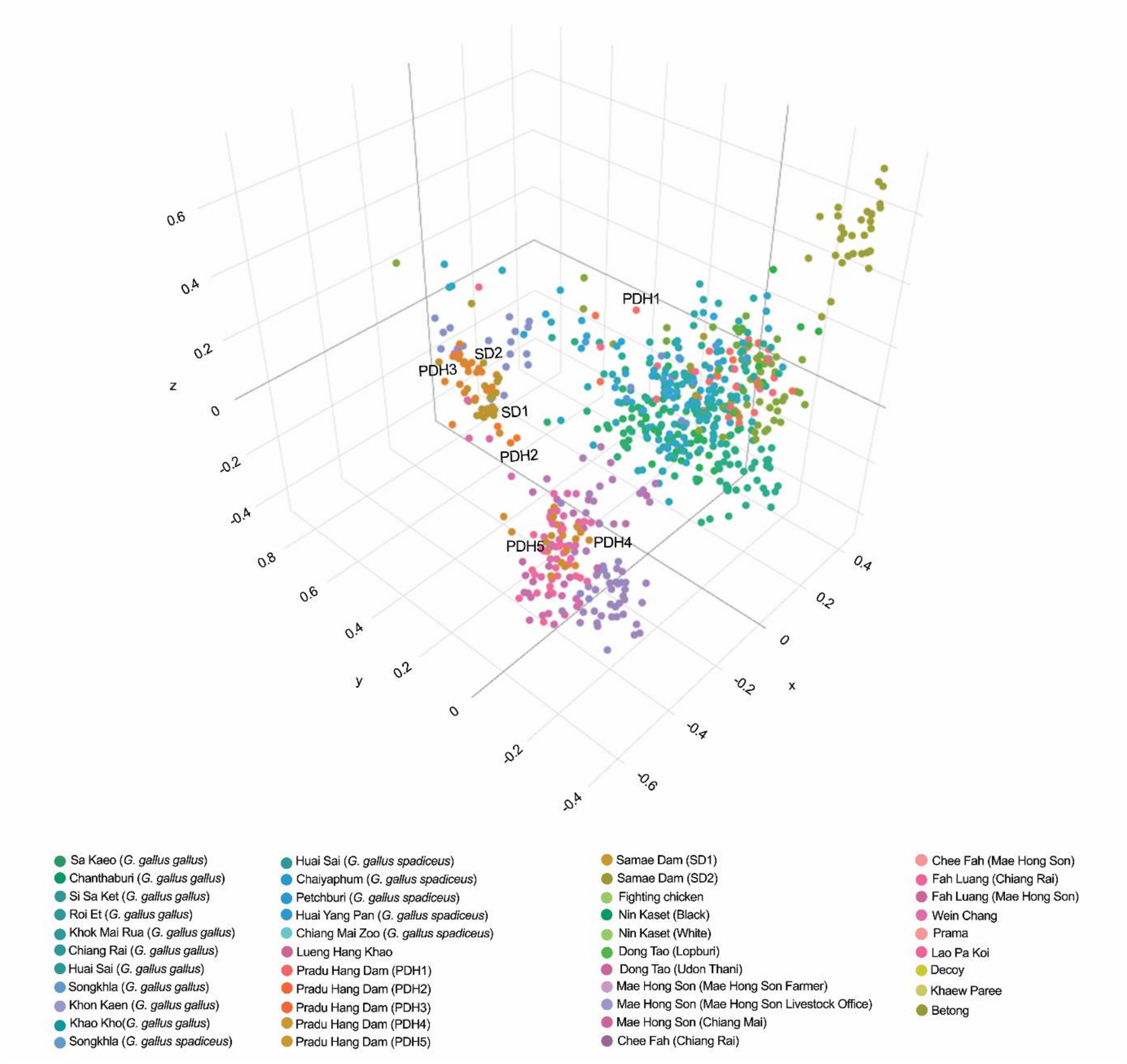

Figure S4. Principal coordinate analysis (PCoA) of Pradu Hang Dam derived from Phitsanulok 1 (PDH1), Phitsanulok 2 (PDH2), Chiang Mai (PDH3), Nakhon Pathom (PDH4), Nonthaburi (PDH5) populations, and Samae Dam derived from Department of Livestock Uthai Thani (SD1), and Sanhawat Farm Uthai Thani (SD2) populations with indigenous and local chicken breeds, and red junglefowl in Thailand that are deposited in the Siam Chicken Bioresource Consortium (SCBP) database based on genotyping data of 28 microsatellite loci. The assigned genetic clusters are represented by different colors, while symbols represent different individuals.
