# Supplementary material for: Samae Dam chicken: a variety of the Pradu Hang Dam breed revealed from microsatellite genotyping data
Source: Anim Biosci. 2024 Jun 25;37(12):2033–43. doi: 10.5713/ab.24.0161 (PMC11541018; doi:10.5713/ab.24.0161)
Supplement: Supplementary file 6 [file ab-24-0161-Supplementary-Fig-S6.pdf]

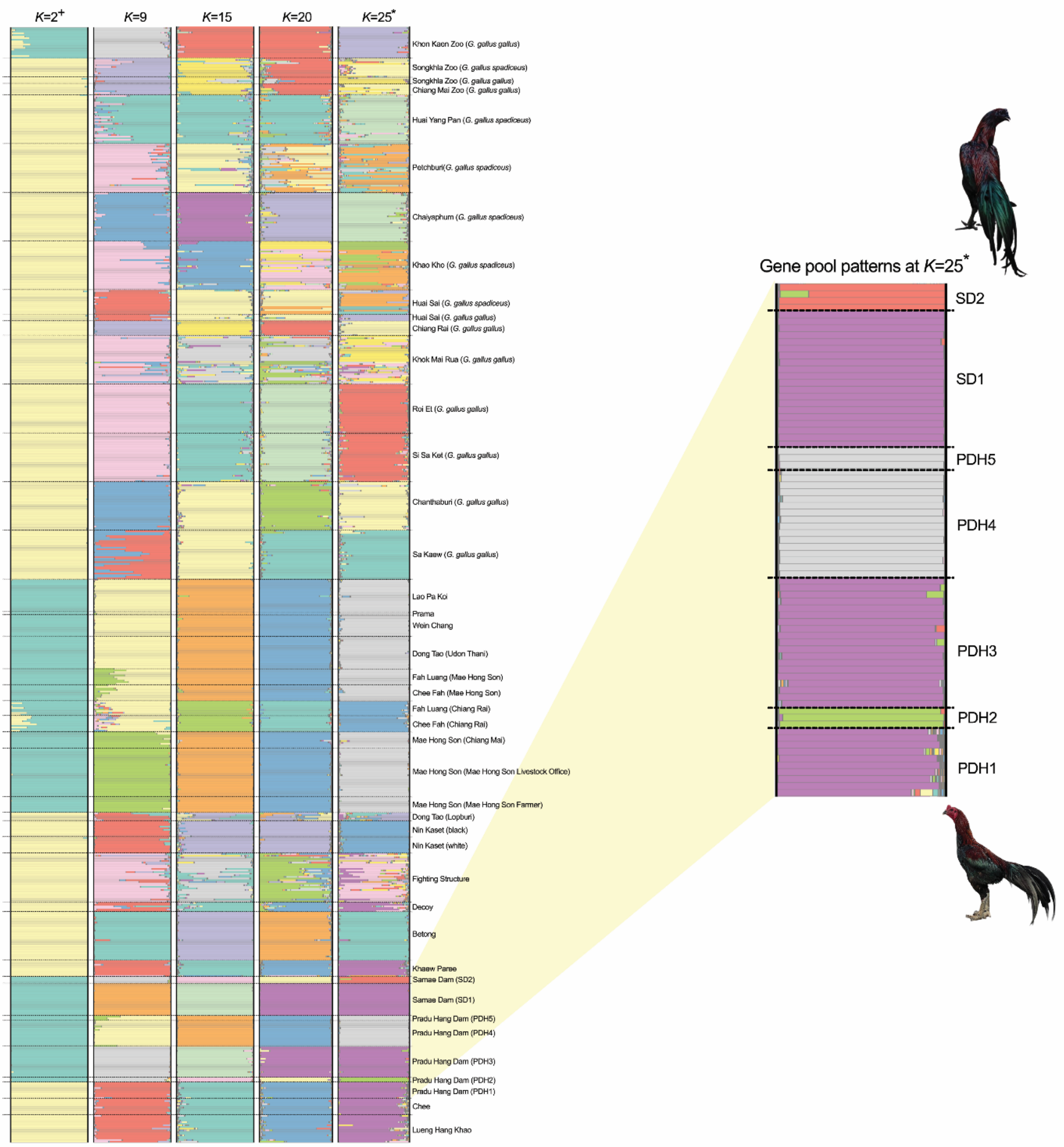

Figure S6. The population structure of Pradu Hang Dam derived from Phitsanulok 1 (PDH1), Phitsanulok 2 (PDH2), Chiang Mai (PDH3), Nakhon Pathom (PDH4), Nonthaburi (PDH5) populations, and Samae Dam derived from Department of Livestock Uthai Thani (SD1), and Sanhawat Farm Uthai Thani (SD2) populations with indigenous and local chicken breeds, and red junglefowl in Thailand that are deposited in the Siam Chicken Bioresource Consortium (SCBP) database based on genotyping data of 28 microsatellite loci. Each horizontal bar on the x-axis represents the proportion of membership (posterior probability) in each genetic cluster while the y-axis represents individuals. Black horizontal lines indicate the boundaries. The plus symbol indicates the appropriate K based on ln P(K) and the asterisk indicates the appropriate K based on Evano's ΔK.
