# Supplementary material for: Samae Dam chicken: a variety of the Pradu Hang Dam breed revealed from microsatellite genotyping data
Source: Anim Biosci. 2024 Jun 25;37(12):2033–43. doi: 10.5713/ab.24.0161 (PMC11541018; doi:10.5713/ab.24.0161)
Supplement: Supplementary file 7 [file ab-24-0161-Supplementary-Fig-S7.pdf]

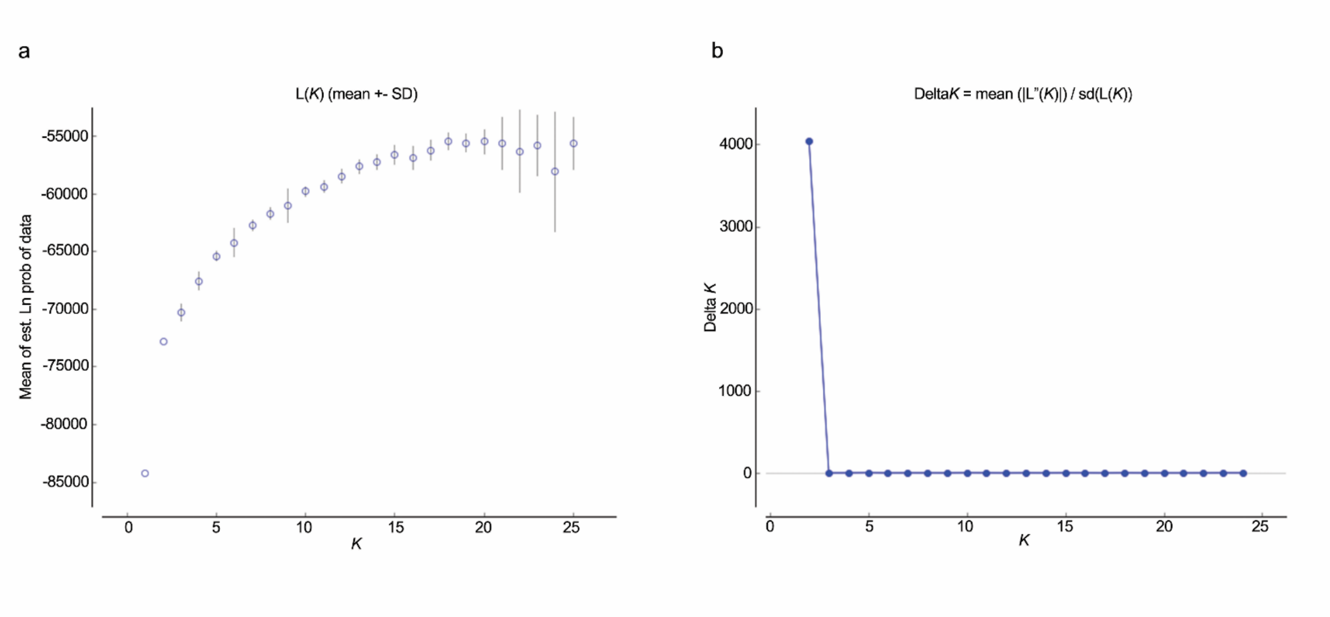

Figure S7. Population structures of Pradu Hang Dam (PDH) chickens, Samae Dam (SD) chickens, and other indigenous and local chicken breeds and red junglefowl in Thailand that are deposited in the Siam Chicken Bioresource Consortium (SCBP) database based on 28 microsatellite loci generated by model-based Bayesian clustering algorithms implemented in STRUCTURE. (a) Plot based on ln P(*K*). (b) Plot based on Evano's Δ*K*.
