# Supplementary material for: Samae Dam chicken: a variety of the Pradu Hang Dam breed revealed from microsatellite genotyping data
Source: Anim Biosci. 2024 Jun 25;37(12):2033–43. doi: 10.5713/ab.24.0161 (PMC11541018; doi:10.5713/ab.24.0161)
Supplement: Supplementary file 8 [file ab-24-0161-Supplementary-Fig-S8.pdf]

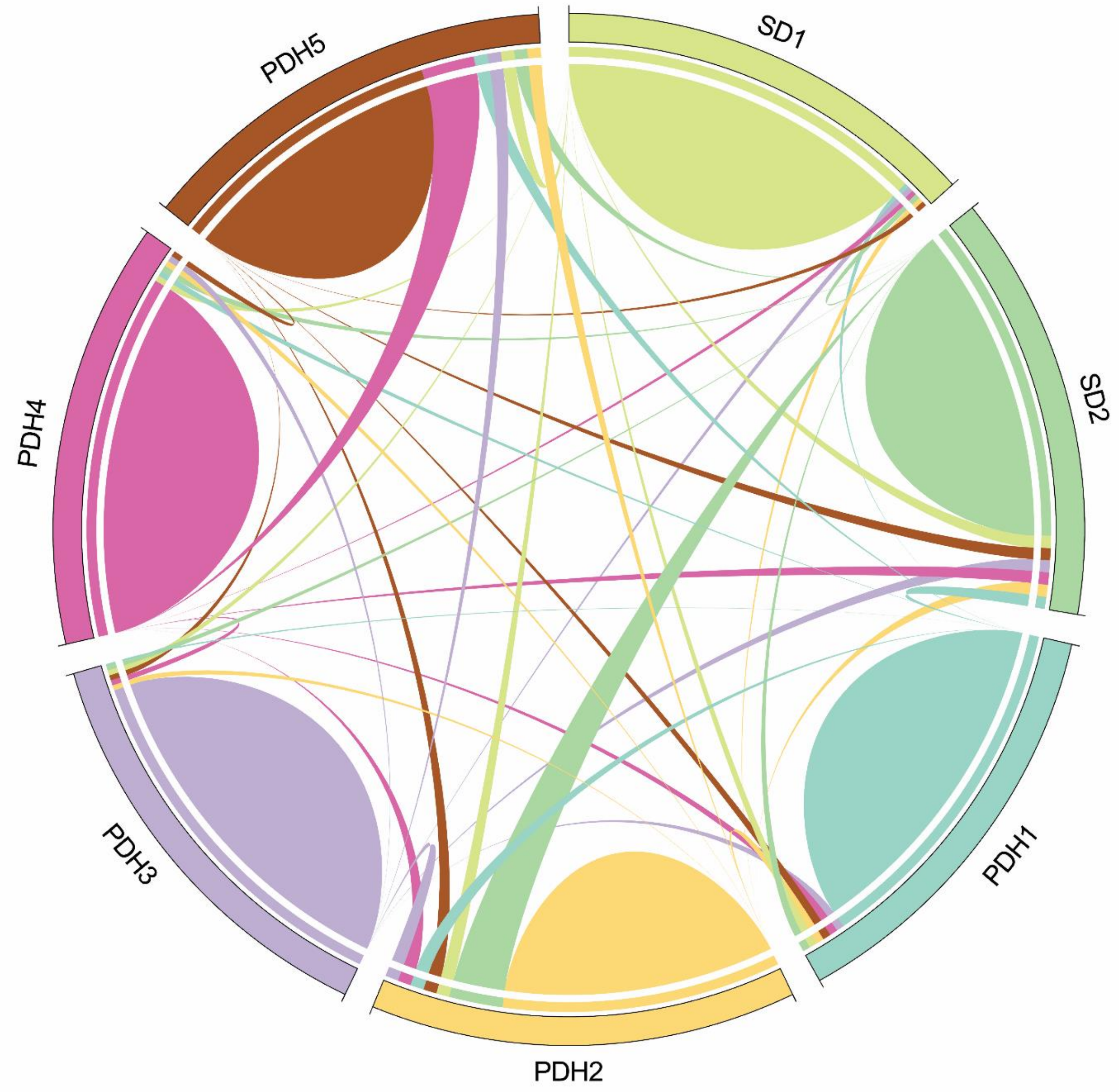

Figure S8. Schematic representation of the current migration rates among populations of Pradu Hang Dam chickens derived from Phitsanulok 1 (PDH1), Phitsanulok 2 (PDH2), Chiang Mai (PDH3), Nakhon Pathom (PDH4), and Nonthaburi (PDH5) populations, and Samae Dam chickens derived from Department of Livestock Uthai Thani (SD1) and Sanhawat Farm Uthai Thani (SD2) populations.
