# Supplementary material for: Samae Dam chicken: a variety of the Pradu Hang Dam breed revealed from microsatellite genotyping data
Source: Anim Biosci. 2024 Jun 25;37(12):2033–43. doi: 10.5713/ab.24.0161 (PMC11541018; doi:10.5713/ab.24.0161)
Supplement: Supplementary file 9 [file ab-24-0161-Supplementary-Table-S1.pdf]

Supplementary Tables

Table S1. Detailed information of Pradu Hang Dam and Samae Dam chicken specimens

No.	Breeds	Sample code	Population	Population Code
**1**	Samae Dam	SDM1	Department of livestock Uthai Thani province	SD1
**2**		SDM2	Department of livestock Uthai Thani province	
**3**		SDM3	Department of livestock Uthai Thani province	
**4**		SDM4	Department of livestock Uthai Thani province	
**5**		SDM5	Department of livestock Uthai Thani province	
**6**		SDM6	Department of livestock Uthai Thani province	
**7**		SDM7	Department of livestock Uthai Thani province	
**8**		SDM8	Department of livestock Uthai Thani province	
**9**		SDM9	Department of livestock Uthai Thani province	
**10**		SDM10	Department of livestock Uthai Thani province	
**11**		SDFM1	Department of livestock Uthai Thani province	
**12**		SDFM2	Department of livestock Uthai Thani province	
**13**		SDFM3	Department of livestock Uthai Thani province	
**14**		SDFM4	Department of livestock Uthai Thani province	
**15**		SDFM5	Department of livestock Uthai Thani province	
**16**		SDFM6	Department of livestock Uthai Thani province	
**17**		SDFM7	Department of livestock Uthai Thani province	
**18**		SDFM8	Department of livestock Uthai Thani province	
**19**		SDFM9	Department of livestock Uthai Thani province	
**20**		SDFM10	Department of livestock Uthai Thani province	
**21**	Samae Dam	SD1	Sanhawat Farm Uthai Thani	SD2
**22**		SD2	Sanhawat Farm Uthai Thani	
**23**		SD3	Sanhawat Farm Uthai Thani	
**24**		SD4	Sanhawat Farm Uthai Thani	
**25**	Pradu Hang Dam	PDH1	Phitsanulok 1	PDH1
**26**		PDH2	Phitsanulok 1	
**27**		PDH3	Phitsanulok 1	
**28**		PDH4	Phitsanulok 1	
**29**		PDH5	Phitsanulok 1	
**30**		PDH6	Phitsanulok 1	
**31**		PDH7	Phitsanulok 1	
**32**		PDH8	Phitsanulok 1	
**33**		PDH9	Phitsanulok 1	
**34**		PDH10	Phitsanulok 1	
**35**	Pradu Hang Dam	PDN1	Phitsanulok 2	PDH2
**36**		PDN2	Phitsanulok 2	
**37**		PDN3	Phitsanulok 2	
**38**	Pradu Hang Dam	PD1	Chiang Mai	PDH3
**39**		PD2	Chiang Mai	
**40**		PD3	Chiang Mai	
**41**		PD4	Chiang Mai	
**42**		PD5	Chiang Mai	
**43**		PD6	Chiang Mai	

**44**		PD7	Chiang Mai	
**45**		PD8	Chiang Mai	
**46**		PD9	Chiang Mai	
**47**		PD10	Chiang Mai	
**48**		PD11	Chiang Mai	
**49**		PD12	Chiang Mai	
**50**		PD13	Chiang Mai	
**51**		PD14	Chiang Mai	
**52**		PD15	Chiang Mai	
**53**		PD16	Chiang Mai	
**54**		PD17	Chiang Mai	
**55**		PD18	Chiang Mai	
**56**		PD19	Chiang Mai	
**57**		PDD1	Nakhon Prathom	
**58**		PDD2	Nakhon Pathom	
**59**		PDD3	Nakhon Pathom	
**60**		PDD4	Nakhon Pathom	
**61**		PDD5	Nakhon Pathom	
**62**		PDD6	Nakhon Pathom	
**63**		PDD7	Nakhon Pathom	
**64**	Pradu Hang Dam	PDD8	Nakhon Pathom	PDH4
**65**		PDD9	Nakhon Pathom	
**66**		PDD10	Nakhon Pathom	
**67**		PDD11	Nakhon Pathom	
**68**		PDD12	Nakhon Pathom	
**69**		PDD13	Nakhon Pathom	
**70**		PDD14	Nakhon Pathom	
**71**		PDD15	Nakhon Pathom	
**72**		PDD16	Nakhon Pathom	
**73**		PDL1	Nonthaburi	PDH5
**74**	Pradu Hang Dam	PDL2	Nonthaburi	
**75**		PDL3	Nonthaburi	
