# Supplementary material for: Samae Dam chicken: a variety of the Pradu Hang Dam breed revealed from microsatellite genotyping data
Source: Anim Biosci. 2024 Jun 25;37(12):2033–43. doi: 10.5713/ab.24.0161 (PMC11541018; doi:10.5713/ab.24.0161)
Supplement: Supplementary file 10 [file ab-24-0161-Supplementary-Table-S2.pdf]

Table S2. Microsatellite primers, primer sequences, and fragment sizes obtained from Pradu Hang Dam and Samae Dam chicken specimens

Primer	Chromosome	Annealing temperate (°C)	Fluorescence	Primer sequence 5′ to 3′		Size (bp)
				Forward	Reverse	
**ADL0268**	1	58	Hex	CTCCACCCCTCTCAGAACTA	CAACTTCCCATCTACCTACT	106–127
**MCW0111**	1	58	Hex	GCTCCATGTGAAGTGGTTTA	GCTCCATGTGAAGTGGTTTA	94–120
**MCW0247**	1	58	Fam	GTTGTTCAAAAGAAGATGCATG	TTGCATTAACTGGGCACTTTC	208–222
**LEI0234**	2	58	Hex	ATGCATCAGATTGGTATTCAA	CGTGGCTGTGAACAAATATG	218–373
**MCW0034**	2	58	Hex	ATCTTGAAACCTCACAAAGC	TCTTCCAACCTATTTTTAGT	211–241
**MCW0206**	2	58	Fam	CTTGACAGTGATGCATTAAATG	ACATCTAGAATTGACTGTTCAC	217–247
**LEI0166**	3	58	Hex	CTCCTGCCCTTAGCTACGCA	TATCCCCTGGCTGGGAGTTT	338–365
**MCW0222**	3	58	Hex	GCAGTTACATTGAAATGATTCC	TTCTCAAAACACCTAGAAGAC	218–230
**MCW0016**	3	58	Fam	ATGGCGCAGAAGGCAAAGCGATAT	TGGCTTCTGAAGCAGTTGCTATGG	126–177
**MCW0037**	3	58	Fam	ACCGGTGCCATCAATTACCTATTA	GAAAGCTCACATGACACTGCGAAA	149–154
**MCW0103**	3	58	Fam	AACTGCGTTGAGAGTGAATGC	TTTCCTAACTGGATGCTTCTG	263–270
**MCW0295**	4	58	Fam	ATCACTACAGAACACCCTCTC	TATGTATGCACGCAGATATCC	82–111
**LEI0094**	4	58	Hex	GATCTCACCAGTATGAGCTGC	TCTCACACTGTAACACAGTGC	243–281
**MCW0078**	5	58	Hex	CCACACGGAGAGGAGAAGGTCT	TAGCATATGAGTGTACTGAGCTTC	144–154
**MCW0098**	4	58	Hex	GGCTGCTTTGTGCTCTTCTCG	CGATGGTCGTAATTCTCACGT	247–257
**MCW0081**	5	58	Fam	GTTGCTGAGAGCCTGGTGCAG	CCTGTATGTGGAATTACTTCTC	107–146
**LEI0192**	6	58	Hex	TGCCAGAGCTTCAGTCTGT	GTCATTACTGTTATGTTTATTGC	251–490
**MCW0014**	6	58	Hex	TATTGGCTCTAGGAACTGTC	GAAATGAAGGTAAGACTAGC	169–202
**MCW0183**	7	58	Hex	ATCCCAGTGTCGAGTATCCGA	TGAGATTTACTGGAGCCTGCC	293–361
**ADL0278**	8	58	Hex	CCAGCAGTCTACCTTCCTAT	TGTCATCCAAGAACAGTGTG	118–136
**MCW0067**	10	58	Fam	GCACTACTGTGTGCTGCAGTTT	GAGATGTAGTTGCCACATTCCGAC	170–182
**ADL0112**	10	58	Hex	GGCTTAAGCTGACCCATTAT	ATCTCAAATGTAATGCGTGC	132–142
**MCW0216**	13	58	Hex	GGGTTTTACAGGATGGGACG	AGTTTCACTCCCAGGGCTCG	135–170
**MCW0104**	13	58	Hex	TAGCACAACTCAAGCTGTGAG	AGACTTGCACAGCTGTGACC	194–249
**MCW0123**	14	58	Hex	CCACTAGAAAAGAACATCCTC	GGCTGATGTAAGAAGGGATGA	84–106
**MCW0330**	17	58	Hex	TGGACCTCATCAGTCTGACAG	AATGTTCTCATAGAGTTCCTGC	259–291
**MCW0165**	23	58	Hex	CAGACATGCATGCCCAGATGA	GATCCAGTCCTGCAGGCTGC	111–115
**MCW0069**	26	58	Hex	GCACTCGAGAAAACTTCCTGCG	ATTGCTTCAGCAAGCATGGGAGGA	155–178

HEX, HEX™ Dye Phosphoramidite; FA, 6-FAM (6-Carboxyfluorescein)
