# Supplementary material for: Samae Dam chicken: a variety of the Pradu Hang Dam breed revealed from microsatellite genotyping data
Source: Anim Biosci. 2024 Jun 25;37(12):2033–43. doi: 10.5713/ab.24.0161 (PMC11541018; doi:10.5713/ab.24.0161)
Supplement: Supplementary file 11 [file ab-24-0161-Supplementary-Table-S3.pdf]

Table S3. Genetic diversity of 75 individuals of Pradu Hang Dam and Samae Dam chicken breeds based on 28 microsatellite loci.

Breeds	Populations	Locus	N	N_a	*AR*	N_{ea}	*I*	H_o	H_e	*F*	*PIC*
		MCW0248	20	5.000	2.921	3.137	1.309	1.000	0.681	–0.468	0.625
		MCW0111	20	3.000	2.682	1.292	0.461	0.250	0.226	–0.105	0.214
		ADL0268	20	3.000	2.000	2.292	0.905	1.000	0.564	–0.774	0.469
		LEI0234	20	11.000	5.524	5.634	1.973	1.000	0.823	–0.216	0.800
		MCW0206	20	7.000	2.921	4.040	1.584	1.000	0.753	–0.329	0.716
		MCW0034	18	9.000	4.556	5.445	1.876	0.944	0.816	–0.157	0.793
		Samae Dam	20	3.000	2.000	2.589	1.017	1.000	0.614	–0.629	0.539
		MCW0103	20	4.000	2.700	2.204	0.890	1.000	0.546	–0.831	0.444
		MCW0016	20	4.000	2.700	2.749	1.121	1.000	0.636	–0.572	0.573
		LEI0166	20	3.000	2.921	2.658	1.037	1.000	0.624	–0.603	0.553
		MCW0037	20	2.000	1.921	1.471	0.500	0.400	0.320	–0.250	0.269
		MCW0295	20	5.000	3.842	3.125	1.305	1.000	0.680	–0.471	0.627
		LEI0094	20	4.000	2.921	3.404	1.306	1.000	0.706	–0.416	0.657
		MCW0098	20	3.000	2.000	2.524	1.004	0.950	0.604	–0.573	0.532
	Department of livestock Uthai Thani province	MCW0078	20	3.000	2.700	2.100	0.792	1.000	0.524	–0.909	0.410
		MCW0081	20	3.000	2.000	2.667	1.040	1.000	0.625	–0.600	0.555
		LEI0192	20	8.000	4.542	3.333	1.569	1.000	0.700	–0.429	0.673
		MCW0014	20	6.000	2.000	4.494	1.621	1.000	0.778	–0.286	0.744
		MCW0183	20	3.000	2.000	2.589	1.017	1.000	0.614	–0.629	0.539
		ADL0278	18	4.000	2.875	2.445	1.020	1.000	0.591	–0.692	0.509
		MCW0067	20	5.000	4.763	3.433	1.357	1.000	0.709	–0.411	0.658
		ADL0112	15	4.000	3.000	2.473	1.073	0.533	0.596	0.104	0.539
		MCW0216	20	9.000	6.563	3.292	1.580	1.000	0.696	–0.436	0.668
		MCW0104	17	13.000	6.000	4.281	1.973	0.941	0.766	–0.228	0.750
		MCW0123	18	2.000	2.000	2.000	0.693	1.000	0.500	–1.000	0.375
		MCW0330	18	6.000	2.992	2.830	1.290	1.000	0.647	–0.547	0.593
		MCW0165	20	3.000	2.000	2.204	0.857	1.000	0.546	–0.831	0.442
		MCW0069	18	4.000	2.992	3.130	1.237	1.000	0.681	–0.469	0.622
		Mean	20	4.964	3.144	2.994	1.193	0.929	0.627	–0.491	0.567

Breeds	Populations	Locus	N	N_a	*AR*	N_{ea}	*I*	H_o	H_e	*F*	*PIC*
		SD	0	2.769	1.301	1.032	0.399	0.193	0.131	0.256	0.145
	Sanhawat Farm Uthai Thani	MCW0248	4	2.000	1.929	1.882	0.662	0.750	0.469	–0.600	0.359
		MCW0111	4	3.000	2.557	2.667	1.040	1.000	0.625	–0.600	0.555
		ADL0268	4	3.000	2.643	2.909	1.082	0.750	0.656	–0.143	0.582
		LEI0234	4	4.000	2.857	3.200	1.255	0.750	0.688	–0.091	0.630
		MCW0206	4	4.000	3.000	3.556	1.321	0.250	0.719	0.652	0.667
		MCW0034	4	1.000	1.000	1.000	0.000	0.000	0.000	#N/A	0.000
		MCW0222	4	1.000	1.000	1.000	0.000	0.000	0.000	#N/A	0.000
		MCW0103	2	2.000	2.000	1.600	0.562	1.000	0.375	–1.667	0.305
		MCW0016	4	3.000	2.557	2.667	1.040	0.000	0.625	1.000	0.555
		LEI0166	3	3.000	2.800	3.000	1.099	0.667	0.667	0.000	0.593
		MCW0037	3	1.000	1.000	1.000	0.000	0.000	0.000	#N/A	0.000
		MCW0295	4	2.000	1.500	1.280	0.377	0.250	0.219	–0.143	0.195
		LEI0094	4	3.000	2.643	2.909	1.082	1.000	0.656	–0.524	0.582
		MCW0098	4	2.000	1.786	1.600	0.562	0.500	0.375	–0.333	0.305
		MCW0078	3	1.000	1.000	1.000	0.000	0.000	0.000	#N/A	0.000
		MCW0081	4	2.000	1.786	1.600	0.562	0.500	0.375	–0.333	0.305
		LEI0192	4	2.000	1.971	2.000	0.693	0.500	0.500	0.000	0.375
		MCW0014	4	2.000	1.786	1.600	0.562	0.000	0.375	1.000	0.305
		MCW0183	4	2.000	1.500	1.280	0.377	0.250	0.219	–0.143	0.195
		ADL0278	4	1.000	1.000	1.000	0.000	0.000	0.000	#N/A	0.000
		MCW0067	4	2.000	1.786	1.600	0.562	0.500	0.375	–0.333	0.305
		ADL0112	4	4.000	2.857	3.200	1.255	0.500	0.688	0.273	0.630
		MCW0216	4	1.000	1.000	1.000	0.000	0.000	0.000	#N/A	0.000
		MCW0104	4	3.000	2.000	1.684	0.736	0.250	0.406	0.385	0.371
		MCW0123	4	3.000	2.286	2.133	0.900	0.500	0.531	0.059	0.468
		MCW0330	4	2.000	1.500	1.280	0.377	0.250	0.219	–0.143	0.195
		MCW0165	4	2.000	1.929	1.882	0.662	0.750	0.469	–0.600	0.359
		MCW0069	4	2.000	1.786	1.600	0.562	0.500	0.375	–0.333	0.305
		Mean	4	2.250	1.909	1.897	0.619	0.408	0.379	–0.119	0.326
		SD	0	0.928	0.650	0.798	0.427	0.339	0.248	0.578	0.222

Breeds	Populations	Locus	N	N_a	*AR*	N_{ea}	*I*	H_o	H_e	*F*	*PIC*
	Phitsanulok 1	MCW0248	7	3.000	3.000	2.882	1.079	0.857	0.653	–0.313	0.580
		MCW0111	10	5.000	4.397	3.226	1.327	0.700	0.690	–0.014	0.640
		ADL0268	10	3.000	2.697	1.653	0.687	0.300	0.395	0.241	0.347
		LEI0234	8	6.000	5.858	5.120	1.700	0.750	0.805	0.068	0.776
		MCW0206	9	5.000	4.551	3.240	1.336	0.556	0.691	0.196	0.642
		MCW0034	10	3.000	2.700	1.802	0.746	0.400	0.445	0.101	0.381
		Pradu Hang Dam	9	3.000	2.995	2.656	1.026	0.444	0.623	0.287	0.544
		MCW0103	10	2.000	2.000	1.923	0.673	0.400	0.480	0.167	0.365
		MCW0016	9	4.000	3.739	2.746	1.132	0.333	0.636	0.476	0.565
		LEI0166	8	4.000	3.875	3.122	1.223	0.500	0.680	0.264	0.618
		MCW0037	10	4.000	3.665	2.128	0.999	0.700	0.530	–0.321	0.489
		MCW0295	9	6.000	5.438	2.842	1.380	0.444	0.648	0.314	0.620
		LEI0094	8	3.000	2.992	1.910	0.831	0.375	0.477	0.213	0.427
		MCW0098	9	2.000	2.000	1.528	0.530	0.222	0.346	0.357	0.286
		MCW0078	9	2.000	2.000	1.906	0.668	0.111	0.475	0.766	0.362
		MCW0081	10	3.000	2.982	2.247	0.938	0.700	0.555	–0.261	0.491
		LEI0192	9	6.000	5.111	2.700	1.303	0.444	0.630	0.294	0.590
		MCW0014	7	2.000	2.000	1.324	0.410	0.000	0.245	1.000	0.215
		MCW0183	9	4.000	3.739	2.656	1.117	0.667	0.623	–0.069	0.557
		ADL0278	9	4.000	3.551	1.800	0.855	0.556	0.444	–0.250	0.409
		MCW0067	7	4.000	4.000	2.178	1.029	0.571	0.541	–0.057	0.502
		ADL0112	9	3.000	2.778	2.219	0.868	0.556	0.549	–0.011	0.448
		MCW0216	8	3.000	3.000	2.909	1.082	0.375	0.656	0.429	0.582
		MCW0104	8	5.000	4.625	2.560	1.190	0.750	0.609	–0.231	0.561
		MCW0123	10	5.000	4.821	3.509	1.426	0.500	0.715	0.301	0.677
		MCW0330	10	6.000	5.318	4.255	1.574	0.900	0.765	–0.176	0.728
		MCW0165	9	2.000	2.000	1.528	0.530	0.444	0.346	–0.286	0.286
		MCW0069	10	5.000	4.100	2.500	1.139	0.700	0.600	–0.167	0.540
		Mean	10	3.821	3.569	2.538	1.029	0.509	0.566	0.119	0.508
		SD	0	1.335	1.142	0.847	0.324	0.213	0.135	0.326	0.140

Breeds	Populations	Locus	N	N_a	*AR*	N_{ea}	*I*	H_o	H_e	*F*	*PIC*
	Phitsanulok 2	MCW0248	3	3.000	2.600	2.571	1.011	0.667	0.611	–0.091	0.535
		MCW0111	3	1.000	1.000	1.000	0.000	0.000	0.000	#N/A	0.000
		ADL0268	3	4.000	3.000	3.000	1.242	0.667	0.667	0.000	0.620
		LEI0234	3	3.000	2.800	3.000	1.099	0.000	0.667	1.000	0.593
		MCW0206	3	3.000	2.800	3.000	1.099	0.000	0.667	1.000	0.593
		MCW0034	3	1.000	1.000	1.000	0.000	0.000	0.000	#N/A	0.000
		MCW0222	2	2.000	2.000	1.600	0.562	0.500	0.375	–0.333	0.305
		MCW0103	3	2.000	2.000	2.000	0.693	1.000	0.500	–1.000	0.375
		MCW0016	3	5.000	3.600	4.500	1.561	0.667	0.778	0.143	0.744
		LEI0166	3	3.000	2.800	3.000	1.099	0.000	0.667	1.000	0.593
		MCW0037	3	3.000	2.333	2.000	0.868	0.667	0.500	–0.333	0.449
		MCW0295	3	3.000	2.600	2.571	1.011	0.333	0.611	0.455	0.535
		LEI0094	3	5.000	3.600	4.500	1.561	0.667	0.778	0.143	0.744
		MCW0098	2	3.000	3.000	2.667	1.040	0.500	0.625	0.200	0.555
		MCW0078	3	2.000	2.000	2.000	0.693	1.000	0.500	–1.000	0.375
		MCW0081	3	5.000	3.600	4.500	1.561	1.000	0.778	–0.286	0.744
		LEI0192	3	2.000	1.933	1.800	0.637	0.000	0.444	1.000	0.346
		MCW0014	3	2.000	1.933	1.800	0.637	0.000	0.444	1.000	0.346
		MCW0183	3	4.000	3.000	3.000	1.242	1.000	0.667	–0.500	0.620
		ADL0278	3	3.000	2.800	3.000	1.099	0.000	0.667	1.000	0.593
		MCW0067	3	2.000	1.933	1.800	0.637	0.000	0.444	1.000	0.346
		ADL0112	3	2.000	1.667	1.385	0.451	0.333	0.278	–0.200	0.239
		MCW0216	3	5.000	3.600	4.500	1.561	1.000	0.778	–0.286	0.744
		MCW0104	3	2.000	1.933	1.800	0.637	0.000	0.444	1.000	0.346
		MCW0123	3	2.000	1.933	1.800	0.637	0.667	0.444	–0.500	0.346
		MCW0330	3	4.000	3.200	3.600	1.330	0.667	0.722	0.077	0.671
		MCW0165	3	2.000	1.933	1.800	0.637	0.000	0.444	1.000	0.346
		MCW0069	3	5.000	3.600	4.500	1.561	0.667	0.778	0.143	0.744
		Mean	3	2.964	2.507	2.632	0.934	0.429	0.546	0.217	0.480
		SD	0	1.232	0.759	1.097	0.439	0.391	0.208	0.664	0.208
	Chiang Mai	MCW0248	19	4.000	3.840	2.188	0.959	0.632	0.543	–0.163	0.475

Breeds	Populations	Locus	N	N_a	*AR*	N_{ea}	*I*	H_o	H_e	*F*	*PIC*
		MCW0111	19	3.000	2.842	2.069	0.788	0.895	0.517	–0.732	0.408
		ADL0268	19	8.000	7.797	4.813	1.788	1.000	0.792	–0.262	0.766
		LEI0234	19	16.000	14.924	10.776	2.561	0.842	0.907	0.072	0.900
		MCW0206	19	7.000	6.820	4.079	1.647	0.947	0.755	–0.255	0.727
		MCW0034	16	6.000	6.000	2.709	1.314	0.625	0.631	0.009	0.596
		MCW0222	19	4.000	3.840	2.329	0.991	0.789	0.571	–0.383	0.493
		MCW0103	19	4.000	4.000	3.505	1.312	0.947	0.715	–0.326	0.662
		MCW0016	19	6.000	5.821	4.323	1.569	1.000	0.769	–0.301	0.732
		LEI0166	18	6.000	5.768	3.176	1.357	1.000	0.685	–0.459	0.634
		MCW0037	19	3.000	3.000	2.854	1.072	0.947	0.650	–0.458	0.575
		MCW0295	19	7.000	6.936	4.719	1.714	0.947	0.788	–0.202	0.759
		LEI0094	19	11.000	10.438	5.232	2.004	0.632	0.809	0.219	0.792
		MCW0098	19	4.000	4.000	3.327	1.280	0.789	0.699	–0.129	0.645
		MCW0078	18	7.000	6.888	5.838	1.824	0.778	0.829	0.061	0.805
		MCW0081	19	4.000	3.842	2.588	1.084	0.789	0.614	–0.287	0.549
		LEI0192	19	12.000	11.010	6.171	2.089	0.947	0.838	–0.131	0.820
		MCW0014	19	7.000	6.658	3.471	1.483	0.895	0.712	–0.257	0.666
		MCW0183	18	4.000	3.879	1.503	0.678	0.389	0.335	–0.161	0.314
		ADL0278	19	5.000	4.837	2.820	1.218	0.474	0.645	0.266	0.584
		MCW0067	18	7.000	6.998	5.492	1.817	0.333	0.818	0.592	0.794
		ADL0112	19	4.000	3.821	2.195	0.934	0.737	0.544	–0.354	0.463
		MCW0216	19	7.000	6.661	3.574	1.526	0.842	0.720	–0.169	0.685
		MCW0104	18	5.000	4.981	3.256	1.330	0.167	0.693	0.759	0.639
		MCW0123	18	6.000	5.889	4.765	1.639	0.611	0.790	0.227	0.758
		MCW0330	17	5.000	4.939	2.627	1.203	0.706	0.619	–0.140	0.574
		MCW0165	19	4.000	3.842	2.947	1.158	1.000	0.661	–0.514	0.595
		MCW0069	18	9.000	8.878	6.680	2.035	0.500	0.850	0.412	0.834
		Mean	19	6.250	6.041	3.930	1.442	0.756	0.696	–0.109	0.652
		SD	0	2.927	2.712	1.902	0.437	0.224	0.124	0.338	0.141
	Nakhon Pathom	MCW0248	15	3.000	2.999	2.381	0.943	0.600	0.580	–0.034	0.492
		MCW0111	16	5.000	4.795	3.261	1.326	0.750	0.693	–0.082	0.639

Breeds	Populations	Locus	N	N_a	*AR*	N_{ea}	*I*	H_o	H_e	*F*	*PIC*
		ADL0268	15	4.000	3.856	2.432	1.056	0.600	0.589	–0.019	0.528
		LEI0234	16	7.000	5.756	3.737	1.556	0.813	0.732	–0.109	0.694
		MCW0206	16	4.000	3.930	1.962	0.943	0.313	0.490	0.363	0.454
		MCW0034	15	9.000	8.807	7.258	2.073	0.800	0.862	0.072	0.847
		MCW0222	16	2.000	2.000	1.519	0.525	0.438	0.342	–0.280	0.283
		MCW0103	16	2.000	2.000	1.969	0.685	0.375	0.492	0.238	0.371
		MCW0016	16	7.000	5.595	3.631	1.531	0.875	0.725	–0.208	0.688
		LEI0166	15	3.000	3.000	2.761	1.056	0.867	0.638	–0.359	0.565
		MCW0037	15	3.000	2.999	1.867	0.817	0.533	0.464	–0.148	0.419
		MCW0295	15	5.000	4.984	3.982	1.479	0.667	0.749	0.110	0.709
		LEI0094	15	4.000	3.857	2.332	1.040	0.800	0.571	–0.401	0.519
		MCW0098	15	2.000	1.984	1.142	0.245	0.133	0.124	–0.071	0.117
		MCW0078	16	3.000	2.999	1.910	0.831	0.563	0.477	–0.180	0.427
		MCW0081	16	3.000	3.000	2.768	1.059	0.625	0.639	0.021	0.567
		LEI0192	14	6.000	4.997	4.404	1.622	0.357	0.773	0.538	0.741
		MCW0014	16	4.000	3.931	1.822	0.874	0.313	0.451	0.307	0.417
		MCW0183	16	5.000	4.566	2.560	1.111	0.625	0.609	–0.026	0.532
		ADL0278	15	5.000	4.968	3.261	1.361	0.600	0.693	0.135	0.650
		MCW0067	16	5.000	4.595	2.844	1.226	0.813	0.648	–0.253	0.587
		ADL0112	14	4.000	3.997	3.294	1.260	0.571	0.696	0.179	0.639
		MCW0216	15	6.000	5.714	4.592	1.615	0.933	0.782	–0.193	0.748
		MCW0104	15	4.000	3.984	2.761	1.157	0.667	0.638	–0.045	0.578
		MCW0123	15	8.000	7.838	6.818	1.981	0.933	0.853	–0.094	0.836
		MCW0330	13	4.000	4.000	3.159	1.261	0.769	0.683	–0.126	0.633
		MCW0165	15	3.000	3.000	2.632	1.030	0.933	0.620	–0.505	0.548
		MCW0069	15	5.000	4.856	2.866	1.285	0.733	0.651	–0.126	0.612
		Mean	16	4.464	4.250	3.069	1.177	0.643	0.617	–0.046	0.566
		SD	0	1.774	1.584	1.395	0.402	0.211	0.156	0.233	0.161
	Nonthaburi	MCW0248	3	2.000	1.667	1.385	0.451	0.333	0.278	–0.200	0.239
		MCW0111	3	2.000	1.667	1.385	0.451	0.333	0.278	–0.200	0.239
		ADL0268	3	3.000	2.800	3.000	1.099	0.667	0.667	0.000	0.593

Breeds	Populations	Locus	N	N_a	*AR*	N_{ea}	*I*	H_o	H_e	*F*	*PIC*
		LEI0234	3	5.000	3.600	4.500	1.561	1.000	0.778	–0.286	0.744
		MCW0206	3	4.000	3.200	3.600	1.330	1.000	0.722	–0.385	0.671
		MCW0034	3	3.000	2.600	2.571	1.011	0.333	0.611	0.455	0.535
		MCW0222	3	2.000	1.933	1.800	0.637	0.000	0.444	1.000	0.346
		MCW0103	3	2.000	1.933	1.800	0.637	0.667	0.444	–0.500	0.346
		MCW0016	3	4.000	3.200	3.600	1.330	0.667	0.722	0.077	0.671
		LEI0166	3	2.000	1.933	1.800	0.637	0.667	0.444	–0.500	0.346
		MCW0037	3	3.000	2.800	3.000	1.099	0.000	0.667	1.000	0.593
		MCW0295	3	4.000	3.000	3.000	1.242	1.000	0.667	–0.500	0.620
		LEI0094	3	2.000	1.933	1.800	0.637	0.667	0.444	–0.500	0.346
		MCW0098	3	2.000	1.667	1.385	0.451	0.333	0.278	–0.200	0.239
		MCW0078	3	2.000	1.933	1.800	0.637	0.000	0.444	1.000	0.346
		MCW0081	3	3.000	2.600	2.571	1.011	0.333	0.611	0.455	0.535
		LEI0192	3	4.000	3.000	3.000	1.242	1.000	0.667	–0.500	0.620
		MCW0014	3	3.000	2.600	2.571	1.011	1.000	0.611	–0.636	0.535
		MCW0183	3	3.000	2.600	2.571	1.011	1.000	0.611	–0.636	0.535
		ADL0278	3	3.000	2.333	2.000	0.868	0.333	0.500	0.333	0.449
		MCW0067	2	4.000	4.000	4.000	1.386	1.000	0.750	–0.333	0.703
		ADL0112	3	2.000	1.933	1.800	0.637	0.667	0.444	–0.500	0.346
		MCW0216	3	2.000	1.667	1.385	0.451	0.333	0.278	–0.200	0.239
		MCW0104	3	5.000	3.600	4.500	1.561	1.000	0.778	–0.286	0.744
		MCW0123	3	4.000	3.000	3.000	1.242	0.667	0.667	0.000	0.620
		MCW0330	3	3.000	2.600	2.571	1.011	1.000	0.611	–0.636	0.535
		MCW0165	3	2.000	1.933	1.800	0.637	0.667	0.444	–0.500	0.346
		MCW0069	3	2.000	1.933	1.800	0.637	0.667	0.444	–0.500	0.346
		Mean	3	2.929	2.488	2.500	0.925	0.619	0.547	–0.131	0.480
		SD	0	0.979	0.667	0.925	0.351	0.336	0.158	0.503	0.165

N, number of samples; N_a, number of alleles; *AR*, allelic richness; N_{ea}, number of effective alleles; *I*, Shannon's information index; H_o, observed heterozygosity; H_e, expected heterozygosity; *F*, fixation index; *PIC,* polymorphic information content
