# Supplementary material for: Samae Dam chicken: a variety of the Pradu Hang Dam breed revealed from microsatellite genotyping data
Source: Anim Biosci. 2024 Jun 25;37(12):2033–43. doi: 10.5713/ab.24.0161 (PMC11541018; doi:10.5713/ab.24.0161)
Supplement: Supplementary file 12 [file ab-24-0161-Supplementary-Table-S4.pdf]

Table S4. Pairwise differentiation of linkage disequilibrium of Pradu Hang Dam chickens derived from Phitsanulok 1 population (PDH1) based on 28 microsatellite loci

Locus 1	Locus 2	*p*-value
**MCW0248**	MCW0111	1.000
**MCW0248**	ADL0268	1.000
**MCW0111**	ADL0268	0.555
**MCW0248**	LEI0234	N/A
**MCW0111**	LEI0234	N/A
**ADL0268**	LEI0234	N/A
**MCW0248**	MCW0206	1.000
**MCW0111**	MCW0206	1.000
**ADL0268**	MCW0206	1.000
**LEI0234**	MCW0206	N/A
**MCW0248**	MCW0034	0.498
**MCW0111**	MCW0034	0.412
**ADL0268**	MCW0034	0.183
**LEI0234**	MCW0034	N/A
**MCW0206**	MCW0034	0.444
**MCW0248**	MCW0222	1.000
**MCW0111**	MCW0222	0.443
**ADL0268**	MCW0222	1.000
**LEI0234**	MCW0222	N/A
**MCW0206**	MCW0222	1.000
**MCW0034**	MCW0222	0.305
**MCW0248**	MCW0103	0.540
**MCW0111**	MCW0103	0.856
**ADL0268**	MCW0103	0.031
**LEI0234**	MCW0103	N/A
**MCW0206**	MCW0103	1.000
**MCW0034**	MCW0103	0.087
**MCW0222**	MCW0103	0.633
**MCW0248**	MCW0016	0.337
**MCW0111**	MCW0016	0.123
**ADL0268**	MCW0016	0.924
**LEI0234**	MCW0016	N/A
**MCW0206**	MCW0016	1.000
**MCW0034**	MCW0016	0.135
**MCW0222**	MCW0016	1.000
**MCW0103**	MCW0016	0.841
**MCW0248**	LEI0166	1.000
**MCW0111**	LEI0166	1.000
**ADL0268**	LEI0166	0.855
**LEI0234**	LEI0166	N/A
**MCW0206**	LEI0166	0.125

**Locus 1**	**Locus 2**	***p*-value**
**MCW0034**	LEI0166	0.636
**MCW0222**	LEI0166	0.447
**MCW0103**	LEI0166	0.565
**MCW0016**	LEI0166	1.000
**MCW0248**	MCW0037	0.146
**MCW0111**	MCW0037	0.701
**ADL0268**	MCW0037	1.000
**LEI0234**	MCW0037	N/A
**MCW0206**	MCW0037	0.350
**MCW0034**	MCW0037	1.000
**MCW0222**	MCW0037	1.000
**MCW0103**	MCW0037	0.485
**MCW0016**	MCW0037	0.215
**LEI0166**	MCW0037	1.000
**MCW0248**	MCW0295	1.000
**MCW0111**	MCW0295	1.000
**ADL0268**	MCW0295	0.119
**LEI0234**	MCW0295	N/A
**MCW0206**	MCW0295	1.000
**MCW0034**	MCW0295	0.219
**MCW0222**	MCW0295	0.853
**MCW0103**	MCW0295	0.127
**MCW0016**	MCW0295	1.000
**LEI0166**	MCW0295	0.533
**MCW0037**	MCW0295	0.876
**MCW0248**	LEI0094	0.764
**MCW0111**	LEI0094	1.000
**ADL0268**	LEI0094	0.148
**LEI0234**	LEI0094	N/A
**MCW0206**	LEI0094	0.253
**MCW0034**	LEI0094	0.237
**MCW0222**	LEI0094	1.000
**MCW0103**	LEI0094	0.489
**MCW0016**	LEI0094	0.549
**LEI0166**	LEI0094	0.623
**MCW0037**	LEI0094	0.856
**MCW0295**	LEI0094	0.541
**MCW0248**	MCW0098	0.766
**MCW0111**	MCW0098	0.911
**ADL0268**	MCW0098	1.000
**LEI0234**	MCW0098	N/A
**MCW0206**	MCW0098	0.721
**MCW0034**	MCW0098	0.764

**Locus 1**	**Locus 2**	***p*-value**
**MCW0222**	MCW0098	1.000
**MCW0103**	MCW0098	0.253
**MCW0016**	MCW0098	1.000
**LEI0166**	MCW0098	0.284
**MCW0037**	MCW0098	0.378
**MCW0295**	MCW0098	1.000
**LEI0094**	MCW0098	0.857
**MCW0248**	MCW0078	1.000
**MCW0111**	MCW0078	0.353
**ADL0268**	MCW0078	1.000
**LEI0234**	MCW0078	N/A
**MCW0206**	MCW0078	0.548
**MCW0034**	MCW0078	0.406
**MCW0222**	MCW0078	0.920
**MCW0103**	MCW0078	0.761
**MCW0016**	MCW0078	0.130
**LEI0166**	MCW0078	0.475
**MCW0037**	MCW0078	1.000
**MCW0295**	MCW0078	0.293
**LEI0094**	MCW0078	1.000
**MCW0098**	MCW0078	0.536
**MCW0248**	MCW0081	1.000
**MCW0111**	MCW0081	0.262
**ADL0268**	MCW0081	0.489
**LEI0234**	MCW0081	N/A
**MCW0206**	MCW0081	1.000
**MCW0034**	MCW0081	0.915
**MCW0222**	MCW0081	0.543
**MCW0103**	MCW0081	0.915
**MCW0016**	MCW0081	0.697
**LEI0166**	MCW0081	0.508
**MCW0037**	MCW0081	0.646
**MCW0295**	MCW0081	0.882
**LEI0094**	MCW0081	0.909
**MCW0098**	MCW0081	1.000
**MCW0078**	MCW0081	0.617
**MCW0248**	LEI0192	1.000
**MCW0111**	LEI0192	0.473
**ADL0268**	LEI0192	0.570
**LEI0234**	LEI0192	N/A
**MCW0206**	LEI0192	1.000
**MCW0034**	LEI0192	0.905
**MCW0222**	LEI0192	1.000

**Locus 1**	**Locus 2**	***p*-value**
**MCW0103**	LEI0192	0.215
**MCW0016**	LEI0192	0.313
**LEI0166**	LEI0192	0.606
**MCW0037**	LEI0192	1.000
**MCW0295**	LEI0192	0.232
**LEI0094**	LEI0192	0.886
**MCW0098**	LEI0192	0.593
**MCW0078**	LEI0192	0.097
**MCW0081**	LEI0192	0.863
**MCW0248**	MCW0014	N/A
**MCW0111**	MCW0014	0.721
**ADL0268**	MCW0014	1.000
**LEI0234**	MCW0014	N/A
**MCW0206**	MCW0014	N/A
**MCW0034**	MCW0014	1.000
**MCW0222**	MCW0014	0.569
**MCW0103**	MCW0014	0.572
**MCW0016**	MCW0014	0.494
**LEI0166**	MCW0014	0.664
**MCW0037**	MCW0014	0.286
**MCW0295**	MCW0014	1.000
**LEI0094**	MCW0014	1.000
**MCW0098**	MCW0014	1.000
**MCW0078**	MCW0014	N/A
**MCW0081**	MCW0014	0.418
**LEI0192**	MCW0014	1.000
**MCW0248**	MCW0183	1.000
**MCW0111**	MCW0183	0.325
**ADL0268**	MCW0183	0.828
**LEI0234**	MCW0183	N/A
**MCW0206**	MCW0183	1.000
**MCW0034**	MCW0183	0.124
**MCW0222**	MCW0183	0.636
**MCW0103**	MCW0183	0.794
**MCW0016**	MCW0183	0.409
**LEI0166**	MCW0183	1.000
**MCW0037**	MCW0183	0.620
**MCW0295**	MCW0183	0.750
**LEI0094**	MCW0183	1.000
**MCW0098**	MCW0183	1.000
**MCW0078**	MCW0183	0.543
**MCW0081**	MCW0183	1.000
**LEI0192**	MCW0183	0.423

**Locus 1**	**Locus 2**	***p*-value**
**MCW0014**	MCW0183	1.000
**MCW0248**	ADL0278	0.601
**MCW0111**	ADL0278	0.579
**ADL0268**	ADL0278	0.047
**LEI0234**	ADL0278	N/A
**MCW0206**	ADL0278	1.000
**MCW0034**	ADL0278	0.327
**MCW0222**	ADL0278	0.529
**MCW0103**	ADL0278	0.194
**MCW0016**	ADL0278	0.680
**LEI0166**	ADL0278	0.689
**MCW0037**	ADL0278	0.941
**MCW0295**	ADL0278	0.259
**LEI0094**	ADL0278	0.159
**MCW0098**	ADL0278	1.000
**MCW0078**	ADL0278	0.617
**MCW0081**	ADL0278	0.139
**LEI0192**	ADL0278	0.313
**MCW0014**	ADL0278	0.586
**MCW0183**	ADL0278	1.000
**MCW0248**	MCW0067	1.000
**MCW0111**	MCW0067	1.000
**ADL0268**	MCW0067	0.119
**LEI0234**	MCW0067	N/A
**MCW0206**	MCW0067	N/A
**MCW0034**	MCW0067	0.518
**MCW0222**	MCW0067	1.000
**MCW0103**	MCW0067	0.661
**MCW0016**	MCW0067	1.000
**LEI0166**	MCW0067	0.274
**MCW0037**	MCW0067	1.000
**MCW0295**	MCW0067	0.208
**LEI0094**	MCW0067	0.501
**MCW0098**	MCW0067	0.851
**MCW0078**	MCW0067	1.000
**MCW0081**	MCW0067	0.525
**LEI0192**	MCW0067	0.630
**MCW0014**	MCW0067	1.000
**MCW0183**	MCW0067	0.293
**ADL0278**	MCW0067	0.741
**MCW0248**	ADL0112	0.546
**MCW0111**	ADL0112	1.000
**ADL0268**	ADL0112	0.112

Locus 1	Locus 2	*p*-value
**LEI0234**	ADL0112	N/A
**MCW0206**	ADL0112	1.000
**MCW0034**	ADL0112	0.639
**MCW0222**	ADL0112	1.000
**MCW0103**	ADL0112	0.020
**MCW0016**	ADL0112	1.000
**LEI0166**	ADL0112	0.669
**MCW0037**	ADL0112	0.107
**MCW0295**	ADL0112	0.014
**LEI0094**	ADL0112	0.602
**MCW0098**	ADL0112	1.000
**MCW0078**	ADL0112	0.482
**MCW0081**	ADL0112	0.446
**LEI0192**	ADL0112	0.179
**MCW0014**	ADL0112	1.000
**MCW0183**	ADL0112	0.504
**ADL0278**	ADL0112	0.195
**MCW0067**	ADL0112	0.111
**MCW0248**	MCW0216	1.000
**MCW0111**	MCW0216	1.000
**ADL0268**	MCW0216	0.639
**LEI0234**	MCW0216	N/A
**MCW0206**	MCW0216	1.000
**MCW0034**	MCW0216	1.000
**MCW0222**	MCW0216	1.000
**MCW0103**	MCW0216	1.000
**MCW0016**	MCW0216	1.000
**LEI0166**	MCW0216	1.000
**MCW0037**	MCW0216	1.000
**MCW0295**	MCW0216	1.000
**LEI0094**	MCW0216	0.505
**MCW0098**	MCW0216	1.000
**MCW0078**	MCW0216	1.000
**MCW0081**	MCW0216	0.033
**LEI0192**	MCW0216	1.000
**MCW0014**	MCW0216	N/A
**MCW0183**	MCW0216	1.000
**ADL0278**	MCW0216	0.485
**MCW0067**	MCW0216	1.000
**ADL0112**	MCW0216	1.000
**MCW0248**	MCW0104	1.000
**MCW0111**	MCW0104	0.589
**ADL0268**	MCW0104	0.031

Locus 1	Locus 2	*p*-value
**LEI0234**	MCW0104	N/A
**MCW0206**	MCW0104	1.000
**MCW0034**	MCW0104	0.929
**MCW0222**	MCW0104	0.626
**MCW0103**	MCW0104	0.293
**MCW0016**	MCW0104	1.000
**LEI0166**	MCW0104	0.397
**MCW0037**	MCW0104	1.000
**MCW0295**	MCW0104	0.508
**LEI0094**	MCW0104	0.789
**MCW0098**	MCW0104	1.000
**MCW0078**	MCW0104	1.000
**MCW0081**	MCW0104	0.026
**LEI0192**	MCW0104	0.374
**MCW0014**	MCW0104	0.605
**MCW0183**	MCW0104	1.000
**ADL0278**	MCW0104	0.030
**MCW0067**	MCW0104	0.315
**ADL0112**	MCW0104	0.502
**MCW0216**	MCW0104	0.065
**MCW0248**	MCW0123	1.000
**MCW0111**	MCW0123	1.000
**ADL0268**	MCW0123	0.862
**LEI0234**	MCW0123	N/A
**MCW0206**	MCW0123	1.000
**MCW0034**	MCW0123	1.000
**MCW0222**	MCW0123	0.644
**MCW0103**	MCW0123	0.795
**MCW0016**	MCW0123	1.000
**LEI0166**	MCW0123	0.362
**MCW0037**	MCW0123	0.634
**MCW0295**	MCW0123	1.000
**LEI0094**	MCW0123	0.735
**MCW0098**	MCW0123	0.238
**MCW0078**	MCW0123	0.907
**MCW0081**	MCW0123	0.161
**LEI0192**	MCW0123	1.000
**MCW0014**	MCW0123	0.559
**MCW0183**	MCW0123	1.000
**ADL0278**	MCW0123	0.184
**MCW0067**	MCW0123	1.000
**ADL0112**	MCW0123	0.665
**MCW0216**	MCW0123	0.278

Locus 1	Locus 2	*p*-value
**MCW0104**	MCW0123	0.273
**MCW0248**	MCW0330	1.000
**MCW0111**	MCW0330	0.620
**ADL0268**	MCW0330	0.496
**LEI0234**	MCW0330	N/A
**MCW0206**	MCW0330	0.373
**MCW0034**	MCW0330	0.339
**MCW0222**	MCW0330	0.216
**MCW0103**	MCW0330	1.000
**MCW0016**	MCW0330	1.000
**LEI0166**	MCW0330	1.000
**MCW0037**	MCW0330	0.892
**MCW0295**	MCW0330	0.602
**LEI0094**	MCW0330	0.891
**MCW0098**	MCW0330	0.346
**MCW0078**	MCW0330	0.903
**MCW0081**	MCW0330	0.916
**LEI0192**	MCW0330	1.000
**MCW0014**	MCW0330	1.000
**MCW0183**	MCW0330	0.530
**ADL0278**	MCW0330	0.358
**MCW0067**	MCW0330	0.374
**ADL0112**	MCW0330	0.910
**MCW0216**	MCW0330	1.000
**MCW0104**	MCW0330	0.494
**MCW0123**	MCW0330	1.000
**MCW0248**	MCW0165	0.771
**MCW0111**	MCW0165	0.433
**ADL0268**	MCW0165	0.524
**LEI0234**	MCW0165	N/A
**MCW0206**	MCW0165	1.000
**MCW0034**	MCW0165	0.568
**MCW0222**	MCW0165	0.281
**MCW0103**	MCW0165	0.129
**MCW0016**	MCW0165	0.853
**LEI0166**	MCW0165	0.853
**MCW0037**	MCW0165	1.000
**MCW0295**	MCW0165	0.581
**LEI0094**	MCW0165	0.791
**MCW0098**	MCW0165	0.444
**MCW0078**	MCW0165	0.488
**MCW0081**	MCW0165	0.049
**LEI0192**	MCW0165	0.523

Locus 1	Locus 2	*p*-value
**MCW0014**	MCW0165	1.000
**MCW0183**	MCW0165	0.714
**ADL0278**	MCW0165	0.679
**MCW0067**	MCW0165	0.143
**ADL0112**	MCW0165	0.142
**MCW0216**	MCW0165	0.201
**MCW0104**	MCW0165	0.279
**MCW0123**	MCW0165	0.524
**MCW0330**	MCW0165	0.710
**MCW0248**	MCW0069	1.000
**MCW0111**	MCW0069	0.124
**ADL0268**	MCW0069	0.981
**LEI0234**	MCW0069	N/A
**MCW0206**	MCW0069	0.218
**MCW0034**	MCW0069	0.332
**MCW0222**	MCW0069	0.628
**MCW0103**	MCW0069	1.000
**MCW0016**	MCW0069	0.017
**LEI0166**	MCW0069	0.223
**MCW0037**	MCW0069	0.898
**MCW0295**	MCW0069	0.859
**LEI0094**	MCW0069	0.454
**MCW0098**	MCW0069	0.516
**MCW0078**	MCW0069	0.014
**MCW0081**	MCW0069	0.914
**LEI0192**	MCW0069	0.565
**MCW0014**	MCW0069	0.718
**MCW0183**	MCW0069	1.000
**ADL0278**	MCW0069	1.000
**MCW0067**	MCW0069	1.000
**ADL0112**	MCW0069	1.000
**MCW0216**	MCW0069	1.000
**MCW0104**	MCW0069	1.000
**MCW0123**	MCW0069	1.000
**MCW0330**	MCW0069	1.000
**MCW0165**	MCW0069	0.516
