# Supplementary material for: Samae Dam chicken: a variety of the Pradu Hang Dam breed revealed from microsatellite genotyping data
Source: Anim Biosci. 2024 Jun 25;37(12):2033–43. doi: 10.5713/ab.24.0161 (PMC11541018; doi:10.5713/ab.24.0161)
Supplement: Supplementary file 14 [file ab-24-0161-Supplementary-Table-S6.pdf]

Table S6. Pairwise differentiation of linkage disequilibrium of Pradu Hang Dam chickens derived from Chiang Mai population (PDH3) based on 28 microsatellite loci

Locus 1	Locus 2	*p*-value
**MCW0248**	MCW0111	0.175
**MCW0248**	ADL0268	0.737
**MCW0111**	ADL0268	0.133
**MCW0248**	LEI0234	N/A
**MCW0111**	LEI0234	N/A
**ADL0268**	LEI0234	N/A
**MCW0248**	MCW0206	0.695
**MCW0111**	MCW0206	0.064
**ADL0268**	MCW0206	0.717
**LEI0234**	MCW0206	N/A
**MCW0248**	MCW0034	0.930
**MCW0111**	MCW0034	0.723
**ADL0268**	MCW0034	0.526
**LEI0234**	MCW0034	N/A
**MCW0206**	MCW0034	0.647
**MCW0248**	MCW0222	0.731
**MCW0111**	MCW0222	1.000
**ADL0268**	MCW0222	0.679
**LEI0234**	MCW0222	N/A
**MCW0206**	MCW0222	0.493
**MCW0034**	MCW0222	0.934
**MCW0248**	MCW0103	0.223
**MCW0111**	MCW0103	0.316
**ADL0268**	MCW0103	0.012
**LEI0234**	MCW0103	N/A
**MCW0206**	MCW0103	0.191
**MCW0034**	MCW0103	0.592
**MCW0222**	MCW0103	0.236
**MCW0248**	MCW0016	0.004
**MCW0111**	MCW0016	0.269
**ADL0268**	MCW0016	0.093
**LEI0234**	MCW0016	N/A
**MCW0206**	MCW0016	0.626
**MCW0034**	MCW0016	1.000
**MCW0222**	MCW0016	0.767
**MCW0103**	MCW0016	0.109
**MCW0248**	LEI0166	0.225
**MCW0111**	LEI0166	0.588
**ADL0268**	LEI0166	0.937
**LEI0234**	LEI0166	N/A
**MCW0206**	LEI0166	0.160

**Locus 1**	**Locus 2**	***p*-value**
**MCW0034**	LEI0166	0.170
**MCW0222**	LEI0166	0.252
**MCW0103**	LEI0166	0.588
**MCW0016**	LEI0166	0.774
**MCW0248**	MCW0037	0.064
**MCW0111**	MCW0037	0.075
**ADL0268**	MCW0037	0.368
**LEI0234**	MCW0037	N/A
**MCW0206**	MCW0037	0.185
**MCW0034**	MCW0037	0.471
**MCW0222**	MCW0037	0.706
**MCW0103**	MCW0037	0.016
**MCW0016**	MCW0037	0.034
**LEI0166**	MCW0037	0.870
**MCW0248**	MCW0295	0.580
**MCW0111**	MCW0295	0.884
**ADL0268**	MCW0295	0.405
**LEI0234**	MCW0295	N/A
**MCW0206**	MCW0295	1.000
**MCW0034**	MCW0295	0.248
**MCW0222**	MCW0295	0.804
**MCW0103**	MCW0295	0.975
**MCW0016**	MCW0295	0.872
**LEI0166**	MCW0295	0.512
**MCW0037**	MCW0295	0.994
**MCW0248**	LEI0094	0.642
**MCW0111**	LEI0094	0.862
**ADL0268**	LEI0094	0.555
**LEI0234**	LEI0094	N/A
**MCW0206**	LEI0094	0.706
**MCW0034**	LEI0094	0.702
**MCW0222**	LEI0094	0.035
**MCW0103**	LEI0094	0.878
**MCW0016**	LEI0094	0.383
**LEI0166**	LEI0094	1.000
**MCW0037**	LEI0094	0.956
**MCW0295**	LEI0094	0.625
**MCW0248**	MCW0098	0.421
**MCW0111**	MCW0098	0.179
**ADL0268**	MCW0098	0.585
**LEI0234**	MCW0098	N/A
**MCW0206**	MCW0098	0.597
**MCW0034**	MCW0098	0.737

**Locus 1**	**Locus 2**	***p*-value**
**MCW0222**	MCW0098	0.001
**MCW0103**	MCW0098	0.511
**MCW0016**	MCW0098	0.446
**LEI0166**	MCW0098	0.432
**MCW0037**	MCW0098	0.336
**MCW0295**	MCW0098	0.620
**LEI0094**	MCW0098	0.062
**MCW0248**	MCW0078	0.166
**MCW0111**	MCW0078	0.335
**ADL0268**	MCW0078	0.378
**LEI0234**	MCW0078	N/A
**MCW0206**	MCW0078	0.700
**MCW0034**	MCW0078	1.000
**MCW0222**	MCW0078	0.027
**MCW0103**	MCW0078	0.147
**MCW0016**	MCW0078	0.577
**LEI0166**	MCW0078	0.705
**MCW0037**	MCW0078	0.888
**MCW0295**	MCW0078	0.809
**LEI0094**	MCW0078	0.028
**MCW0098**	MCW0078	0.059
**MCW0248**	MCW0081	0.900
**MCW0111**	MCW0081	1.000
**ADL0268**	MCW0081	0.146
**LEI0234**	MCW0081	N/A
**MCW0206**	MCW0081	0.887
**MCW0034**	MCW0081	0.570
**MCW0222**	MCW0081	0.093
**MCW0103**	MCW0081	0.977
**MCW0016**	MCW0081	0.774
**LEI0166**	MCW0081	0.310
**MCW0037**	MCW0081	0.880
**MCW0295**	MCW0081	0.529
**LEI0094**	MCW0081	0.014
**MCW0098**	MCW0081	0.118
**MCW0078**	MCW0081	0.136
**MCW0248**	LEI0192	0.796
**MCW0111**	LEI0192	0.493
**ADL0268**	LEI0192	0.007
**LEI0234**	LEI0192	N/A
**MCW0206**	LEI0192	0.619
**MCW0034**	LEI0192	0.213
**MCW0222**	LEI0192	0.860

Locus 1	Locus 2	*p*-value
**MCW0103**	LEI0192	0.043
**MCW0016**	LEI0192	0.009
**LEI0166**	LEI0192	0.431
**MCW0037**	LEI0192	0.735
**MCW0295**	LEI0192	0.546
**LEI0094**	LEI0192	1.000
**MCW0098**	LEI0192	0.907
**MCW0078**	LEI0192	0.118
**MCW0081**	LEI0192	0.945
**MCW0248**	MCW0014	1.000
**MCW0111**	MCW0014	0.370
**ADL0268**	MCW0014	0.401
**LEI0234**	MCW0014	N/A
**MCW0206**	MCW0014	0.767
**MCW0034**	MCW0014	0.928
**MCW0222**	MCW0014	0.699
**MCW0103**	MCW0014	0.295
**MCW0016**	MCW0014	0.319
**LEI0166**	MCW0014	0.860
**MCW0037**	MCW0014	0.262
**MCW0295**	MCW0014	0.252
**LEI0094**	MCW0014	0.431
**MCW0098**	MCW0014	0.146
**MCW0078**	MCW0014	0.622
**MCW0081**	MCW0014	0.109
**LEI0192**	MCW0014	0.735
**MCW0248**	MCW0183	0.793
**MCW0111**	MCW0183	0.473
**ADL0268**	MCW0183	0.554
**LEI0234**	MCW0183	N/A
**MCW0206**	MCW0183	0.512
**MCW0034**	MCW0183	0.291
**MCW0222**	MCW0183	0.204
**MCW0103**	MCW0183	0.964
**MCW0016**	MCW0183	0.808
**LEI0166**	MCW0183	0.748
**MCW0037**	MCW0183	0.705
**MCW0295**	MCW0183	0.486
**LEI0094**	MCW0183	0.161
**MCW0098**	MCW0183	0.973
**MCW0078**	MCW0183	0.545
**MCW0081**	MCW0183	0.523
**LEI0192**	MCW0183	0.162

**Locus 1**	**Locus 2**	***p*-value**
**MCW0014**	MCW0183	0.692
**MCW0248**	ADL0278	0.328
**MCW0111**	ADL0278	0.497
**ADL0268**	ADL0278	0.156
**LEI0234**	ADL0278	N/A
**MCW0206**	ADL0278	0.486
**MCW0034**	ADL0278	0.203
**MCW0222**	ADL0278	0.905
**MCW0103**	ADL0278	0.454
**MCW0016**	ADL0278	0.905
**LEI0166**	ADL0278	0.143
**MCW0037**	ADL0278	0.944
**MCW0295**	ADL0278	0.349
**LEI0094**	ADL0278	0.572
**MCW0098**	ADL0278	0.697
**MCW0078**	ADL0278	0.052
**MCW0081**	ADL0278	0.996
**LEI0192**	ADL0278	0.329
**MCW0014**	ADL0278	0.913
**MCW0183**	ADL0278	0.136
**MCW0248**	MCW0067	0.016
**MCW0111**	MCW0067	0.749
**ADL0268**	MCW0067	0.926
**LEI0234**	MCW0067	N/A
**MCW0206**	MCW0067	0.241
**MCW0034**	MCW0067	1.000
**MCW0222**	MCW0067	0.102
**MCW0103**	MCW0067	0.204
**MCW0016**	MCW0067	0.194
**LEI0166**	MCW0067	0.700
**MCW0037**	MCW0067	0.045
**MCW0295**	MCW0067	1.000
**LEI0094**	MCW0067	0.259
**MCW0098**	MCW0067	0.024
**MCW0078**	MCW0067	0.352
**MCW0081**	MCW0067	0.466
**LEI0192**	MCW0067	1.000
**MCW0014**	MCW0067	0.563
**MCW0183**	MCW0067	0.654
**ADL0278**	MCW0067	0.974
**MCW0248**	ADL0112	0.063
**MCW0111**	ADL0112	0.152
**ADL0268**	ADL0112	0.290

**Locus 1**	**Locus 2**	***p*-value**
**LEI0234**	ADL0112	N/A
**MCW0206**	ADL0112	0.103
**MCW0034**	ADL0112	0.113
**MCW0222**	ADL0112	0.955
**MCW0103**	ADL0112	0.556
**MCW0016**	ADL0112	0.039
**LEI0166**	ADL0112	0.281
**MCW0037**	ADL0112	0.180
**MCW0295**	ADL0112	0.899
**LEI0094**	ADL0112	0.736
**MCW0098**	ADL0112	0.801
**MCW0078**	ADL0112	0.998
**MCW0081**	ADL0112	0.166
**LEI0192**	ADL0112	0.610
**MCW0014**	ADL0112	0.921
**MCW0183**	ADL0112	0.972
**ADL0278**	ADL0112	0.846
**MCW0067**	ADL0112	0.636
**MCW0248**	MCW0216	0.117
**MCW0111**	MCW0216	0.969
**ADL0268**	MCW0216	0.378
**LEI0234**	MCW0216	N/A
**MCW0206**	MCW0216	0.919
**MCW0034**	MCW0216	0.802
**MCW0222**	MCW0216	0.750
**MCW0103**	MCW0216	0.158
**MCW0016**	MCW0216	0.065
**LEI0166**	MCW0216	0.978
**MCW0037**	MCW0216	0.353
**MCW0295**	MCW0216	0.072
**LEI0094**	MCW0216	0.840
**MCW0098**	MCW0216	0.890
**MCW0078**	MCW0216	0.575
**MCW0081**	MCW0216	0.786
**LEI0192**	MCW0216	0.647
**MCW0014**	MCW0216	0.742
**MCW0183**	MCW0216	0.857
**ADL0278**	MCW0216	0.901
**MCW0067**	MCW0216	0.897
**ADL0112**	MCW0216	0.400
**MCW0248**	MCW0104	0.717
**MCW0111**	MCW0104	0.037
**ADL0268**	MCW0104	0.871

**Locus 1**	**Locus 2**	***p*-value**
**LEI0234**	MCW0104	N/A
**MCW0206**	MCW0104	0.078
**MCW0034**	MCW0104	0.926
**MCW0222**	MCW0104	0.816
**MCW0103**	MCW0104	0.974
**MCW0016**	MCW0104	0.645
**LEI0166**	MCW0104	0.784
**MCW0037**	MCW0104	0.560
**MCW0295**	MCW0104	0.722
**LEI0094**	MCW0104	0.502
**MCW0098**	MCW0104	0.074
**MCW0078**	MCW0104	0.604
**MCW0081**	MCW0104	0.859
**LEI0192**	MCW0104	1.000
**MCW0014**	MCW0104	0.453
**MCW0183**	MCW0104	0.156
**ADL0278**	MCW0104	0.310
**MCW0067**	MCW0104	0.971
**ADL0112**	MCW0104	0.458
**MCW0216**	MCW0104	0.673
**MCW0248**	MCW0123	0.272
**MCW0111**	MCW0123	1.000
**ADL0268**	MCW0123	0.433
**LEI0234**	MCW0123	N/A
**MCW0206**	MCW0123	0.654
**MCW0034**	MCW0123	0.784
**MCW0222**	MCW0123	0.824
**MCW0103**	MCW0123	0.682
**MCW0016**	MCW0123	0.761
**LEI0166**	MCW0123	0.759
**MCW0037**	MCW0123	0.788
**MCW0295**	MCW0123	0.851
**LEI0094**	MCW0123	0.724
**MCW0098**	MCW0123	0.781
**MCW0078**	MCW0123	0.397
**MCW0081**	MCW0123	0.080
**LEI0192**	MCW0123	1.000
**MCW0014**	MCW0123	0.999
**MCW0183**	MCW0123	0.127
**ADL0278**	MCW0123	0.535
**MCW0067**	MCW0123	0.552
**ADL0112**	MCW0123	0.268
**MCW0216**	MCW0123	0.182

Locus 1	Locus 2	*p*-value
**MCW0104**	MCW0123	0.178
**MCW0248**	MCW0330	0.726
**MCW0111**	MCW0330	1.000
**ADL0268**	MCW0330	0.698
**LEI0234**	MCW0330	N/A
**MCW0206**	MCW0330	0.953
**MCW0034**	MCW0330	0.534
**MCW0222**	MCW0330	0.957
**MCW0103**	MCW0330	0.608
**MCW0016**	MCW0330	0.979
**LEI0166**	MCW0330	0.172
**MCW0037**	MCW0330	0.731
**MCW0295**	MCW0330	0.075
**LEI0094**	MCW0330	0.950
**MCW0098**	MCW0330	0.820
**MCW0078**	MCW0330	0.669
**MCW0081**	MCW0330	0.144
**LEI0192**	MCW0330	0.788
**MCW0014**	MCW0330	0.039
**MCW0183**	MCW0330	0.727
**ADL0278**	MCW0330	0.060
**MCW0067**	MCW0330	0.531
**ADL0112**	MCW0330	1.000
**MCW0216**	MCW0330	0.347
**MCW0104**	MCW0330	0.869
**MCW0123**	MCW0330	0.928
**MCW0248**	MCW0165	0.635
**MCW0111**	MCW0165	1.000
**ADL0268**	MCW0165	0.396
**LEI0234**	MCW0165	N/A
**MCW0206**	MCW0165	0.823
**MCW0034**	MCW0165	0.979
**MCW0222**	MCW0165	0.537
**MCW0103**	MCW0165	0.626
**MCW0016**	MCW0165	0.505
**LEI0166**	MCW0165	0.723
**MCW0037**	MCW0165	0.188
**MCW0295**	MCW0165	0.552
**LEI0094**	MCW0165	0.251
**MCW0098**	MCW0165	0.904
**MCW0078**	MCW0165	0.480
**MCW0081**	MCW0165	0.328
**LEI0192**	MCW0165	0.454

Locus 1	Locus 2	*p*-value
**MCW0014**	MCW0165	0.243
**MCW0183**	MCW0165	0.045
**ADL0278**	MCW0165	0.889
**MCW0067**	MCW0165	0.645
**ADL0112**	MCW0165	0.805
**MCW0216**	MCW0165	0.260
**MCW0104**	MCW0165	0.176
**MCW0123**	MCW0165	0.090
**MCW0330**	MCW0165	0.438
**MCW0248**	MCW0069	0.183
**MCW0111**	MCW0069	0.407
**ADL0268**	MCW0069	0.725
**LEI0234**	MCW0069	N/A
**MCW0206**	MCW0069	1.000
**MCW0034**	MCW0069	0.401
**MCW0222**	MCW0069	0.601
**MCW0103**	MCW0069	0.159
**MCW0016**	MCW0069	1.000
**LEI0166**	MCW0069	0.824
**MCW0037**	MCW0069	0.980
**MCW0295**	MCW0069	0.588
**LEI0094**	MCW0069	1.000
**MCW0098**	MCW0069	0.577
**MCW0078**	MCW0069	0.515
**MCW0081**	MCW0069	0.728
**LEI0192**	MCW0069	1.000
**MCW0014**	MCW0069	0.741
**MCW0183**	MCW0069	0.942
**ADL0278**	MCW0069	0.776
**MCW0067**	MCW0069	0.166
**ADL0112**	MCW0069	0.879
**MCW0216**	MCW0069	1.000
**MCW0104**	MCW0069	0.873
**MCW0123**	MCW0069	0.597
**MCW0330**	MCW0069	0.609
**MCW0165**	MCW0069	0.803
