# Supplementary material for: Samae Dam chicken: a variety of the Pradu Hang Dam breed revealed from microsatellite genotyping data
Source: Anim Biosci. 2024 Jun 25;37(12):2033–43. doi: 10.5713/ab.24.0161 (PMC11541018; doi:10.5713/ab.24.0161)
Supplement: Supplementary file 15 [file ab-24-0161-Supplementary-Table-S7.pdf]

Table S7. Pairwise differentiation of linkage disequilibrium of Pradu Hang Dam chickens derived from Nakhon Pathom population (PDH4) based on 28 microsatellite loci

Locus 1	Locus 2	*p*-value
**MCW0248**	MCW0111	0.127
**MCW0248**	ADL0268	0.056
**MCW0111**	ADL0268	0.692
**MCW0248**	LEI0234	0.148
**MCW0111**	LEI0234	0.826
**ADL0268**	LEI0234	0.239
**MCW0248**	MCW0206	0.274
**MCW0111**	MCW0206	0.966
**ADL0268**	MCW0206	0.023
**LEI0234**	MCW0206	0.230
**MCW0248**	MCW0034	1.000
**MCW0111**	MCW0034	0.457
**ADL0268**	MCW0034	0.671
**LEI0234**	MCW0034	1.000
**MCW0206**	MCW0034	0.537
**MCW0248**	MCW0222	0.444
**MCW0111**	MCW0222	0.515
**ADL0268**	MCW0222	0.378
**LEI0234**	MCW0222	0.492
**MCW0206**	MCW0222	0.266
**MCW0034**	MCW0222	0.153
**MCW0248**	MCW0103	0.694
**MCW0111**	MCW0103	0.063
**ADL0268**	MCW0103	0.151
**LEI0234**	MCW0103	0.357
**MCW0206**	MCW0103	0.155
**MCW0034**	MCW0103	0.864
**MCW0222**	MCW0103	1.000
**MCW0248**	MCW0016	0.273
**MCW0111**	MCW0016	0.945
**ADL0268**	MCW0016	0.031
**LEI0234**	MCW0016	0.050
**MCW0206**	MCW0016	0.268
**MCW0034**	MCW0016	0.551
**MCW0222**	MCW0016	0.161
**MCW0103**	MCW0016	0.178
**MCW0248**	LEI0166	0.225
**MCW0111**	LEI0166	0.671
**ADL0268**	LEI0166	0.031
**LEI0234**	LEI0166	0.071
**MCW0206**	LEI0166	0.873

**Locus 1**	**Locus 2**	***p*-value**
**MCW0034**	LEI0166	0.865
**MCW0222**	LEI0166	0.813
**MCW0103**	LEI0166	0.160
**MCW0016**	LEI0166	0.464
**MCW0248**	MCW0037	0.073
**MCW0111**	MCW0037	0.111
**ADL0268**	MCW0037	0.814
**LEI0234**	MCW0037	0.245
**MCW0206**	MCW0037	0.236
**MCW0034**	MCW0037	1.000
**MCW0222**	MCW0037	0.384
**MCW0103**	MCW0037	0.565
**MCW0016**	MCW0037	0.371
**LEI0166**	MCW0037	0.155
**MCW0248**	MCW0295	0.837
**MCW0111**	MCW0295	0.716
**ADL0268**	MCW0295	0.864
**LEI0234**	MCW0295	0.633
**MCW0206**	MCW0295	0.133
**MCW0034**	MCW0295	0.382
**MCW0222**	MCW0295	0.471
**MCW0103**	MCW0295	0.476
**MCW0016**	MCW0295	1.000
**LEI0166**	MCW0295	0.990
**MCW0037**	MCW0295	0.193
**MCW0248**	LEI0094	0.747
**MCW0111**	LEI0094	0.176
**ADL0268**	LEI0094	0.757
**LEI0234**	LEI0094	0.057
**MCW0206**	LEI0094	0.878
**MCW0034**	LEI0094	0.846
**MCW0222**	LEI0094	0.388
**MCW0103**	LEI0094	0.874
**MCW0016**	LEI0094	0.806
**LEI0166**	LEI0094	0.489
**MCW0037**	LEI0094	0.606
**MCW0295**	LEI0094	0.396
**MCW0248**	MCW0098	0.602
**MCW0111**	MCW0098	0.661
**ADL0268**	MCW0098	0.635
**LEI0234**	MCW0098	0.434
**MCW0206**	MCW0098	0.149
**MCW0034**	MCW0098	0.642

**Locus 1**	**Locus 2**	***p*-value**
**MCW0222**	MCW0098	1.000
**MCW0103**	MCW0098	0.655
**MCW0016**	MCW0098	0.087
**LEI0166**	MCW0098	0.559
**MCW0037**	MCW0098	1.000
**MCW0295**	MCW0098	0.360
**LEI0094**	MCW0098	0.283
**MCW0248**	MCW0078	0.231
**MCW0111**	MCW0078	0.621
**ADL0268**	MCW0078	0.749
**LEI0234**	MCW0078	0.323
**MCW0206**	MCW0078	0.136
**MCW0034**	MCW0078	1.000
**MCW0222**	MCW0078	1.000
**MCW0103**	MCW0078	0.046
**MCW0016**	MCW0078	0.885
**LEI0166**	MCW0078	0.031
**MCW0037**	MCW0078	0.020
**MCW0295**	MCW0078	0.777
**LEI0094**	MCW0078	0.844
**MCW0098**	MCW0078	1.000
**MCW0248**	MCW0081	0.312
**MCW0111**	MCW0081	0.320
**ADL0268**	MCW0081	0.621
**LEI0234**	MCW0081	0.527
**MCW0206**	MCW0081	0.172
**MCW0034**	MCW0081	1.000
**MCW0222**	MCW0081	0.870
**MCW0103**	MCW0081	0.927
**MCW0016**	MCW0081	0.777
**LEI0166**	MCW0081	1.000
**MCW0037**	MCW0081	0.962
**MCW0295**	MCW0081	0.852
**LEI0094**	MCW0081	0.232
**MCW0098**	MCW0081	0.225
**MCW0078**	MCW0081	0.707
**MCW0248**	LEI0192	0.628
**MCW0111**	LEI0192	0.662
**ADL0268**	LEI0192	0.298
**LEI0234**	LEI0192	0.290
**MCW0206**	LEI0192	0.300
**MCW0034**	LEI0192	1.000
**MCW0222**	LEI0192	0.005

**Locus 1**	**Locus 2**	***p*-value**
**MCW0103**	LEI0192	0.836
**MCW0016**	LEI0192	0.788
**LEI0166**	LEI0192	0.729
**MCW0037**	LEI0192	0.950
**MCW0295**	LEI0192	1.000
**LEI0094**	LEI0192	0.085
**MCW0098**	LEI0192	1.000
**MCW0078**	LEI0192	0.036
**MCW0081**	LEI0192	0.854
**MCW0248**	MCW0014	0.448
**MCW0111**	MCW0014	0.018
**ADL0268**	MCW0014	0.702
**LEI0234**	MCW0014	0.440
**MCW0206**	MCW0014	0.290
**MCW0034**	MCW0014	0.945
**MCW0222**	MCW0014	1.000
**MCW0103**	MCW0014	0.732
**MCW0016**	MCW0014	0.174
**LEI0166**	MCW0014	0.403
**MCW0037**	MCW0014	0.258
**MCW0295**	MCW0014	0.053
**LEI0094**	MCW0014	0.032
**MCW0098**	MCW0014	0.397
**MCW0078**	MCW0014	0.130
**MCW0081**	MCW0014	0.184
**LEI0192**	MCW0014	0.994
**MCW0248**	MCW0183	0.305
**MCW0111**	MCW0183	0.067
**ADL0268**	MCW0183	0.159
**LEI0234**	MCW0183	0.304
**MCW0206**	MCW0183	0.412
**MCW0034**	MCW0183	0.713
**MCW0222**	MCW0183	0.357
**MCW0103**	MCW0183	0.437
**MCW0016**	MCW0183	0.289
**LEI0166**	MCW0183	0.068
**MCW0037**	MCW0183	0.117
**MCW0295**	MCW0183	0.932
**LEI0094**	MCW0183	0.916
**MCW0098**	MCW0183	0.153
**MCW0078**	MCW0183	0.255
**MCW0081**	MCW0183	0.755
**LEI0192**	MCW0183	0.575

**Locus 1**	**Locus 2**	***p*-value**
**MCW0014**	MCW0183	0.200
**MCW0248**	ADL0278	0.789
**MCW0111**	ADL0278	0.123
**ADL0268**	ADL0278	0.681
**LEI0234**	ADL0278	0.721
**MCW0206**	ADL0278	0.974
**MCW0034**	ADL0278	1.000
**MCW0222**	ADL0278	0.698
**MCW0103**	ADL0278	0.273
**MCW0016**	ADL0278	0.069
**LEI0166**	ADL0278	0.462
**MCW0037**	ADL0278	0.036
**MCW0295**	ADL0278	1.000
**LEI0094**	ADL0278	0.462
**MCW0098**	ADL0278	0.577
**MCW0078**	ADL0278	0.548
**MCW0081**	ADL0278	0.520
**LEI0192**	ADL0278	1.000
**MCW0014**	ADL0278	0.054
**MCW0183**	ADL0278	0.150
**MCW0248**	MCW0067	0.988
**MCW0111**	MCW0067	0.307
**ADL0268**	MCW0067	0.025
**LEI0234**	MCW0067	0.187
**MCW0206**	MCW0067	0.133
**MCW0034**	MCW0067	0.791
**MCW0222**	MCW0067	0.360
**MCW0103**	MCW0067	0.884
**MCW0016**	MCW0067	0.001
**LEI0166**	MCW0067	0.593
**MCW0037**	MCW0067	0.712
**MCW0295**	MCW0067	0.935
**LEI0094**	MCW0067	0.656
**MCW0098**	MCW0067	0.304
**MCW0078**	MCW0067	0.461
**MCW0081**	MCW0067	0.489
**LEI0192**	MCW0067	0.483
**MCW0014**	MCW0067	0.029
**MCW0183**	MCW0067	0.180
**ADL0278**	MCW0067	0.187
**MCW0248**	ADL0112	0.204
**MCW0111**	ADL0112	0.354
**ADL0268**	ADL0112	0.869

Locus 1	Locus 2	*p*-value
**LEI0234**	ADL0112	0.016
**MCW0206**	ADL0112	0.396
**MCW0034**	ADL0112	1.000
**MCW0222**	ADL0112	0.736
**MCW0103**	ADL0112	0.220
**MCW0016**	ADL0112	0.814
**LEI0166**	ADL0112	0.411
**MCW0037**	ADL0112	0.088
**MCW0295**	ADL0112	1.000
**LEI0094**	ADL0112	0.518
**MCW0098**	ADL0112	0.416
**MCW0078**	ADL0112	0.043
**MCW0081**	ADL0112	0.573
**LEI0192**	ADL0112	0.781
**MCW0014**	ADL0112	0.686
**MCW0183**	ADL0112	0.157
**ADL0278**	ADL0112	0.273
**MCW0067**	ADL0112	0.514
**MCW0248**	MCW0216	0.874
**MCW0111**	MCW0216	0.698
**ADL0268**	MCW0216	0.816
**LEI0234**	MCW0216	1.000
**MCW0206**	MCW0216	0.490
**MCW0034**	MCW0216	1.000
**MCW0222**	MCW0216	0.011
**MCW0103**	MCW0216	0.755
**MCW0016**	MCW0216	0.745
**LEI0166**	MCW0216	0.734
**MCW0037**	MCW0216	0.332
**MCW0295**	MCW0216	1.000
**LEI0094**	MCW0216	0.132
**MCW0098**	MCW0216	1.000
**MCW0078**	MCW0216	0.289
**MCW0081**	MCW0216	1.000
**LEI0192**	MCW0216	0.021
**MCW0014**	MCW0216	0.904
**MCW0183**	MCW0216	0.896
**ADL0278**	MCW0216	0.524
**MCW0067**	MCW0216	0.938
**ADL0112**	MCW0216	0.579
**MCW0248**	MCW0104	0.979
**MCW0111**	MCW0104	0.937
**ADL0268**	MCW0104	0.132

**Locus 1**	**Locus 2**	***p*-value**
**LEI0234**	MCW0104	0.932
**MCW0206**	MCW0104	0.062
**MCW0034**	MCW0104	0.690
**MCW0222**	MCW0104	0.049
**MCW0103**	MCW0104	0.618
**MCW0016**	MCW0104	0.454
**LEI0166**	MCW0104	0.352
**MCW0037**	MCW0104	0.643
**MCW0295**	MCW0104	0.113
**LEI0094**	MCW0104	0.671
**MCW0098**	MCW0104	0.255
**MCW0078**	MCW0104	0.869
**MCW0081**	MCW0104	0.723
**LEI0192**	MCW0104	0.270
**MCW0014**	MCW0104	0.466
**MCW0183**	MCW0104	0.217
**ADL0278**	MCW0104	0.238
**MCW0067**	MCW0104	0.292
**ADL0112**	MCW0104	1.000
**MCW0216**	MCW0104	0.413
**MCW0248**	MCW0123	0.224
**MCW0111**	MCW0123	0.667
**ADL0268**	MCW0123	0.630
**LEI0234**	MCW0123	0.507
**MCW0206**	MCW0123	0.961
**MCW0034**	MCW0123	1.000
**MCW0222**	MCW0123	0.153
**MCW0103**	MCW0123	0.498
**MCW0016**	MCW0123	0.102
**LEI0166**	MCW0123	1.000
**MCW0037**	MCW0123	0.790
**MCW0295**	MCW0123	0.330
**LEI0094**	MCW0123	0.713
**MCW0098**	MCW0123	0.617
**MCW0078**	MCW0123	0.832
**MCW0081**	MCW0123	0.572
**LEI0192**	MCW0123	1.000
**MCW0014**	MCW0123	0.216
**MCW0183**	MCW0123	0.727
**ADL0278**	MCW0123	0.002
**MCW0067**	MCW0123	0.228
**ADL0112**	MCW0123	1.000
**MCW0216**	MCW0123	1.000

**Locus 1**	**Locus 2**	***p*-value**
**MCW0104**	MCW0123	0.240
**MCW0248**	MCW0330	0.702
**MCW0111**	MCW0330	1.000
**ADL0268**	MCW0330	0.453
**LEI0234**	MCW0330	0.067
**MCW0206**	MCW0330	0.064
**MCW0034**	MCW0330	0.596
**MCW0222**	MCW0330	0.785
**MCW0103**	MCW0330	0.274
**MCW0016**	MCW0330	0.074
**LEI0166**	MCW0330	0.069
**MCW0037**	MCW0330	0.811
**MCW0295**	MCW0330	1.000
**LEI0094**	MCW0330	0.960
**MCW0098**	MCW0330	0.035
**MCW0078**	MCW0330	0.614
**MCW0081**	MCW0330	1.000
**LEI0192**	MCW0330	1.000
**MCW0014**	MCW0330	0.598
**MCW0183**	MCW0330	0.136
**ADL0278**	MCW0330	0.761
**MCW0067**	MCW0330	0.169
**ADL0112**	MCW0330	0.393
**MCW0216**	MCW0330	0.552
**MCW0104**	MCW0330	0.968
**MCW0123**	MCW0330	0.409
**MCW0248**	MCW0165	0.257
**MCW0111**	MCW0165	0.430
**ADL0268**	MCW0165	0.351
**LEI0234**	MCW0165	0.012
**MCW0206**	MCW0165	0.012
**MCW0034**	MCW0165	1.000
**MCW0222**	MCW0165	1.000
**MCW0103**	MCW0165	0.562
**MCW0016**	MCW0165	0.061
**LEI0166**	MCW0165	0.077
**MCW0037**	MCW0165	0.017
**MCW0295**	MCW0165	0.201
**LEI0094**	MCW0165	0.054
**MCW0098**	MCW0165	1.000
**MCW0078**	MCW0165	0.018
**MCW0081**	MCW0165	0.981
**LEI0192**	MCW0165	0.350

**Locus 1**	**Locus 2**	***p*-value**
**MCW0014**	MCW0165	0.314
**MCW0183**	MCW0165	0.572
**ADL0278**	MCW0165	0.912
**MCW0067**	MCW0165	0.083
**ADL0112**	MCW0165	0.006
**MCW0216**	MCW0165	0.146
**MCW0104**	MCW0165	1.000
**MCW0123**	MCW0165	0.909
**MCW0330**	MCW0165	0.076
**MCW0248**	MCW0069	0.814
**MCW0111**	MCW0069	0.307
**ADL0268**	MCW0069	0.039
**LEI0234**	MCW0069	0.387
**MCW0206**	MCW0069	0.057
**MCW0034**	MCW0069	0.513
**MCW0222**	MCW0069	0.810
**MCW0103**	MCW0069	0.014
**MCW0016**	MCW0069	0.080
**LEI0166**	MCW0069	0.531
**MCW0037**	MCW0069	0.172
**MCW0295**	MCW0069	0.371
**LEI0094**	MCW0069	0.750
**MCW0098**	MCW0069	0.163
**MCW0078**	MCW0069	0.984
**MCW0081**	MCW0069	0.702
**LEI0192**	MCW0069	1.000
**MCW0014**	MCW0069	0.163
**MCW0183**	MCW0069	0.029
**ADL0278**	MCW0069	0.009
**MCW0067**	MCW0069	0.385
**ADL0112**	MCW0069	0.822
**MCW0216**	MCW0069	1.000
**MCW0104**	MCW0069	0.278
**MCW0123**	MCW0069	0.124
**MCW0330**	MCW0069	0.070
**MCW0165**	MCW0069	0.937
