# Supplementary material for: Samae Dam chicken: a variety of the Pradu Hang Dam breed revealed from microsatellite genotyping data
Source: Anim Biosci. 2024 Jun 25;37(12):2033–43. doi: 10.5713/ab.24.0161 (PMC11541018; doi:10.5713/ab.24.0161)
Supplement: Supplementary file 16 [file ab-24-0161-Supplementary-Table-S8.pdf]

Table S8. Pairwise differentiation of linkage disequilibrium of Pradu Hang Dam chickens derived from Nonthaburi population (PDH5) based on 28 microsatellite loci

Locus 1	Locus 2	*p*-value
**MCW0248**	MCW0111	0.337
**MCW0248**	ADL0268	1.000
**MCW0111**	ADL0268	1.000
**MCW0248**	LEI0234	N/A
**MCW0111**	LEI0234	N/A
**ADL0268**	LEI0234	N/A
**MCW0248**	MCW0206	N/A
**MCW0111**	MCW0206	N/A
**ADL0268**	MCW0206	N/A
**LEI0234**	MCW0206	N/A
**MCW0248**	MCW0034	N/A
**MCW0111**	MCW0034	N/A
**ADL0268**	MCW0034	N/A
**LEI0234**	MCW0034	N/A
**MCW0206**	MCW0034	N/A
**MCW0248**	MCW0222	0.334
**MCW0111**	MCW0222	0.334
**ADL0268**	MCW0222	1.000
**LEI0234**	MCW0222	N/A
**MCW0206**	MCW0222	N/A
**MCW0034**	MCW0222	N/A
**MCW0248**	MCW0103	1.000
**MCW0111**	MCW0103	1.000
**ADL0268**	MCW0103	0.329
**LEI0234**	MCW0103	N/A
**MCW0206**	MCW0103	N/A
**MCW0034**	MCW0103	N/A
**MCW0222**	MCW0103	1.000
**MCW0248**	MCW0016	N/A
**MCW0111**	MCW0016	N/A
**ADL0268**	MCW0016	N/A
**LEI0234**	MCW0016	N/A
**MCW0206**	MCW0016	N/A
**MCW0034**	MCW0016	N/A
**MCW0222**	MCW0016	N/A
**MCW0103**	MCW0016	N/A
**MCW0248**	LEI0166	0.335
**MCW0111**	LEI0166	0.333
**ADL0268**	LEI0166	1.000
**LEI0234**	LEI0166	N/A
**MCW0206**	LEI0166	N/A

**Locus 1**	**Locus 2**	***p*-value**
**MCW0034**	LEI0166	N/A
**MCW0222**	LEI0166	0.335
**MCW0103**	LEI0166	1.000
**MCW0016**	LEI0166	N/A
**MCW0248**	MCW0037	N/A
**MCW0111**	MCW0037	N/A
**ADL0268**	MCW0037	N/A
**LEI0234**	MCW0037	N/A
**MCW0206**	MCW0037	N/A
**MCW0034**	MCW0037	N/A
**MCW0222**	MCW0037	N/A
**MCW0103**	MCW0037	N/A
**MCW0016**	MCW0037	N/A
**LEI0166**	MCW0037	N/A
**MCW0248**	MCW0295	N/A
**MCW0111**	MCW0295	N/A
**ADL0268**	MCW0295	N/A
**LEI0234**	MCW0295	N/A
**MCW0206**	MCW0295	N/A
**MCW0034**	MCW0295	N/A
**MCW0222**	MCW0295	N/A
**MCW0103**	MCW0295	N/A
**MCW0016**	MCW0295	N/A
**LEI0166**	MCW0295	N/A
**MCW0037**	MCW0295	N/A
**MCW0248**	LEI0094	1.000
**MCW0111**	LEI0094	1.000
**ADL0268**	LEI0094	1.000
**LEI0234**	LEI0094	N/A
**MCW0206**	LEI0094	N/A
**MCW0034**	LEI0094	N/A
**MCW0222**	LEI0094	1.000
**MCW0103**	LEI0094	1.000
**MCW0016**	LEI0094	N/A
**LEI0166**	LEI0094	1.000
**MCW0037**	LEI0094	N/A
**MCW0295**	LEI0094	N/A
**MCW0248**	MCW0098	0.335
**MCW0111**	MCW0098	0.333
**ADL0268**	MCW0098	1.000
**LEI0234**	MCW0098	N/A
**MCW0206**	MCW0098	N/A
**MCW0034**	MCW0098	N/A

**Locus 1**	**Locus 2**	***p*-value**
**MCW0222**	MCW0098	0.333
**MCW0103**	MCW0098	1.000
**MCW0016**	MCW0098	N/A
**LEI0166**	MCW0098	0.334
**MCW0037**	MCW0098	N/A
**MCW0295**	MCW0098	N/A
**LEI0094**	MCW0098	1.000
**MCW0248**	MCW0078	1.000
**MCW0111**	MCW0078	1.000
**ADL0268**	MCW0078	1.000
**LEI0234**	MCW0078	N/A
**MCW0206**	MCW0078	N/A
**MCW0034**	MCW0078	N/A
**MCW0222**	MCW0078	1.000
**MCW0103**	MCW0078	1.000
**MCW0016**	MCW0078	N/A
**LEI0166**	MCW0078	1.000
**MCW0037**	MCW0078	N/A
**MCW0295**	MCW0078	N/A
**LEI0094**	MCW0078	0.333
**MCW0098**	MCW0078	1.000
**MCW0248**	MCW0081	N/A
**MCW0111**	MCW0081	N/A
**ADL0268**	MCW0081	N/A
**LEI0234**	MCW0081	N/A
**MCW0206**	MCW0081	N/A
**MCW0034**	MCW0081	N/A
**MCW0222**	MCW0081	N/A
**MCW0103**	MCW0081	N/A
**MCW0016**	MCW0081	N/A
**LEI0166**	MCW0081	N/A
**MCW0037**	MCW0081	N/A
**MCW0295**	MCW0081	N/A
**LEI0094**	MCW0081	N/A
**MCW0098**	MCW0081	N/A
**MCW0078**	MCW0081	N/A
**MCW0248**	LEI0192	N/A
**MCW0111**	LEI0192	N/A
**ADL0268**	LEI0192	N/A
**LEI0234**	LEI0192	N/A
**MCW0206**	LEI0192	N/A
**MCW0034**	LEI0192	N/A
**MCW0222**	LEI0192	N/A

**Locus 1**	**Locus 2**	***p*-value**
**MCW0103**	LEI0192	N/A
**MCW0016**	LEI0192	N/A
**LEI0166**	LEI0192	N/A
**MCW0037**	LEI0192	N/A
**MCW0295**	LEI0192	N/A
**LEI0094**	LEI0192	N/A
**MCW0098**	LEI0192	N/A
**MCW0078**	LEI0192	N/A
**MCW0081**	LEI0192	N/A
**MCW0248**	MCW0014	1.000
**MCW0111**	MCW0014	1.000
**ADL0268**	MCW0014	0.337
**LEI0234**	MCW0014	N/A
**MCW0206**	MCW0014	N/A
**MCW0034**	MCW0014	N/A
**MCW0222**	MCW0014	1.000
**MCW0103**	MCW0014	0.332
**MCW0016**	MCW0014	N/A
**LEI0166**	MCW0014	1.000
**MCW0037**	MCW0014	N/A
**MCW0295**	MCW0014	N/A
**LEI0094**	MCW0014	1.000
**MCW0098**	MCW0014	1.000
**MCW0078**	MCW0014	1.000
**MCW0081**	MCW0014	N/A
**LEI0192**	MCW0014	N/A
**MCW0248**	MCW0183	1.000
**MCW0111**	MCW0183	1.000
**ADL0268**	MCW0183	1.000
**LEI0234**	MCW0183	N/A
**MCW0206**	MCW0183	N/A
**MCW0034**	MCW0183	N/A
**MCW0222**	MCW0183	1.000
**MCW0103**	MCW0183	1.000
**MCW0016**	MCW0183	N/A
**LEI0166**	MCW0183	1.000
**MCW0037**	MCW0183	N/A
**MCW0295**	MCW0183	N/A
**LEI0094**	MCW0183	0.331
**MCW0098**	MCW0183	1.000
**MCW0078**	MCW0183	0.332
**MCW0081**	MCW0183	N/A
**LEI0192**	MCW0183	N/A

Locus 1	Locus 2	*p*-value
**MCW0014**	MCW0183	1.000
**MCW0248**	ADL0278	1.000
**MCW0111**	ADL0278	1.000
**ADL0268**	ADL0278	0.332
**LEI0234**	ADL0278	N/A
**MCW0206**	ADL0278	N/A
**MCW0034**	ADL0278	N/A
**MCW0222**	ADL0278	1.000
**MCW0103**	ADL0278	0.330
**MCW0016**	ADL0278	N/A
**LEI0166**	ADL0278	1.000
**MCW0037**	ADL0278	N/A
**MCW0295**	ADL0278	N/A
**LEI0094**	ADL0278	1.000
**MCW0098**	ADL0278	1.000
**MCW0078**	ADL0278	1.000
**MCW0081**	ADL0278	N/A
**LEI0192**	ADL0278	N/A
**MCW0014**	ADL0278	0.332
**MCW0183**	ADL0278	1.000
**MCW0248**	MCW0067	N/A
**MCW0111**	MCW0067	N/A
**ADL0268**	MCW0067	N/A
**LEI0234**	MCW0067	N/A
**MCW0206**	MCW0067	N/A
**MCW0034**	MCW0067	N/A
**MCW0222**	MCW0067	N/A
**MCW0103**	MCW0067	N/A
**MCW0016**	MCW0067	N/A
**LEI0166**	MCW0067	N/A
**MCW0037**	MCW0067	N/A
**MCW0295**	MCW0067	N/A
**LEI0094**	MCW0067	No contingency table
**MCW0098**	MCW0067	N/A
**MCW0078**	MCW0067	No contingency table
**MCW0081**	MCW0067	N/A
**LEI0192**	MCW0067	N/A
**MCW0014**	MCW0067	N/A
**MCW0183**	MCW0067	No contingency table

Locus 1	Locus 2	*p*-value
**ADL0278**	MCW0067	N/A
**MCW0248**	ADL0112	1.000
**MCW0111**	ADL0112	1.000
**ADL0268**	ADL0112	0.335
**LEI0234**	ADL0112	N/A
**MCW0206**	ADL0112	N/A
**MCW0034**	ADL0112	N/A
**MCW0222**	ADL0112	1.000
**MCW0103**	ADL0112	0.332
**MCW0016**	ADL0112	N/A
**LEI0166**	ADL0112	1.000
**MCW0037**	ADL0112	N/A
**MCW0295**	ADL0112	N/A
**LEI0094**	ADL0112	1.000
**MCW0098**	ADL0112	1.000
**MCW0078**	ADL0112	1.000
**MCW0081**	ADL0112	N/A
**LEI0192**	ADL0112	N/A
**MCW0014**	ADL0112	0.337
**MCW0183**	ADL0112	1.000
**ADL0278**	ADL0112	0.330
**MCW0067**	ADL0112	N/A
**MCW0248**	MCW0216	0.331
**MCW0111**	MCW0216	0.335
**ADL0268**	MCW0216	1.000
**LEI0234**	MCW0216	N/A
**MCW0206**	MCW0216	N/A
**MCW0034**	MCW0216	N/A
**MCW0222**	MCW0216	0.335
**MCW0103**	MCW0216	1.000
**MCW0016**	MCW0216	N/A
**LEI0166**	MCW0216	0.335
**MCW0037**	MCW0216	N/A
**MCW0295**	MCW0216	N/A
**LEI0094**	MCW0216	1.000
**MCW0098**	MCW0216	0.333
**MCW0078**	MCW0216	1.000
**MCW0081**	MCW0216	N/A
**LEI0192**	MCW0216	N/A
**MCW0014**	MCW0216	1.000
**MCW0183**	MCW0216	1.000
**ADL0278**	MCW0216	1.000
**MCW0067**	MCW0216	N/A

Locus 1	Locus 2	*p*-value
**ADL0112**	MCW0216	1.000
**MCW0248**	MCW0104	N/A
**MCW0111**	MCW0104	N/A
**ADL0268**	MCW0104	N/A
**LEI0234**	MCW0104	N/A
**MCW0206**	MCW0104	N/A
**MCW0034**	MCW0104	N/A
**MCW0222**	MCW0104	N/A
**MCW0103**	MCW0104	N/A
**MCW0016**	MCW0104	N/A
**LEI0166**	MCW0104	N/A
**MCW0037**	MCW0104	N/A
**MCW0295**	MCW0104	N/A
**LEI0094**	MCW0104	N/A
**MCW0098**	MCW0104	N/A
**MCW0078**	MCW0104	N/A
**MCW0081**	MCW0104	N/A
**LEI0192**	MCW0104	N/A
**MCW0014**	MCW0104	N/A
**MCW0183**	MCW0104	N/A
**ADL0278**	MCW0104	N/A
**MCW0067**	MCW0104	N/A
**ADL0112**	MCW0104	N/A
**MCW0216**	MCW0104	N/A
**MCW0248**	MCW0123	N/A
**MCW0111**	MCW0123	N/A
**ADL0268**	MCW0123	N/A
**LEI0234**	MCW0123	N/A
**MCW0206**	MCW0123	N/A
**MCW0034**	MCW0123	N/A
**MCW0222**	MCW0123	N/A
**MCW0103**	MCW0123	N/A
**MCW0016**	MCW0123	N/A
**LEI0166**	MCW0123	N/A
**MCW0037**	MCW0123	N/A
**MCW0295**	MCW0123	N/A
**LEI0094**	MCW0123	N/A
**MCW0098**	MCW0123	N/A
**MCW0078**	MCW0123	N/A
**MCW0081**	MCW0123	N/A
**LEI0192**	MCW0123	N/A
**MCW0014**	MCW0123	N/A
**MCW0183**	MCW0123	N/A

Locus 1	Locus 2	*p*-value
**ADL0278**	MCW0123	N/A
**MCW0067**	MCW0123	N/A
**ADL0112**	MCW0123	N/A
**MCW0216**	MCW0123	N/A
**MCW0104**	MCW0123	N/A
**MCW0248**	MCW0330	1.000
**MCW0111**	MCW0330	1.000
**ADL0268**	MCW0330	0.334
**LEI0234**	MCW0330	N/A
**MCW0206**	MCW0330	N/A
**MCW0034**	MCW0330	N/A
**MCW0222**	MCW0330	1.000
**MCW0103**	MCW0330	0.332
**MCW0016**	MCW0330	N/A
**LEI0166**	MCW0330	1.000
**MCW0037**	MCW0330	N/A
**MCW0295**	MCW0330	N/A
**LEI0094**	MCW0330	1.000
**MCW0098**	MCW0330	1.000
**MCW0078**	MCW0330	1.000
**MCW0081**	MCW0330	N/A
**LEI0192**	MCW0330	N/A
**MCW0014**	MCW0330	0.336
**MCW0183**	MCW0330	1.000
**ADL0278**	MCW0330	0.334
**MCW0067**	MCW0330	N/A
**ADL0112**	MCW0330	0.334
**MCW0216**	MCW0330	1.000
**MCW0104**	MCW0330	N/A
**MCW0123**	MCW0330	N/A
**MCW0248**	MCW0165	1.000
**MCW0111**	MCW0165	1.000
**ADL0268**	MCW0165	0.336
**LEI0234**	MCW0165	N/A
**MCW0206**	MCW0165	N/A
**MCW0034**	MCW0165	N/A
**MCW0222**	MCW0165	1.000
**MCW0103**	MCW0165	0.332
**MCW0016**	MCW0165	N/A
**LEI0166**	MCW0165	1.000
**MCW0037**	MCW0165	N/A
**MCW0295**	MCW0165	N/A
**LEI0094**	MCW0165	1.000

**Locus 1**	**Locus 2**	***p*-value**
**MCW0098**	MCW0165	1.000
**MCW0078**	MCW0165	1.000
**MCW0081**	MCW0165	N/A
**LEI0192**	MCW0165	N/A
**MCW0014**	MCW0165	0.334
**MCW0183**	MCW0165	1.000
**ADL0278**	MCW0165	0.332
**MCW0067**	MCW0165	N/A
**ADL0112**	MCW0165	0.335
**MCW0216**	MCW0165	1.000
**MCW0104**	MCW0165	N/A
**MCW0123**	MCW0165	N/A
**MCW0330**	MCW0165	0.331
**MCW0248**	MCW0069	0.330
**MCW0111**	MCW0069	0.337
**ADL0268**	MCW0069	1.000
**LEI0234**	MCW0069	N/A
**MCW0206**	MCW0069	N/A
**MCW0034**	MCW0069	N/A
**MCW0222**	MCW0069	0.335
**MCW0103**	MCW0069	1.000
**MCW0016**	MCW0069	N/A
**LEI0166**	MCW0069	0.332
**MCW0037**	MCW0069	N/A
**MCW0295**	MCW0069	N/A
**LEI0094**	MCW0069	1.000
**MCW0098**	MCW0069	0.333
**MCW0078**	MCW0069	1.000
**MCW0081**	MCW0069	N/A
**LEI0192**	MCW0069	N/A
**MCW0014**	MCW0069	1.000
**MCW0183**	MCW0069	1.000
**ADL0278**	MCW0069	1.000
**MCW0067**	MCW0069	N/A
**ADL0112**	MCW0069	1.000
**MCW0216**	MCW0069	0.334
**MCW0104**	MCW0069	N/A
**MCW0123**	MCW0069	N/A
**MCW0330**	MCW0069	1.000
**MCW0165**	MCW0069	1.000
