# Supplementary material for: Samae Dam chicken: a variety of the Pradu Hang Dam breed revealed from microsatellite genotyping data
Source: Anim Biosci. 2024 Jun 25;37(12):2033–43. doi: 10.5713/ab.24.0161 (PMC11541018; doi:10.5713/ab.24.0161)
Supplement: Supplementary file 17 [file ab-24-0161-Supplementary-Table-S9.pdf]

Table S9. Pairwise differentiation of linkage disequilibrium of Samae Dam chickens derived from the Department of Livestock, Uthai Thani (SD1) based on 28 microsatellite loci

Locus 1	Locus 2	*p*-value
**MCW0248**	MCW0111	0.537
**MCW0248**	ADL0268	0.001
**MCW0111**	ADL0268	0.327
**MCW0248**	LEI0234	0.084
**MCW0111**	LEI0234	0.925
**ADL0268**	LEI0234	0.770
**MCW0248**	MCW0206	0.000
**MCW0111**	MCW0206	0.846
**ADL0268**	MCW0206	0.004
**LEI0234**	MCW0206	0.076
**MCW0248**	MCW0034	0.111
**MCW0111**	MCW0034	0.521
**ADL0268**	MCW0034	0.634
**LEI0234**	MCW0034	0.815
**MCW0206**	MCW0034	0.493
**MCW0248**	MCW0222	0.005
**MCW0111**	MCW0222	0.171
**ADL0268**	MCW0222	0.280
**LEI0234**	MCW0222	0.049
**MCW0206**	MCW0222	0.000
**MCW0034**	MCW0222	0.061
**MCW0248**	MCW0103	0.091
**MCW0111**	MCW0103	1.000
**ADL0268**	MCW0103	1.000
**LEI0234**	MCW0103	1.000
**MCW0206**	MCW0103	0.864
**MCW0034**	MCW0103	0.119
**MCW0222**	MCW0103	0.588
**MCW0248**	MCW0016	0.001
**MCW0111**	MCW0016	0.182
**ADL0268**	MCW0016	0.250
**LEI0234**	MCW0016	0.052
**MCW0206**	MCW0016	0.000
**MCW0034**	MCW0016	0.012
**MCW0222**	MCW0016	0.003
**MCW0103**	MCW0016	1.000
**MCW0248**	LEI0166	0.000
**MCW0111**	LEI0166	1.000
**ADL0268**	LEI0166	0.075
**LEI0234**	LEI0166	0.001
**MCW0206**	LEI0166	0.004

Locus 1	Locus 2	*p*-value
**MCW0034**	LEI0166	0.048
**MCW0222**	LEI0166	0.646
**MCW0103**	LEI0166	0.190
**MCW0016**	LEI0166	0.011
**MCW0248**	MCW0037	0.004
**MCW0111**	MCW0037	1.000
**ADL0268**	MCW0037	0.049
**LEI0234**	MCW0037	0.033
**MCW0206**	MCW0037	0.006
**MCW0034**	MCW0037	0.012
**MCW0222**	MCW0037	1.000
**MCW0103**	MCW0037	0.657
**MCW0016**	MCW0037	0.036
**LEI0166**	MCW0037	0.362
**MCW0248**	MCW0295	0.000
**MCW0111**	MCW0295	0.655
**ADL0268**	MCW0295	0.033
**LEI0234**	MCW0295	0.309
**MCW0206**	MCW0295	0.002
**MCW0034**	MCW0295	0.060
**MCW0222**	MCW0295	0.423
**MCW0103**	MCW0295	0.193
**MCW0016**	MCW0295	0.002
**LEI0166**	MCW0295	0.176
**MCW0037**	MCW0295	0.000
**MCW0248**	LEI0094	0.000
**MCW0111**	LEI0094	0.747
**ADL0268**	LEI0094	0.002
**LEI0234**	LEI0094	0.000
**MCW0206**	LEI0094	0.000
**MCW0034**	LEI0094	0.006
**MCW0222**	LEI0094	0.001
**MCW0103**	LEI0094	0.614
**MCW0016**	LEI0094	0.000
**LEI0166**	LEI0094	0.000
**MCW0037**	LEI0094	0.000
**MCW0295**	LEI0094	0.000
**MCW0248**	MCW0098	0.005
**MCW0111**	MCW0098	0.250
**ADL0268**	MCW0098	0.106
**LEI0234**	MCW0098	0.234
**MCW0206**	MCW0098	0.000
**MCW0034**	MCW0098	0.060

Locus 1	Locus 2	*p*-value
**MCW0222**	MCW0098	0.000
**MCW0103**	MCW0098	0.649
**MCW0016**	MCW0098	0.001
**LEI0166**	MCW0098	0.362
**MCW0037**	MCW0098	0.627
**MCW0295**	MCW0098	0.350
**LEI0094**	MCW0098	0.001
**MCW0248**	MCW0078	1.000
**MCW0111**	MCW0078	1.000
**ADL0268**	MCW0078	1.000
**LEI0234**	MCW0078	0.497
**MCW0206**	MCW0078	1.000
**MCW0034**	MCW0078	0.651
**MCW0222**	MCW0078	1.000
**MCW0103**	MCW0078	1.000
**MCW0016**	MCW0078	0.500
**LEI0166**	MCW0078	1.000
**MCW0037**	MCW0078	1.000
**MCW0295**	MCW0078	1.000
**LEI0094**	MCW0078	1.000
**MCW0098**	MCW0078	1.000
**MCW0248**	MCW0081	0.002
**MCW0111**	MCW0081	0.209
**ADL0268**	MCW0081	0.212
**LEI0234**	MCW0081	0.022
**MCW0206**	MCW0081	0.000
**MCW0034**	MCW0081	0.017
**MCW0222**	MCW0081	0.003
**MCW0103**	MCW0081	1.000
**MCW0016**	MCW0081	0.000
**LEI0166**	MCW0081	0.067
**MCW0037**	MCW0081	0.167
**MCW0295**	MCW0081	0.006
**LEI0094**	MCW0081	0.001
**MCW0098**	MCW0081	0.001
**MCW0078**	MCW0081	1.000
**MCW0248**	LEI0192	0.021
**MCW0111**	LEI0192	0.475
**ADL0268**	LEI0192	0.004
**LEI0234**	LEI0192	0.064
**MCW0206**	LEI0192	0.000
**MCW0034**	LEI0192	0.249
**MCW0222**	LEI0192	0.001

**Locus 1**	**Locus 2**	***p*-value**
**MCW0103**	LEI0192	1.000
**MCW0016**	LEI0192	0.006
**LEI0166**	LEI0192	0.117
**MCW0037**	LEI0192	0.113
**MCW0295**	LEI0192	0.026
**LEI0094**	LEI0192	0.000
**MCW0098**	LEI0192	0.003
**MCW0078**	LEI0192	1.000
**MCW0081**	LEI0192	0.001
**MCW0248**	MCW0014	0.001
**MCW0111**	MCW0014	0.675
**ADL0268**	MCW0014	0.141
**LEI0234**	MCW0014	0.131
**MCW0206**	MCW0014	0.000
**MCW0034**	MCW0014	0.073
**MCW0222**	MCW0014	0.002
**MCW0103**	MCW0014	0.763
**MCW0016**	MCW0014	0.000
**LEI0166**	MCW0014	0.001
**MCW0037**	MCW0014	0.221
**MCW0295**	MCW0014	0.025
**LEI0094**	MCW0014	0.000
**MCW0098**	MCW0014	0.001
**MCW0078**	MCW0014	1.000
**MCW0081**	MCW0014	0.000
**LEI0192**	MCW0014	0.034
**MCW0248**	MCW0183	0.005
**MCW0111**	MCW0183	0.169
**ADL0268**	MCW0183	0.281
**LEI0234**	MCW0183	0.055
**MCW0206**	MCW0183	0.000
**MCW0034**	MCW0183	0.055
**MCW0222**	MCW0183	0.000
**MCW0103**	MCW0183	0.592
**MCW0016**	MCW0183	0.003
**LEI0166**	MCW0183	0.644
**MCW0037**	MCW0183	1.000
**MCW0295**	MCW0183	0.420
**LEI0094**	MCW0183	0.001
**MCW0098**	MCW0183	0.000
**MCW0078**	MCW0183	1.000
**MCW0081**	MCW0183	0.003
**LEI0192**	MCW0183	0.002

Locus 1	Locus 2	*p*-value
**MCW0014**	MCW0183	0.003
**MCW0248**	ADL0278	0.304
**MCW0111**	ADL0278	0.334
**ADL0268**	ADL0278	0.106
**LEI0234**	ADL0278	0.816
**MCW0206**	ADL0278	0.356
**MCW0034**	ADL0278	0.931
**MCW0222**	ADL0278	1.000
**MCW0103**	ADL0278	1.000
**MCW0016**	ADL0278	1.000
**LEI0166**	ADL0278	1.000
**MCW0037**	ADL0278	0.351
**MCW0295**	ADL0278	0.405
**LEI0094**	ADL0278	0.261
**MCW0098**	ADL0278	0.121
**MCW0078**	ADL0278	1.000
**MCW0081**	ADL0278	1.000
**LEI0192**	ADL0278	0.654
**MCW0014**	ADL0278	0.396
**MCW0183**	ADL0278	1.000
**MCW0248**	MCW0067	0.000
**MCW0111**	MCW0067	0.063
**ADL0268**	MCW0067	0.061
**LEI0234**	MCW0067	0.034
**MCW0206**	MCW0067	0.005
**MCW0034**	MCW0067	0.027
**MCW0222**	MCW0067	0.373
**MCW0103**	MCW0067	0.171
**MCW0016**	MCW0067	0.001
**LEI0166**	MCW0067	0.000
**MCW0037**	MCW0067	0.265
**MCW0295**	MCW0067	0.024
**LEI0094**	MCW0067	0.000
**MCW0098**	MCW0067	0.280
**MCW0078**	MCW0067	1.000
**MCW0081**	MCW0067	0.001
**LEI0192**	MCW0067	0.073
**MCW0014**	MCW0067	0.000
**MCW0183**	MCW0067	0.370
**ADL0278**	MCW0067	0.937
**MCW0248**	ADL0112	0.268
**MCW0111**	ADL0112	0.012
**ADL0268**	ADL0112	0.242

**Locus 1**	**Locus 2**	***p*-value**
**LEI0234**	ADL0112	0.698
**MCW0206**	ADL0112	0.443
**MCW0034**	ADL0112	0.554
**MCW0222**	ADL0112	0.108
**MCW0103**	ADL0112	1.000
**MCW0016**	ADL0112	0.580
**LEI0166**	ADL0112	0.578
**MCW0037**	ADL0112	0.635
**MCW0295**	ADL0112	0.844
**LEI0094**	ADL0112	0.135
**MCW0098**	ADL0112	0.238
**MCW0078**	ADL0112	0.687
**MCW0081**	ADL0112	0.583
**LEI0192**	ADL0112	0.344
**MCW0014**	ADL0112	0.877
**MCW0183**	ADL0112	0.109
**ADL0278**	ADL0112	0.831
**MCW0067**	ADL0112	0.782
**MCW0248**	MCW0216	0.238
**MCW0111**	MCW0216	0.333
**ADL0268**	MCW0216	0.168
**LEI0234**	MCW0216	0.034
**MCW0206**	MCW0216	0.220
**MCW0034**	MCW0216	0.454
**MCW0222**	MCW0216	0.147
**MCW0103**	MCW0216	1.000
**MCW0016**	MCW0216	0.020
**LEI0166**	MCW0216	0.127
**MCW0037**	MCW0216	0.021
**MCW0295**	MCW0216	0.208
**LEI0094**	MCW0216	0.045
**MCW0098**	MCW0216	0.308
**MCW0078**	MCW0216	0.198
**MCW0081**	MCW0216	0.072
**LEI0192**	MCW0216	0.064
**MCW0014**	MCW0216	0.877
**MCW0183**	MCW0216	0.150
**ADL0278**	MCW0216	0.881
**MCW0067**	MCW0216	0.431
**ADL0112**	MCW0216	0.454
**MCW0248**	MCW0104	0.420
**MCW0111**	MCW0104	0.178
**ADL0268**	MCW0104	0.206

Locus 1	Locus 2	*p*-value
**LEI0234**	MCW0104	0.680
**MCW0206**	MCW0104	0.162
**MCW0034**	MCW0104	1.000
**MCW0222**	MCW0104	0.790
**MCW0103**	MCW0104	1.000
**MCW0016**	MCW0104	0.727
**LEI0166**	MCW0104	0.326
**MCW0037**	MCW0104	0.772
**MCW0295**	MCW0104	0.599
**LEI0094**	MCW0104	0.292
**MCW0098**	MCW0104	0.674
**MCW0078**	MCW0104	1.000
**MCW0081**	MCW0104	0.795
**LEI0192**	MCW0104	0.016
**MCW0014**	MCW0104	0.292
**MCW0183**	MCW0104	0.805
**ADL0278**	MCW0104	0.102
**MCW0067**	MCW0104	0.438
**ADL0112**	MCW0104	0.492
**MCW0216**	MCW0104	0.718
**MCW0248**	MCW0123	N/A
**MCW0111**	MCW0123	N/A
**ADL0268**	MCW0123	N/A
**LEI0234**	MCW0123	N/A
**MCW0206**	MCW0123	N/A
**MCW0034**	MCW0123	N/A
**MCW0222**	MCW0123	N/A
**MCW0103**	MCW0123	N/A
**MCW0016**	MCW0123	N/A
**LEI0166**	MCW0123	N/A
**MCW0037**	MCW0123	N/A
**MCW0295**	MCW0123	N/A
**LEI0094**	MCW0123	N/A
**MCW0098**	MCW0123	N/A
**MCW0078**	MCW0123	N/A
**MCW0081**	MCW0123	N/A
**LEI0192**	MCW0123	N/A
**MCW0014**	MCW0123	N/A
**MCW0183**	MCW0123	N/A
**ADL0278**	MCW0123	N/A
**MCW0067**	MCW0123	N/A
**ADL0112**	MCW0123	N/A
**MCW0216**	MCW0123	N/A

Locus 1	Locus 2	*p*-value
**MCW0104**	MCW0123	N/A
**MCW0248**	MCW0330	0.035
**MCW0111**	MCW0330	0.331
**ADL0268**	MCW0330	0.394
**LEI0234**	MCW0330	0.794
**MCW0206**	MCW0330	0.169
**MCW0034**	MCW0330	0.324
**MCW0222**	MCW0330	0.698
**MCW0103**	MCW0330	0.142
**MCW0016**	MCW0330	0.116
**LEI0166**	MCW0330	0.227
**MCW0037**	MCW0330	0.022
**MCW0295**	MCW0330	0.002
**LEI0094**	MCW0330	0.112
**MCW0098**	MCW0330	0.201
**MCW0078**	MCW0330	1.000
**MCW0081**	MCW0330	0.117
**LEI0192**	MCW0330	0.521
**MCW0014**	MCW0330	0.110
**MCW0183**	MCW0330	0.697
**ADL0278**	MCW0330	0.111
**MCW0067**	MCW0330	0.058
**ADL0112**	MCW0330	0.955
**MCW0216**	MCW0330	0.998
**MCW0104**	MCW0330	0.520
**MCW0123**	MCW0330	N/A
**MCW0248**	MCW0165	0.470
**MCW0111**	MCW0165	1.000
**ADL0268**	MCW0165	0.290
**LEI0234**	MCW0165	0.780
**MCW0206**	MCW0165	0.091
**MCW0034**	MCW0165	1.000
**MCW0222**	MCW0165	0.590
**MCW0103**	MCW0165	1.000
**MCW0016**	MCW0165	1.000
**LEI0166**	MCW0165	0.708
**MCW0037**	MCW0165	0.146
**MCW0295**	MCW0165	0.283
**LEI0094**	MCW0165	0.075
**MCW0098**	MCW0165	0.035
**MCW0078**	MCW0165	1.000
**MCW0081**	MCW0165	0.477
**LEI0192**	MCW0165	0.086

Locus 1	Locus 2	*p*-value
**MCW0014**	MCW0165	0.241
**MCW0183**	MCW0165	0.588
**ADL0278**	MCW0165	0.133
**MCW0067**	MCW0165	0.812
**ADL0112**	MCW0165	0.680
**MCW0216**	MCW0165	0.888
**MCW0104**	MCW0165	0.301
**MCW0123**	MCW0165	N/A
**MCW0330**	MCW0165	0.061
**MCW0248**	MCW0069	0.000
**MCW0111**	MCW0069	1.000
**ADL0268**	MCW0069	0.237
**LEI0234**	MCW0069	0.013
**MCW0206**	MCW0069	0.000
**MCW0034**	MCW0069	0.088
**MCW0222**	MCW0069	0.001
**MCW0103**	MCW0069	0.408
**MCW0016**	MCW0069	0.095
**LEI0166**	MCW0069	0.005
**MCW0037**	MCW0069	0.140
**MCW0295**	MCW0069	0.009
**LEI0094**	MCW0069	0.000
**MCW0098**	MCW0069	0.001
**MCW0078**	MCW0069	1.000
**MCW0081**	MCW0069	0.143
**LEI0192**	MCW0069	0.015
**MCW0014**	MCW0069	0.024
**MCW0183**	MCW0069	0.001
**ADL0278**	MCW0069	0.534
**MCW0067**	MCW0069	0.012
**ADL0112**	MCW0069	0.211
**MCW0216**	MCW0069	0.712
**MCW0104**	MCW0069	0.496
**MCW0123**	MCW0069	N/A
**MCW0330**	MCW0069	0.165
**MCW0165**	MCW0069	0.314
