# Supplementary material for: Samae Dam chicken: a variety of the Pradu Hang Dam breed revealed from microsatellite genotyping data
Source: Anim Biosci. 2024 Jun 25;37(12):2033–43. doi: 10.5713/ab.24.0161 (PMC11541018; doi:10.5713/ab.24.0161)
Supplement: Supplementary file 19 [file ab-24-0161-Supplementary-Table-S11.pdf]

Table S11. Comparison of the observed and expected heterozygosity of Pradu Hang Dam and Samae Dam chickens based on 28 microsatellite loci

Breeds	Locality*	H_o	H_e	*t*-test	*p*-value
Samea Dam	SD1	0.929±0.037	0.627±0.025	6.763	9.854×10^{-8}
	SD2	0.408±0.064	0.379±0.047	0.365	0.728
Pradu Hang Dam	PDH1	0.509±0.040	0.566±0.025	–1.208	0.245
	PDH2	0.429±0.074	0.546±0.039	–1.398	0.255
	PDH3	0.756±0.042	0.696±0.024	–13.519	5.903×10^{-14}
	PDH4	0.643±0.040	0.617±0.030	0.607	0.607
	PDH5	0.619±0.063	0.547±0.030	1.031	0.381

*SD1, Samae Dam (Department of livestock Uthai Thani); SD2, Samae Dam (Sanhawat Farm Uthai Thani); PDH1, Pradu Hang Dam (Phitsanulok 1); PDH2, Pradu Hang Dam (Phitsanulok 2); PDH3, Pradu Hang Dam (Chiang Mai); PDH4, Pradu Hang Dam (Nakhon Pathom); PDH5, Pradu Hang Dam (Nonthaburi)

H_o, Observed heterozygosity; H_e, Expected heterozygosity
