# Supplementary material for: Samae Dam chicken: a variety of the Pradu Hang Dam breed revealed from microsatellite genotyping data
Source: Anim Biosci. 2024 Jun 25;37(12):2033–43. doi: 10.5713/ab.24.0161 (PMC11541018; doi:10.5713/ab.24.0161)
Supplement: Supplementary file 20 [file ab-24-0161-Supplementary-Table-S12.pdf]

Table S12. Inbreeding coefficients (F_{IS}) of Pradu Hang Dam chickens (n = 10) derived from Phitsanulok 1 (PDH1).

Individual	F_{IS}
PDH1	0.393
PDH2	0.449
PDH3	0.181
PDH4	0.628
PDH5	0.062
PDH6	0.478
PDH7	0.286
PDH8	0.367
PDH9	0.133
PDH10	0.337
