# Supplementary material for: Samae Dam chicken: a variety of the Pradu Hang Dam breed revealed from microsatellite genotyping data
Source: Anim Biosci. 2024 Jun 25;37(12):2033–43. doi: 10.5713/ab.24.0161 (PMC11541018; doi:10.5713/ab.24.0161)
Supplement: Supplementary file 21 [file ab-24-0161-Supplementary-Table-S13.pdf]

Table S13. Inbreeding coefficients (F_{IS}) of Pradu Hang Dam chickens (n = 3) derived from Phitsanulok 2 (PDH2).

Individual	F_{IS}
PDH2-1	0.908
PDH2-2	0.888
PDH2-3	1.249
