# Supplementary material for: Samae Dam chicken: a variety of the Pradu Hang Dam breed revealed from microsatellite genotyping data
Source: Anim Biosci. 2024 Jun 25;37(12):2033–43. doi: 10.5713/ab.24.0161 (PMC11541018; doi:10.5713/ab.24.0161)
Supplement: Supplementary file 22 [file ab-24-0161-Supplementary-Table-S14.pdf]

Table S14. Inbreeding coefficients (F_{IS}) of Pradu Hang Dam chicken (n = 19) derived from Chiang Mai (PDH3).

Individual	F_{IS}
PD1	0.074
PD2	–0.026
PD3	0.097
PD4	0.138
PD5	0.189
PD6	0.090
PD7	0.156
PD8	0.468
PD9	0.159
PD10	0.022
PD11	0.042
PD12	0.005
PD13	0.035
PD14	0.072
PD15	0.264
PD16	0.033
PD17	0.209
PD18	–0.055
PD19	0.163
