# Supplementary material for: Samae Dam chicken: a variety of the Pradu Hang Dam breed revealed from microsatellite genotyping data
Source: Anim Biosci. 2024 Jun 25;37(12):2033–43. doi: 10.5713/ab.24.0161 (PMC11541018; doi:10.5713/ab.24.0161)
Supplement: Supplementary file 23 [file ab-24-0161-Supplementary-Table-S15.pdf]

Table S15. Inbreeding coefficients (F_{IS}) of Pradu Hang Dam chickens (n = 16) derived from Nakhon Pathom (PDH4).

Individual	F_{IS}
PDD1	0.450
PDD2	0.060
PDD3	0.209
PDD4	0.064
PDD5	0.198
PDD6	0.062
PDD7	0.309
PDD8	0.014
PDD9	0.224
PDD10	0.116
PDD11	0.066
PDD12	0.041
PDD13	0.065
PDD14	0.052
PDD15	0.138
PDD16	0.253
