# Supplementary material for: Samae Dam chicken: a variety of the Pradu Hang Dam breed revealed from microsatellite genotyping data
Source: Anim Biosci. 2024 Jun 25;37(12):2033–43. doi: 10.5713/ab.24.0161 (PMC11541018; doi:10.5713/ab.24.0161)
Supplement: Supplementary file 24 [file ab-24-0161-Supplementary-Table-S16.pdf]

Table S16. Inbreeding coefficients (F_{IS}) of Pradu Hang Dam chickens (n = 3) derived from Nonthaburi (PDH5)

Individual	F_{IS}
PDn1	0.107
PDn2	0.179
PDN3	0.372
