# Supplementary material for: Samae Dam chicken: a variety of the Pradu Hang Dam breed revealed from microsatellite genotyping data
Source: Anim Biosci. 2024 Jun 25;37(12):2033–43. doi: 10.5713/ab.24.0161 (PMC11541018; doi:10.5713/ab.24.0161)
Supplement: Supplementary file 25 [file ab-24-0161-Supplementary-Table-S17.pdf]

Table S17. Inbreeding coefficients (F_{IS}) of Samae Dam chickens (n = 20) derived from Department of Livestock, Uthai Thani (SD1)

Individual	F_{IS}
SDM1	–0.049
SDM2	–0.055
SDM3	–0.049
SDM4	–0.048
SDM5	–0.056
SDM6	–0.049
SDM7	–0.056
SDM8	0.032
SDM9	–0.066
SDM10	–0.018
SDM11	–0.032
SDM12	0.004
SDM13	–0.048
SDM14	–0.032
SDM15	–0.060
SDM16	–0.067
SDM17	–0.055
SDM18	–0.043
SDM19	–0.043
SDM20	–0.059
