# Supplementary material for: Samae Dam chicken: a variety of the Pradu Hang Dam breed revealed from microsatellite genotyping data
Source: Anim Biosci. 2024 Jun 25;37(12):2033–43. doi: 10.5713/ab.24.0161 (PMC11541018; doi:10.5713/ab.24.0161)
Supplement: Supplementary file 26 [file ab-24-0161-Supplementary-Table-S18.pdf]

Table S18. Inbreeding coefficients (F_{IS}) of Samae Dam chickens (n = 4) derived from Sanhawat Farm, Uthai Thani (SD2).

Individual	F_{IS}
SD1	0.776
SD2	0.421
SD3	0.370
SD4	0.304
