# Supplementary material for: Samae Dam chicken: a variety of the Pradu Hang Dam breed revealed from microsatellite genotyping data
Source: Anim Biosci. 2024 Jun 25;37(12):2033–43. doi: 10.5713/ab.24.0161 (PMC11541018; doi:10.5713/ab.24.0161)
Supplement: Supplementary file 27 [file ab-24-0161-Supplementary-Table-S19.pdf]

Table S19. Pairwise genetic relatedness (*r*) amongst Pradu Hang Dam chicken populations derived from Phitsanulok 1 (PDH1), Phitsanulok 2 (PDH2), Chiang Mai (PDH3), Nakhon Pathom (PDH4) and Nonthaburi (PDH5).

Sample 1	Sample 2	*r*
PDH1	PDH2	0.232
PDH1	PDH3	0.218
PDH2	PDH3	0.186
PDH1	PDH4	0.100
PDH2	PDH4	0.241
PDH3	PDH4	0.142
PDH1	PDH5	0.250
PDH2	PDH5	0.177
PDH3	PDH5	0.172
PDH4	PDH5	0.302
PDH1	PDH6	0.048
PDH2	PDH6	0.138
PDH3	PDH6	0.146
PDH4	PDH6	0.233
PDH5	PDH6	0.122
PDH1	PDH7	0.219
PDH2	PDH7	0.208
PDH3	PDH7	0.187
PDH4	PDH7	0.197
PDH5	PDH7	0.197
PDH6	PDH7	0.249
PDH1	PDH8	0.092
PDH2	PDH8	0.266
PDH3	PDH8	0.161
PDH4	PDH8	0.206
PDH5	PDH8	0.219
PDH6	PDH8	0.304
PDH7	PDH8	0.137
PDH1	PDH9	0.095
PDH2	PDH9	0.167
PDH3	PDH9	0.140
PDH4	PDH9	0.163
PDH5	PDH9	0.211
PDH6	PDH9	0.343
PDH7	PDH9	0.253
PDH8	PDH9	0.326
PDH1	PDH10	0.052
PDH2	PDH10	0.174
PDH3	PDH10	0.128
PDH4	PDH10	0.082

Sample 1	Sample 2	*r*
PDH5	PDH10	0.105
PDH6	PDH10	0.196
PDH7	PDH10	0.204
PDH8	PDH10	0.116
PDH9	PDH10	0.149
PDH1	PDN1	–0.062
PDH2	PDN1	–0.065
PDH3	PDN1	–0.069
PDH4	PDN1	–0.082
PDH5	PDN1	–0.072
PDH6	PDN1	–0.078
PDH7	PDN1	–0.071
PDH8	PDN1	–0.063
PDH9	PDN1	–0.059
PDH10	PDN1	–0.038
PDH1	PDN2	–0.072
PDH2	PDN2	–0.071
PDH3	PDN2	–0.054
PDH4	PDN2	–0.061
PDH5	PDN2	–0.070
PDH6	PDN2	–0.056
PDH7	PDN2	–0.062
PDH8	PDN2	–0.072
PDH9	PDN2	–0.067
PDH10	PDN2	–0.063
PDN1	PDN2	0.332
PDH1	PDN3	–0.043
PDH2	PDN3	–0.049
PDH3	PDN3	–0.076
PDH4	PDN3	–0.078
PDH5	PDN3	–0.076
PDH6	PDN3	–0.069
PDH7	PDN3	–0.054
PDH8	PDN3	–0.068
PDH9	PDN3	–0.068
PDH10	PDN3	–0.031
PDN1	PDN3	0.157
PDN2	PDN3	0.259
PDH1	PD1	–0.047
PDH2	PD1	–0.069

Sample 1	Sample 2	r
PDH3	PD1	–0.050
PDH4	PD1	–0.071
PDH5	PD1	–0.063
PDH6	PD1	–0.066
PDH7	PD1	–0.077
PDH8	PD1	–0.061
PDH9	PD1	–0.068
PDH10	PD1	–0.059
PDN1	PD1	–0.022
PDN2	PD1	–0.030
PDN3	PD1	–0.047
PDH1	PD2	–0.065
PDH2	PD2	–0.069
PDH3	PD2	–0.070
PDH4	PD2	–0.075
PDH5	PD2	–0.035
PDH6	PD2	–0.072
PDH7	PD2	–0.076
PDH8	PD2	–0.063
PDH9	PD2	–0.068
PDH10	PD2	–0.066
PDN1	PD2	–0.050
PDN2	PD2	–0.038
PDN3	PD2	–0.050
PD1	PD2	0.079
PDH1	PD3	–0.058
PDH2	PD3	–0.048
PDH3	PD3	–0.051
PDH4	PD3	–0.055
PDH5	PD3	–0.036
PDH6	PD3	–0.058
PDH7	PD3	–0.052
PDH8	PD3	–0.034
PDH9	PD3	–0.051
PDH10	PD3	–0.029
PDN1	PD3	–0.048
PDN2	PD3	–0.061
PDN3	PD3	–0.048
PD1	PD3	0.055
PD2	PD3	0.049
PDH1	PD4	–0.075
PDH2	PD4	–0.043
PDH3	PD4	–0.071

Sample 1	Sample 2	r
PDH4	PD4	–0.078
PDH5	PD4	–0.053
PDH6	PD4	–0.061
PDH7	PD4	–0.029
PDH8	PD4	–0.047
PDH9	PD4	–0.046
PDH10	PD4	–0.037
PDN1	PD4	–0.045
PDN2	PD4	–0.032
PDN3	PD4	–0.044
PD1	PD4	0.061
PD2	PD4	0.093
PD3	PD4	0.022
PDH1	PD5	–0.080
PDH2	PD5	–0.077
PDH3	PD5	–0.080
PDH4	PD5	–0.076
PDH5	PD5	–0.072
PDH6	PD5	–0.077
PDH7	PD5	–0.078
PDH8	PD5	–0.075
PDH9	PD5	–0.074
PDH10	PD5	–0.080
PDN1	PD5	–0.066
PDN2	PD5	–0.050
PDN3	PD5	–0.065
PD1	PD5	0.031
PD2	PD5	0.075
PD3	PD5	0.047
PD4	PD5	0.121
PDH1	PD6	–0.055
PDH2	PD6	–0.079
PDH3	PD6	–0.072
PDH4	PD6	–0.063
PDH5	PD6	–0.064
PDH6	PD6	–0.078
PDH7	PD6	–0.080
PDH8	PD6	–0.081
PDH9	PD6	–0.069
PDH10	PD6	–0.048
PDN1	PD6	–0.069
PDN2	PD6	–0.052
PDN3	PD6	–0.056

Sample 1	Sample 2	*r*
PD1	PD6	0.091
PD2	PD6	0.121
PD3	PD6	0.072
PD4	PD6	0.156
PD5	PD6	0.130
PDH1	PD7	–0.073
PDH2	PD7	–0.079
PDH3	PD7	–0.070
PDH4	PD7	–0.075
PDH5	PD7	–0.072
PDH6	PD7	–0.077
PDH7	PD7	–0.076
PDH8	PD7	–0.076
PDH9	PD7	–0.076
PDH10	PD7	–0.072
PDN1	PD7	–0.013
PDN2	PD7	–0.049
PDN3	PD7	–0.014
PD1	PD7	0.091
PD2	PD7	0.039
PD3	PD7	0.055
PD4	PD7	0.035
PD5	PD7	0.062
PD6	PD7	0.080
PDH1	PD8	–0.036
PDH2	PD8	–0.044
PDH3	PD8	–0.058
PDH4	PD8	–0.043
PDH5	PD8	–0.061
PDH6	PD8	–0.071
PDH7	PD8	–0.068
PDH8	PD8	–0.042
PDH9	PD8	–0.056
PDH10	PD8	–0.051
PDN1	PD8	–0.036
PDN2	PD8	–0.058
PDN3	PD8	–0.041
PD1	PD8	0.124
PD2	PD8	0.026
PD3	PD8	0.106
PD4	PD8	0.002
PD5	PD8	0.022
PD6	PD8	0.034
PD7	PD8	0.063
PDH1	PD9	–0.060
PDH2	PD9	–0.066
PDH3	PD9	–0.074
PDH4	PD9	–0.066
PDH5	PD9	–0.068
PDH6	PD9	–0.071
PDH7	PD9	–0.080
PDH8	PD9	–0.065
PDH9	PD9	–0.067
PDH10	PD9	–0.070
PDN1	PD9	–0.060
PDN2	PD9	–0.044
PDN3	PD9	–0.030
PD1	PD9	0.093
PD2	PD9	0.132
PD3	PD9	0.013
PD4	PD9	0.090
PD5	PD9	0.119
PD6	PD9	0.058
PD7	PD9	0.069
PD8	PD9	0.013
PDH1	PD10	–0.077
PDH2	PD10	–0.077
PDH3	PD10	–0.073
PDH4	PD10	–0.079
PDH5	PD10	–0.066
PDH6	PD10	–0.077
PDH7	PD10	–0.076
PDH8	PD10	–0.072
PDH9	PD10	–0.067
PDH10	PD10	–0.065
PDN1	PD10	0.015
PDN2	PD10	–0.049
PDN3	PD10	–0.012
PD1	PD10	0.047
PD2	PD10	0.053
PD3	PD10	0.097
PD4	PD10	0.096
PD5	PD10	0.013
PD6	PD10	0.022
PD7	PD10	0.075
PD8	PD10	0.045

Sample 1	Sample 2	*r*
PD9	PD10	0.059
PDH1	PD11	–0.070
PDH2	PD11	–0.078
PDH3	PD11	–0.074
PDH4	PD11	–0.081
PDH5	PD11	–0.066
PDH6	PD11	–0.078
PDH7	PD11	–0.078
PDH8	PD11	–0.072
PDH9	PD11	–0.068
PDH10	PD11	–0.065
PDN1	PD11	–0.044
PDN2	PD11	–0.029
PDN3	PD11	–0.050
PD1	PD11	0.040
PD2	PD11	0.056
PD3	PD11	0.134
PD4	PD11	0.008
PD5	PD11	0.047
PD6	PD11	0.051
PD7	PD11	0.099
PD8	PD11	0.079
PD9	PD11	0.047
PD10	PD11	0.139
PDH1	PD12	–0.059
PDH2	PD12	–0.071
PDH3	PD12	–0.078
PDH4	PD12	–0.080
PDH5	PD12	–0.077
PDH6	PD12	–0.073
PDH7	PD12	–0.085
PDH8	PD12	–0.068
PDH9	PD12	–0.075
PDH10	PD12	–0.075
PDN1	PD12	–0.039
PDN2	PD12	–0.032
PDN3	PD12	–0.014
PD1	PD12	0.033
PD2	PD12	0.111
PD3	PD12	0.002
PD4	PD12	0.009
PD5	PD12	0.032
PD6	PD12	0.048

Sample 1	Sample 2	*r*
PD7	PD12	0.084
PD8	PD12	0.070
PD9	PD12	0.065
PD10	PD12	0.088
PD11	PD12	0.127
PDH1	PD13	–0.046
PDH2	PD13	–0.072
PDH3	PD13	–0.072
PDH4	PD13	–0.076
PDH5	PD13	–0.074
PDH6	PD13	–0.075
PDH7	PD13	–0.085
PDH8	PD13	–0.066
PDH9	PD13	–0.067
PDH10	PD13	–0.070
PDN1	PD13	–0.044
PDN2	PD13	–0.048
PDN3	PD13	–0.019
PD1	PD13	0.082
PD2	PD13	0.040
PD3	PD13	0.075
PD4	PD13	0.029
PD5	PD13	0.136
PD6	PD13	0.039
PD7	PD13	0.183
PD8	PD13	0.025
PD9	PD13	0.082
PD10	PD13	0.071
PD11	PD13	0.113
PD12	PD13	0.093
PDH1	PD14	–0.047
PDH2	PD14	–0.077
PDH3	PD14	–0.064
PDH4	PD14	–0.064
PDH5	PD14	–0.064
PDH6	PD14	–0.076
PDH7	PD14	–0.075
PDH8	PD14	–0.071
PDH9	PD14	–0.071
PDH10	PD14	–0.071
PDN1	PD14	–0.049
PDN2	PD14	–0.015
PDN3	PD14	–0.031

Sample 1	Sample 2	*r*
PD1	PD14	0.048
PD2	PD14	0.075
PD3	PD14	–0.009
PD4	PD14	0.068
PD5	PD14	0.073
PD6	PD14	0.023
PD7	PD14	0.063
PD8	PD14	0.011
PD9	PD14	0.088
PD10	PD14	0.044
PD11	PD14	0.098
PD12	PD14	0.092
PD13	PD14	0.117
PDH1	PD15	–0.062
PDH2	PD15	–0.076
PDH3	PD15	–0.059
PDH4	PD15	–0.074
PDH5	PD15	–0.064
PDH6	PD15	–0.076
PDH7	PD15	–0.059
PDH8	PD15	–0.071
PDH9	PD15	–0.062
PDH10	PD15	–0.058
PDN1	PD15	0.020
PDN2	PD15	–0.055
PDN3	PD15	–0.042
PD1	PD15	0.041
PD2	PD15	0.068
PD3	PD15	0.021
PD4	PD15	0.033
PD5	PD15	0.069
PD6	PD15	0.048
PD7	PD15	0.084
PD8	PD15	0.048
PD9	PD15	0.052
PD10	PD15	0.039
PD11	PD15	0.033
PD12	PD15	0.041
PD13	PD15	0.080
PD14	PD15	0.130
PDH1	PD16	–0.034
PDH2	PD16	–0.061
PDH3	PD16	–0.036

Sample 1	Sample 2	*r*
PDH4	PD16	–0.053
PDH5	PD16	–0.042
PDH6	PD16	–0.059
PDH7	PD16	–0.049
PDH8	PD16	–0.045
PDH9	PD16	–0.039
PDH10	PD16	–0.042
PDN1	PD16	–0.041
PDN2	PD16	–0.014
PDN3	PD16	–0.044
PD1	PD16	0.060
PD2	PD16	0.074
PD3	PD16	0.028
PD4	PD16	0.037
PD5	PD16	0.094
PD6	PD16	0.081
PD7	PD16	0.073
PD8	PD16	0.034
PD9	PD16	0.068
PD10	PD16	0.041
PD11	PD16	0.053
PD12	PD16	0.063
PD13	PD16	0.064
PD14	PD16	0.058
PD15	PD16	0.063
PDH1	PD17	–0.053
PDH2	PD17	–0.075
PDH3	PD17	–0.056
PDH4	PD17	–0.008
PDH5	PD17	–0.051
PDH6	PD17	–0.067
PDH7	PD17	–0.056
PDH8	PD17	–0.066
PDH9	PD17	–0.059
PDH10	PD17	–0.059
PDN1	PD17	–0.034
PDN2	PD17	0.040
PDN3	PD17	0.015
PD1	PD17	0.014
PD2	PD17	0.073
PD3	PD17	0.136
PD4	PD17	0.026
PD5	PD17	0.026

Sample 1	Sample 2	*r*
PD6	PD17	0.012
PD7	PD17	0.031
PD8	PD17	0.110
PD9	PD17	0.028
PD10	PD17	0.038
PD11	PD17	0.096
PD12	PD17	0.023
PD13	PD17	0.013
PD14	PD17	0.073
PD15	PD17	0.062
PD16	PD17	0.042
PDH1	PD18	–0.037
PDH2	PD18	–0.067
PDH3	PD18	–0.056
PDH4	PD18	–0.046
PDH5	PD18	–0.057
PDH6	PD18	–0.069
PDH7	PD18	–0.084
PDH8	PD18	–0.063
PDH9	PD18	–0.058
PDH10	PD18	–0.058
PDN1	PD18	–0.025
PDN2	PD18	–0.004
PDN3	PD18	–0.025
PD1	PD18	0.065
PD2	PD18	0.080
PD3	PD18	0.054
PD4	PD18	0.041
PD5	PD18	0.055
PD6	PD18	0.137
PD7	PD18	0.031
PD8	PD18	0.081
PD9	PD18	0.032
PD10	PD18	0.035
PD11	PD18	0.087
PD12	PD18	0.049
PD13	PD18	0.064
PD14	PD18	0.061
PD15	PD18	0.042
PD16	PD18	0.071
PD17	PD18	0.086
PDH1	PD19	–0.049
PDH2	PD19	–0.069

Sample 1	Sample 2	*r*
PDH3	PD19	–0.056
PDH4	PD19	–0.052
PDH5	PD19	–0.043
PDH6	PD19	–0.053
PDH7	PD19	–0.080
PDH8	PD19	–0.047
PDH9	PD19	–0.060
PDH10	PD19	–0.075
PDN1	PD19	–0.055
PDN2	PD19	–0.053
PDN3	PD19	–0.027
PD1	PD19	0.016
PD2	PD19	0.025
PD3	PD19	0.098
PD4	PD19	0.014
PD5	PD19	0.042
PD6	PD19	0.045
PD7	PD19	0.030
PD8	PD19	0.083
PD9	PD19	0.073
PD10	PD19	0.105
PD11	PD19	0.056
PD12	PD19	0.043
PD13	PD19	0.035
PD14	PD19	0.023
PD15	PD19	0.213
PD16	PD19	0.045
PD17	PD19	0.073
PD18	PD19	0.060
PDH1	PDN1	–0.048
PDH2	PDN1	–0.072
PDH3	PDN1	–0.063
PDH4	PDN1	–0.043
PDH5	PDN1	–0.049
PDH6	PDN1	–0.058
PDH7	PDN1	–0.077
PDH8	PDN1	–0.062
PDH9	PDN1	–0.038
PDH10	PDN1	–0.020
PDN1	PDN1	–0.022
PDN2	PDN1	–0.049
PDN3	PDN1	0.007
PD1	PDN1	–0.050

Sample 1	Sample 2	*r*
PD2	PDN1	–0.066
PD3	PDN1	–0.044
PD4	PDN1	–0.066
PD5	PDN1	–0.044
PD6	PDN1	–0.049
PD7	PDN1	–0.041
PD8	PDN1	–0.049
PD9	PDN1	–0.054
PD10	PDN1	–0.030
PD11	PDN1	–0.049
PD12	PDN1	–0.015
PD13	PDN1	–0.059
PD14	PDN1	–0.057
PD15	PDN1	–0.027
PD16	PDN1	–0.054
PD17	PDN1	–0.066
PD18	PDN1	–0.071
PD19	PDN1	0.013
PDH1	PDN2	–0.043
PDH2	PDN2	–0.051
PDH3	PDN2	–0.028
PDH4	PDN2	–0.052
PDH5	PDN2	–0.045
PDH6	PDN2	–0.041
PDH7	PDN2	–0.053
PDH8	PDN2	–0.039
PDH9	PDN2	–0.042
PDH10	PDN2	–0.032
PDN1	PDN2	–0.055
PDN2	PDN2	–0.065
PDN3	PDN2	–0.055
PD1	PDN2	–0.042
PD2	PDN2	–0.037
PD3	PDN2	–0.055
PD4	PDN2	–0.047
PD5	PDN2	–0.050
PD6	PDN2	–0.039
PD7	PDN2	–0.059
PD8	PDN2	–0.058
PD9	PDN2	–0.056
PD10	PDN2	–0.056
PD11	PDN2	–0.056
PD12	PDN2	–0.042

Sample 1	Sample 2	*r*
PD13	PDN2	–0.051
PD14	PDN2	–0.046
PD15	PDN2	–0.044
PD16	PDN2	–0.054
PD17	PDN2	–0.057
PD18	PDN2	–0.044
PD19	PDN2	–0.063
PDN1	PDN2	0.053
PDH1	PDN3	–0.071
PDH2	PDN3	–0.051
PDH3	PDN3	–0.040
PDH4	PDN3	–0.046
PDH5	PDN3	–0.045
PDH6	PDN3	–0.047
PDH7	PDN3	0.006
PDH8	PDN3	–0.061
PDH9	PDN3	–0.047
PDH10	PDN3	–0.036
PDN1	PDN3	–0.045
PDN2	PDN3	–0.064
PDN3	PDN3	–0.027
PD1	PDN3	–0.051
PD2	PDN3	–0.059
PD3	PDN3	–0.051
PD4	PDN3	–0.054
PD5	PDN3	–0.062
PD6	PDN3	–0.054
PD7	PDN3	–0.070
PD8	PDN3	–0.061
PD9	PDN3	–0.069
PD10	PDN3	–0.060
PD11	PDN3	–0.058
PD12	PDN3	–0.047
PD13	PDN3	–0.059
PD14	PDN3	–0.054
PD15	PDN3	–0.062
PD16	PDN3	–0.073
PD17	PDN3	–0.043
PD18	PDN3	–0.063
PD19	PDN3	–0.073
PDN1	PDN3	–0.014
PDN2	PDN3	0.064
PDH1	PDN4	–0.057

Sample 1	Sample 2	*r*
PDH2	PDN4	–0.053
PDH3	PDN4	–0.033
PDH4	PDN4	–0.054
PDH5	PDN4	–0.052
PDH6	PDN4	–0.044
PDH7	PDN4	–0.057
PDH8	PDN4	–0.044
PDH9	PDN4	–0.045
PDH10	PDN4	–0.052
PDN1	PDN4	–0.064
PDN2	PDN4	–0.020
PDN3	PDN4	–0.037
PD1	PDN4	–0.059
PD2	PDN4	–0.053
PD3	PDN4	–0.064
PD4	PDN4	–0.064
PD5	PDN4	–0.041
PD6	PDN4	–0.055
PD7	PDN4	–0.057
PD8	PDN4	–0.046
PD9	PDN4	–0.056
PD10	PDN4	–0.045
PD11	PDN4	–0.063
PD12	PDN4	–0.053
PD13	PDN4	–0.053
PD14	PDN4	–0.050
PD15	PDN4	–0.053
PD16	PDN4	–0.042
PD17	PDN4	–0.050
PD18	PDN4	–0.050
PD19	PDN4	–0.057
PDN1	PDN4	0.039
PDN2	PDN4	0.117
PDN3	PDN4	0.079
PDH1	PDN5	–0.056
PDH2	PDN5	–0.049
PDH3	PDN5	–0.053
PDH4	PDN5	–0.065
PDH5	PDN5	–0.044
PDH6	PDN5	–0.054
PDH7	PDN5	–0.047
PDH8	PDN5	–0.053
PDH9	PDN5	–0.043

Sample 1	Sample 2	*r*
PDH10	PDN5	–0.039
PDN1	PDN5	–0.041
PDN2	PDN5	–0.059
PDN3	PDN5	–0.058
PD1	PDN5	–0.055
PD2	PDN5	–0.052
PD3	PDN5	–0.059
PD4	PDN5	–0.044
PD5	PDN5	–0.044
PD6	PDN5	–0.046
PD7	PDN5	–0.062
PD8	PDN5	–0.053
PD9	PDN5	–0.051
PD10	PDN5	–0.035
PD11	PDN5	–0.063
PD12	PDN5	–0.051
PD13	PDN5	–0.060
PD14	PDN5	–0.070
PD15	PDN5	–0.067
PD16	PDN5	–0.047
PD17	PDN5	–0.053
PD18	PDN5	–0.046
PD19	PDN5	–0.056
PDN1	PDN5	0.052
PDN2	PDN5	0.104
PDN3	PDN5	0.099
PDN4	PDN5	0.055
PDH1	PDN6	–0.056
PDH2	PDN6	–0.065
PDH3	PDN6	–0.039
PDH4	PDN6	–0.066
PDH5	PDN6	–0.055
PDH6	PDN6	–0.069
PDH7	PDN6	–0.073
PDH8	PDN6	–0.067
PDH9	PDN6	–0.054
PDH10	PDN6	–0.036
PDN1	PDN6	–0.033
PDN2	PDN6	–0.054
PDN3	PDN6	–0.064
PD1	PDN6	–0.063
PD2	PDN6	–0.039
PD3	PDN6	–0.064

Sample 1	Sample 2	*r*
PD4	PDN6	–0.037
PD5	PDN6	–0.047
PD6	PDN6	–0.021
PD7	PDN6	–0.067
PD8	PDN6	–0.059
PD9	PDN6	–0.055
PD10	PDN6	–0.043
PD11	PDN6	–0.064
PD12	PDN6	–0.049
PD13	PDN6	–0.064
PD14	PDN6	–0.063
PD15	PDN6	–0.055
PD16	PDN6	–0.056
PD17	PDN6	–0.050
PD18	PDN6	–0.043
PD19	PDN6	–0.063
PDN1	PDN6	0.024
PDN2	PDN6	0.069
PDN3	PDN6	0.066
PDN4	PDN6	0.090
PDN5	PDN6	0.071
PDH1	PDN7	–0.050
PDH2	PDN7	–0.043
PDH3	PDN7	–0.038
PDH4	PDN7	–0.074
PDH5	PDN7	–0.055
PDH6	PDN7	–0.057
PDH7	PDN7	–0.079
PDH8	PDN7	–0.056
PDH9	PDN7	–0.049
PDH10	PDN7	0.003
PDN1	PDN7	–0.054
PDN2	PDN7	–0.064
PDN3	PDN7	–0.006
PD1	PDN7	–0.068
PD2	PDN7	–0.057
PD3	PDN7	–0.073
PD4	PDN7	–0.055
PD5	PDN7	–0.071
PD6	PDN7	–0.043
PD7	PDN7	–0.054
PD8	PDN7	–0.063
PD9	PDN7	–0.063

Sample 1	Sample 2	*r*
PD10	PDN7	–0.063
PD11	PDN7	–0.071
PD12	PDN7	–0.055
PD13	PDN7	–0.058
PD14	PDN7	–0.060
PD15	PDN7	–0.073
PD16	PDN7	–0.060
PD17	PDN7	–0.073
PD18	PDN7	–0.054
PD19	PDN7	–0.072
PDN1	PDN7	0.030
PDN2	PDN7	0.111
PDN3	PDN7	0.076
PDN4	PDN7	0.096
PDN5	PDN7	0.100
PDN6	PDN7	0.061
PDH1	PDN8	–0.063
PDH2	PDN8	–0.051
PDH3	PDN8	–0.031
PDH4	PDN8	–0.070
PDH5	PDN8	–0.044
PDH6	PDN8	–0.054
PDH7	PDN8	–0.023
PDH8	PDN8	–0.058
PDH9	PDN8	–0.038
PDH10	PDN8	–0.017
PDN1	PDN8	–0.040
PDN2	PDN8	–0.054
PDN3	PDN8	–0.061
PD1	PDN8	–0.062
PD2	PDN8	–0.053
PD3	PDN8	–0.059
PD4	PDN8	–0.059
PD5	PDN8	–0.027
PD6	PDN8	–0.041
PD7	PDN8	–0.055
PD8	PDN8	–0.058
PD9	PDN8	–0.026
PD10	PDN8	–0.042
PD11	PDN8	–0.059
PD12	PDN8	–0.051
PD13	PDN8	–0.067
PD14	PDN8	–0.061

Sample 1	Sample 2	*r*
PD15	PDN8	–0.059
PD16	PDN8	–0.051
PD17	PDN8	–0.052
PD18	PDN8	–0.059
PD19	PDN8	–0.059
PDN1	PDN8	0.037
PDN2	PDN8	0.044
PDN3	PDN8	0.047
PDN4	PDN8	0.094
PDN5	PDN8	0.060
PDN6	PDN8	0.182
PDN7	PDN8	0.064
PDH1	PDN9	–0.063
PDH2	PDN9	–0.035
PDH3	PDN9	–0.039
PDH4	PDN9	–0.066
PDH5	PDN9	–0.042
PDH6	PDN9	–0.046
PDH7	PDN9	–0.068
PDH8	PDN9	–0.045
PDH9	PDN9	–0.052
PDH10	PDN9	–0.028
PDN1	PDN9	–0.045
PDN2	PDN9	–0.060
PDN3	PDN9	–0.030
PD1	PDN9	–0.041
PD2	PDN9	–0.038
PD3	PDN9	–0.055
PD4	PDN9	–0.054
PD5	PDN9	–0.040
PD6	PDN9	–0.044
PD7	PDN9	–0.058
PD8	PDN9	–0.049
PD9	PDN9	–0.054
PD10	PDN9	–0.046
PD11	PDN9	–0.063
PD12	PDN9	–0.047
PD13	PDN9	–0.047
PD14	PDN9	–0.045
PD15	PDN9	–0.048
PD16	PDN9	–0.040
PD17	PDN9	–0.045
PD18	PDN9	–0.048

Sample 1	Sample 2	*r*
PD19	PDN9	–0.053
PDN1	PDN9	–0.011
PDN2	PDN9	0.045
PDN3	PDN9	0.074
PDN4	PDN9	0.095
PDN5	PDN9	0.039
PDN6	PDN9	0.081
PDN7	PDN9	0.086
PDN8	PDN9	0.093
PDH1	PDN10	–0.053
PDH2	PDN10	–0.042
PDH3	PDN10	–0.037
PDH4	PDN10	–0.060
PDH5	PDN10	–0.048
PDH6	PDN10	–0.046
PDH7	PDN10	–0.058
PDH8	PDN10	–0.050
PDH9	PDN10	–0.041
PDH10	PDN10	–0.035
PDN1	PDN10	–0.049
PDN2	PDN10	–0.058
PDN3	PDN10	–0.045
PD1	PDN10	–0.053
PD2	PDN10	–0.050
PD3	PDN10	–0.059
PD4	PDN10	–0.063
PD5	PDN10	–0.049
PD6	PDN10	–0.037
PD7	PDN10	–0.067
PD8	PDN10	–0.062
PD9	PDN10	–0.055
PD10	PDN10	–0.043
PD11	PDN10	–0.061
PD12	PDN10	–0.052
PD13	PDN10	–0.060
PD14	PDN10	–0.056
PD15	PDN10	–0.062
PD16	PDN10	–0.059
PD17	PDN10	–0.056
PD18	PDN10	–0.057
PD19	PDN10	–0.066
PDN1	PDN10	0.027
PDN2	PDN10	0.106

Sample 1	Sample 2	*r*
PDN3	PDN10	0.111
PDN4	PDN10	0.103
PDN5	PDN10	0.064
PDN6	PDN10	0.081
PDN7	PDN10	0.091
PDN8	PDN10	0.109
PDN9	PDN10	0.098
PDH1	PDN11	–0.019
PDH2	PDN11	–0.040
PDH3	PDN11	–0.017
PDH4	PDN11	–0.075
PDH5	PDN11	–0.046
PDH6	PDN11	–0.062
PDH7	PDN11	–0.073
PDH8	PDN11	–0.056
PDH9	PDN11	–0.037
PDH10	PDN11	–0.062
PDN1	PDN11	–0.038
PDN2	PDN11	–0.074
PDN3	PDN11	–0.071
PD1	PDN11	–0.029
PD2	PDN11	–0.063
PD3	PDN11	–0.062
PD4	PDN11	–0.053
PD5	PDN11	–0.066
PD6	PDN11	–0.071
PD7	PDN11	–0.066
PD8	PDN11	–0.052
PD9	PDN11	–0.065
PD10	PDN11	–0.027
PD11	PDN11	–0.060
PD12	PDN11	–0.058
PD13	PDN11	–0.025
PD14	PDN11	–0.062
PD15	PDN11	–0.060
PD16	PDN11	–0.060
PD17	PDN11	–0.064
PD18	PDN11	–0.029
PD19	PDN11	–0.060
PDN1	PDN11	0.048
PDN2	PDN11	0.022
PDN3	PDN11	0.015
PDN4	PDN11	0.048

Sample 1	Sample 2	*r*
PDN5	PDN11	0.044
PDN6	PDN11	0.079
PDN7	PDN11	0.032
PDN8	PDN11	0.054
PDN9	PDN11	0.096
PDN10	PDN11	0.086
PDH1	PDN12	–0.057
PDH2	PDN12	–0.040
PDH3	PDN12	–0.035
PDH4	PDN12	–0.026
PDH5	PDN12	–0.043
PDH6	PDN12	–0.040
PDH7	PDN12	–0.010
PDH8	PDN12	–0.055
PDH9	PDN12	–0.021
PDH10	PDN12	–0.016
PDN1	PDN12	–0.050
PDN2	PDN12	–0.069
PDN3	PDN12	–0.063
PD1	PDN12	–0.062
PD2	PDN12	–0.043
PD3	PDN12	–0.020
PD4	PDN12	–0.052
PD5	PDN12	–0.064
PD6	PDN12	–0.030
PD7	PDN12	–0.066
PD8	PDN12	–0.068
PD9	PDN12	–0.056
PD10	PDN12	–0.030
PD11	PDN12	–0.058
PD12	PDN12	–0.063
PD13	PDN12	–0.064
PD14	PDN12	–0.070
PD15	PDN12	–0.069
PD16	PDN12	–0.059
PD17	PDN12	–0.064
PD18	PDN12	–0.042
PD19	PDN12	–0.074
PDN1	PDN12	0.008
PDN2	PDN12	0.059
PDN3	PDN12	0.073
PDN4	PDN12	0.074
PDN5	PDN12	0.110

Sample 1	Sample 2	*r*
PDN6	PDN12	0.059
PDN7	PDN12	0.099
PDN8	PDN12	0.078
PDN9	PDN12	0.028
PDN10	PDN12	0.078
PDN11	PDN12	0.093
PDH1	PDN13	–0.049
PDH2	PDN13	–0.043
PDH3	PDN13	–0.047
PDH4	PDN13	–0.071
PDH5	PDN13	–0.056
PDH6	PDN13	–0.053
PDH7	PDN13	–0.071
PDH8	PDN13	–0.054
PDH9	PDN13	–0.047
PDH10	PDN13	0.057
PDN1	PDN13	–0.011
PDN2	PDN13	–0.067
PDN3	PDN13	–0.053
PD1	PDN13	–0.037
PD2	PDN13	–0.041
PD3	PDN13	–0.060
PD4	PDN13	–0.042
PD5	PDN13	–0.060
PD6	PDN13	–0.027
PD7	PDN13	–0.059
PD8	PDN13	–0.052
PD9	PDN13	–0.048
PD10	PDN13	–0.021
PD11	PDN13	–0.054
PD12	PDN13	–0.054
PD13	PDN13	–0.048
PD14	PDN13	–0.067
PD15	PDN13	–0.063
PD16	PDN13	–0.043
PD17	PDN13	–0.071
PD18	PDN13	–0.035
PD19	PDN13	–0.068
PDN1	PDN13	0.036
PDN2	PDN13	0.074
PDN3	PDN13	0.030
PDN4	PDN13	0.028
PDN5	PDN13	0.083

Sample 1	Sample 2	*r*
PDN6	PDN13	0.093
PDN7	PDN13	0.060
PDN8	PDN13	0.056
PDN9	PDN13	0.085
PDN10	PDN13	0.031
PDN11	PDN13	0.122
PDN12	PDN13	0.117
PDH1	PDN14	–0.059
PDH2	PDN14	–0.060
PDH3	PDN14	–0.045
PDH4	PDN14	–0.057
PDH5	PDN14	–0.045
PDH6	PDN14	–0.046
PDH7	PDN14	–0.041
PDH8	PDN14	–0.050
PDH9	PDN14	–0.034
PDH10	PDN14	–0.049
PDN1	PDN14	–0.057
PDN2	PDN14	–0.055
PDN3	PDN14	–0.044
PD1	PDN14	–0.062
PD2	PDN14	–0.060
PD3	PDN14	–0.059
PD4	PDN14	–0.055
PD5	PDN14	–0.049
PD6	PDN14	–0.037
PD7	PDN14	–0.066
PD8	PDN14	–0.066
PD9	PDN14	–0.059
PD10	PDN14	–0.049
PD11	PDN14	–0.053
PD12	PDN14	–0.050
PD13	PDN14	–0.059
PD14	PDN14	–0.055
PD15	PDN14	–0.057
PD16	PDN14	–0.059
PD17	PDN14	–0.058
PD18	PDN14	–0.059
PD19	PDN14	–0.066
PDN1	PDN14	0.026
PDN2	PDN14	0.087
PDN3	PDN14	0.149
PDN4	PDN14	0.137

Sample 1	Sample 2	*r*
PDN5	PDN14	0.045
PDN6	PDN14	0.121
PDN7	PDN14	0.097
PDN8	PDN14	0.133
PDN9	PDN14	0.036
PDN10	PDN14	0.140
PDN11	PDN14	–0.001
PDN12	PDN14	0.074
PDN13	PDN14	0.015
PDH1	PDN15	–0.026
PDH2	PDN15	–0.056
PDH3	PDN15	–0.042
PDH4	PDN15	–0.031
PDH5	PDN15	–0.060
PDH6	PDN15	–0.050
PDH7	PDN15	0.017
PDH8	PDN15	–0.040
PDH9	PDN15	–0.038
PDH10	PDN15	–0.014
PDN1	PDN15	–0.056
PDN2	PDN15	–0.065
PDN3	PDN15	–0.039
PD1	PDN15	–0.057
PD2	PDN15	–0.059
PD3	PDN15	–0.056
PD4	PDN15	–0.061
PD5	PDN15	–0.059
PD6	PDN15	–0.053
PD7	PDN15	–0.057
PD8	PDN15	–0.062
PD9	PDN15	–0.061
PD10	PDN15	–0.059
PD11	PDN15	–0.059
PD12	PDN15	–0.056
PD13	PDN15	–0.061
PD14	PDN15	–0.047
PD15	PDN15	–0.064
PD16	PDN15	–0.067
PD17	PDN15	–0.047
PD18	PDN15	–0.056
PD19	PDN15	–0.070
PDN1	PDN15	–0.002
PDN2	PDN15	0.051

Sample 1	Sample 2	*r*
PDN3	PDN15	0.176
PDN4	PDN15	0.076
PDN5	PDN15	0.138
PDN6	PDN15	0.059
PDN7	PDN15	0.092
PDN8	PDN15	0.082
PDN9	PDN15	0.087
PDN10	PDN15	0.088
PDN11	PDN15	0.017
PDN12	PDN15	0.100
PDN13	PDN15	0.054
PDN14	PDN15	0.108
PDH1	PDN16	–0.047
PDH2	PDN16	–0.057
PDH3	PDN16	–0.017
PDH4	PDN16	–0.061
PDH5	PDN16	–0.042
PDH6	PDN16	–0.049
PDH7	PDN16	–0.032
PDH8	PDN16	–0.049
PDH9	PDN16	–0.057
PDH10	PDN16	–0.036
PDN1	PDN16	–0.027
PDN2	PDN16	–0.036
PDN3	PDN16	–0.050
PD1	PDN16	–0.050
PD2	PDN16	–0.048
PD3	PDN16	–0.052
PD4	PDN16	–0.056
PD5	PDN16	–0.064
PD6	PDN16	–0.051
PD7	PDN16	–0.060
PD8	PDN16	–0.064
PD9	PDN16	–0.022
PD10	PDN16	–0.059
PD11	PDN16	–0.016
PD12	PDN16	–0.064
PD13	PDN16	–0.055
PD14	PDN16	–0.067
PD15	PDN16	–0.054
PD16	PDN16	–0.059
PD17	PDN16	–0.058
PD18	PDN16	–0.058

Sample 1	Sample 2	*r*
PD19	PDN16	–0.064
PDN1	PDN16	0.050
PDN2	PDN16	0.074
PDN3	PDN16	0.084
PDN4	PDN16	0.024
PDN5	PDN16	0.121
PDN6	PDN16	0.040
PDN7	PDN16	0.107
PDN8	PDN16	0.040
PDN9	PDN16	0.061
PDN10	PDN16	0.031
PDN11	PDN16	0.045
PDN12	PDN16	0.089
PDN13	PDN16	0.093
PDN14	PDN16	0.040
PDN15	PDN16	0.077
PDH1	PDL1	–0.061
PDH2	PDL1	–0.060
PDH3	PDL1	–0.021
PDH4	PDL1	–0.076
PDH5	PDL1	–0.062
PDH6	PDL1	–0.053
PDH7	PDL1	–0.079
PDH8	PDL1	–0.037
PDH9	PDL1	–0.047
PDH10	PDL1	–0.030
PDN1	PDL1	–0.066
PDN2	PDL1	–0.058
PDN3	PDL1	–0.046
PD1	PDL1	–0.046
PD2	PDL1	–0.047
PD3	PDL1	–0.068
PD4	PDL1	–0.046
PD5	PDL1	–0.027
PD6	PDL1	–0.067
PD7	PDL1	–0.048
PD8	PDL1	–0.012
PD9	PDL1	–0.051
PD10	PDL1	–0.041
PD11	PDL1	–0.041
PD12	PDL1	–0.050
PD13	PDL1	–0.027
PD14	PDL1	–0.026

Sample 1	Sample 2	*r*
PD15	PDL1	–0.049
PD16	PDL1	–0.047
PD17	PDL1	–0.064
PD18	PDL1	–0.066
PD19	PDL1	–0.064
PDN1	PDL1	0.121
PDN2	PDL1	0.094
PDN3	PDL1	0.017
PDN4	PDL1	0.071
PDN5	PDL1	0.031
PDN6	PDL1	0.051
PDN7	PDL1	0.039
PDN8	PDL1	0.038
PDN9	PDL1	0.009
PDN10	PDL1	0.039
PDN11	PDL1	0.089
PDN12	PDL1	0.034
PDN13	PDL1	0.069
PDN14	PDL1	0.037
PDN15	PDL1	0.007
PDN16	PDL1	0.020
PDH1	PDL2	–0.019
PDH2	PDL2	–0.024
PDH3	PDL2	–0.023
PDH4	PDL2	–0.042
PDH5	PDL2	–0.050
PDH6	PDL2	–0.059
PDH7	PDL2	–0.034
PDH8	PDL2	–0.054
PDH9	PDL2	–0.045
PDH10	PDL2	–0.069
PDN1	PDL2	–0.068
PDN2	PDL2	–0.045
PDN3	PDL2	–0.033
PD1	PDL2	–0.058
PD2	PDL2	–0.055
PD3	PDL2	–0.058
PD4	PDL2	–0.053
PD5	PDL2	–0.043
PD6	PDL2	–0.050
PD7	PDL2	–0.066
PD8	PDL2	–0.057
PD9	PDL2	–0.058

Sample 1	Sample 2	*r*
PD10	PDL2	–0.040
PD11	PDL2	–0.058
PD12	PDL2	–0.044
PD13	PDL2	–0.054
PD14	PDL2	–0.064
PD15	PDL2	–0.060
PD16	PDL2	–0.052
PD17	PDL2	–0.055
PD18	PDL2	–0.058
PD19	PDL2	–0.060
PDN1	PDL2	0.088
PDN2	PDL2	0.070
PDN3	PDL2	0.054
PDN4	PDL2	0.089
PDN5	PDL2	0.019
PDN6	PDL2	0.092
PDN7	PDL2	0.017
PDN8	PDL2	0.080
PDN9	PDL2	0.052
PDN10	PDL2	0.066
PDN11	PDL2	0.050
PDN12	PDL2	0.044
PDN13	PDL2	–0.021
PDN14	PDL2	0.052
PDN15	PDL2	0.022
PDN16	PDL2	0.046
PDL1	PDL2	0.098
PDH1	PDL3	–0.054
PDH2	PDL3	–0.059
PDH3	PDL3	–0.036
PDH4	PDL3	–0.039
PDH5	PDL3	–0.021
PDH6	PDL3	–0.047
PDH7	PDL3	–0.056
PDH8	PDL3	–0.047
PDH9	PDL3	–0.025
PDH10	PDL3	–0.046
PDN1	PDL3	–0.069
PDN2	PDL3	–0.071

Sample 1	Sample 2	*r*
PDN3	PDL3	–0.012
PD1	PDL3	–0.046
PD2	PDL3	–0.056
PD3	PDL3	–0.043
PD4	PDL3	–0.045
PD5	PDL3	–0.020
PD6	PDL3	–0.056
PD7	PDL3	–0.067
PD8	PDL3	–0.063
PD9	PDL3	–0.058
PD10	PDL3	–0.050
PD11	PDL3	–0.057
PD12	PDL3	–0.053
PD13	PDL3	–0.053
PD14	PDL3	–0.052
PD15	PDL3	–0.045
PD16	PDL3	–0.059
PD17	PDL3	–0.047
PD18	PDL3	–0.064
PD19	PDL3	–0.055
PDN1	PDL3	0.129
PDN2	PDL3	0.046
PDN3	PDL3	0.032
PDN4	PDL3	0.022
PDN5	PDL3	0.052
PDN6	PDL3	0.027
PDN7	PDL3	0.011
PDN8	PDL3	0.002
PDN9	PDL3	0.032
PDN10	PDL3	0.000
PDN11	PDL3	0.074
PDN12	PDL3	0.034
PDN13	PDL3	0.054
PDN14	PDL3	0.016
PDN15	PDL3	0.028
PDN16	PDL3	0.040
PDL1	PDL3	0.127
PDL2	PDL3	0.089
