# Supplementary material for: Samae Dam chicken: a variety of the Pradu Hang Dam breed revealed from microsatellite genotyping data
Source: Anim Biosci. 2024 Jun 25;37(12):2033–43. doi: 10.5713/ab.24.0161 (PMC11541018; doi:10.5713/ab.24.0161)
Supplement: Supplementary file 28 [file ab-24-0161-Supplementary-Table-S20.pdf]

Table S20. Pairwise genetic relatedness (*r*) among Samae Dam chicken populations derived from the Department of Livestock, Uthai Thani (SD1) and the Sanhawat Farm, Uthai Thani (SD2)

Sample 1	Sample 2	*r*
SDM1	SDM2	0.143
SDM1	SDM3	0.084
SDM2	SDM3	0.075
SDM1	SDM4	0.074
SDM2	SDM4	0.093
SDM3	SDM4	0.059
SDM1	SDM5	0.054
SDM2	SDM5	0.120
SDM3	SDM5	0.052
SDM4	SDM5	0.064
SDM1	SDM6	0.092
SDM2	SDM6	0.123
SDM3	SDM6	0.134
SDM4	SDM6	0.077
SDM5	SDM6	0.127
SDM1	SDM7	0.090
SDM2	SDM7	0.071
SDM3	SDM7	0.095
SDM4	SDM7	0.067
SDM5	SDM7	0.108
SDM6	SDM7	0.098
SDM1	SDM8	0.101
SDM2	SDM8	0.062
SDM3	SDM8	0.072
SDM4	SDM8	0.051
SDM5	SDM8	0.048
SDM6	SDM8	0.079
SDM7	SDM8	0.240
SDM1	SDM9	–0.009
SDM2	SDM9	0.007
SDM3	SDM9	–0.015
SDM4	SDM9	0.010
SDM5	SDM9	0.016
SDM6	SDM9	–0.015
SDM7	SDM9	0.015
SDM8	SDM9	–0.023
SDM1	SDM10	0.015
SDM2	SDM10	0.017
SDM3	SDM10	0.007
SDM4	SDM10	0.026

Sample 1	Sample 2	*r*
SDM5	SDM10	0.003
SDM6	SDM10	–0.012
SDM7	SDM10	–0.009
SDM8	SDM10	0.022
SDM9	SDM10	0.232
SDM1	SDFM1	–0.022
SDM2	SDFM1	–0.041
SDM3	SDFM1	–0.030
SDM4	SDFM1	–0.018
SDM5	SDFM1	–0.045
SDM6	SDFM1	–0.052
SDM7	SDFM1	–0.056
SDM8	SDFM1	–0.015
SDM9	SDFM1	–0.003
SDM10	SDFM1	0.041
SDM1	SDFM2	–0.045
SDM2	SDFM2	–0.054
SDM3	SDFM2	–0.026
SDM4	SDFM2	–0.035
SDM5	SDFM2	–0.035
SDM6	SDFM2	–0.026
SDM7	SDFM2	–0.033
SDM8	SDFM2	–0.046
SDM9	SDFM2	–0.027
SDM10	SDFM2	–0.021
SDFM1	SDFM2	0.124
SDM1	SDFM3	–0.039
SDM2	SDFM3	–0.054
SDM3	SDFM3	–0.041
SDM4	SDFM3	–0.029
SDM5	SDFM3	–0.044
SDM6	SDFM3	–0.043
SDM7	SDFM3	–0.052
SDM8	SDFM3	–0.040
SDM9	SDFM3	–0.017
SDM10	SDFM3	–0.022
SDFM1	SDFM3	0.101
SDFM2	SDFM3	0.157
SDM1	SDFM4	–0.026
SDM2	SDFM4	–0.045

Sample 1	Sample 2	*r*
SDM3	SDFM4	–0.008
SDM4	SDFM4	–0.021
SDM5	SDFM4	–0.042
SDM6	SDFM4	–0.020
SDM7	SDFM4	–0.052
SDM8	SDFM4	–0.040
SDM9	SDFM4	–0.012
SDM10	SDFM4	–0.007
SDFM1	SDFM4	0.116
SDFM2	SDFM4	0.135
SDFM3	SDFM4	0.105
SDM1	SDFM5	–0.021
SDM2	SDFM5	–0.049
SDM3	SDFM5	–0.012
SDM4	SDFM5	–0.011
SDM5	SDFM5	–0.025
SDM6	SDFM5	–0.011
SDM7	SDFM5	–0.046
SDM8	SDFM5	–0.024
SDM9	SDFM5	0.011
SDM10	SDFM5	–0.005
SDFM1	SDFM5	0.003
SDFM2	SDFM5	–0.002
SDFM3	SDFM5	–0.004
SDFM4	SDFM5	0.027
SDM1	SDFM6	–0.055
SDM2	SDFM6	–0.043
SDM3	SDFM6	–0.018
SDM4	SDFM6	–0.041
SDM5	SDFM6	–0.050
SDM6	SDFM6	–0.049
SDM7	SDFM6	–0.062
SDM8	SDFM6	–0.052
SDM9	SDFM6	–0.010
SDM10	SDFM6	–0.027
SDFM1	SDFM6	0.057
SDFM2	SDFM6	0.010
SDFM3	SDFM6	–0.011
SDFM4	SDFM6	0.002
SDFM5	SDFM6	0.175
SDM1	SDFM7	–0.052
SDM2	SDFM7	–0.069
SDM3	SDFM7	–0.039

Sample 1	Sample 2	*r*
SDM4	SDFM7	–0.037
SDM5	SDFM7	–0.049
SDM6	SDFM7	–0.046
SDM7	SDFM7	–0.069
SDM8	SDFM7	–0.055
SDM9	SDFM7	0.003
SDM10	SDFM7	–0.017
SDFM1	SDFM7	0.012
SDFM2	SDFM7	–0.005
SDFM3	SDFM7	0.012
SDFM4	SDFM7	–0.001
SDFM5	SDFM7	0.178
SDFM6	SDFM7	0.167
SDM1	SDFM8	–0.039
SDM2	SDFM8	–0.034
SDM3	SDFM8	0.024
SDM4	SDFM8	–0.029
SDM5	SDFM8	–0.043
SDM6	SDFM8	–0.045
SDM7	SDFM8	–0.032
SDM8	SDFM8	–0.037
SDM9	SDFM8	–0.030
SDM10	SDFM8	–0.043
SDFM1	SDFM8	–0.007
SDFM2	SDFM8	–0.008
SDFM3	SDFM8	0.018
SDFM4	SDFM8	–0.002
SDFM5	SDFM8	0.002
SDFM6	SDFM8	0.009
SDFM7	SDFM8	0.007
SDM1	SDFM9	–0.038
SDM2	SDFM9	–0.055
SDM3	SDFM9	–0.027
SDM4	SDFM9	0.008
SDM5	SDFM9	–0.042
SDM6	SDFM9	–0.049
SDM7	SDFM9	–0.051
SDM8	SDFM9	–0.031
SDM9	SDFM9	–0.042
SDM10	SDFM9	–0.028
SDFM1	SDFM9	0.014
SDFM2	SDFM9	0.020
SDFM3	SDFM9	0.035

Sample 1	Sample 2	*r*
SDFM4	SDFM9	0.020
SDFM5	SDFM9	0.011
SDFM6	SDFM9	–0.013
SDFM7	SDFM9	0.009
SDFM8	SDFM9	0.157
SDM1	SDFM10	–0.041
SDM2	SDFM10	–0.048
SDM3	SDFM10	–0.030
SDM4	SDFM10	–0.030
SDM5	SDFM10	–0.045
SDM6	SDFM10	–0.044
SDM7	SDFM10	–0.047
SDM8	SDFM10	–0.050
SDM9	SDFM10	–0.047
SDM10	SDFM10	–0.040
SDFM1	SDFM10	0.003
SDFM2	SDFM10	0.000
SDFM3	SDFM10	0.007
SDFM4	SDFM10	0.000
SDFM5	SDFM10	–0.015
SDFM6	SDFM10	–0.031
SDFM7	SDFM10	0.069
SDFM8	SDFM10	0.142
SDFM9	SDFM10	0.195
SDM1	SD1	–0.124
SDM2	SD1	–0.124
SDM3	SD1	–0.124
SDM4	SD1	–0.109
SDM5	SD1	–0.120
SDM6	SD1	–0.119
SDM7	SD1	–0.133
SDM8	SD1	–0.131
SDM9	SD1	–0.123
SDM10	SD1	–0.127
SDFM1	SD1	–0.147
SDFM2	SD1	–0.126
SDFM3	SD1	–0.144
SDFM4	SD1	–0.135
SDFM5	SD1	–0.142
SDFM6	SD1	–0.134
SDFM7	SD1	–0.135
SDFM8	SD1	–0.131
SDFM9	SD1	–0.131

Sample 1	Sample 2	*r*
SDFM10	SD1	–0.126
SDM1	SD2	–0.125
SDM2	SD2	–0.116
SDM3	SD2	–0.125
SDM4	SD2	–0.104
SDM5	SD2	–0.125
SDM6	SD2	–0.132
SDM7	SD2	–0.140
SDM8	SD2	–0.125
SDM9	SD2	–0.123
SDM10	SD2	–0.128
SDFM1	SD2	–0.119
SDFM2	SD2	–0.119
SDFM3	SD2	–0.115
SDFM4	SD2	–0.119
SDFM5	SD2	–0.119
SDFM6	SD2	–0.073
SDFM7	SD2	–0.113
SDFM8	SD2	–0.125
SDFM9	SD2	–0.125
SDFM10	SD2	–0.121
SD1	SD2	0.399
SDM1	SD3	–0.145
SDM2	SD3	–0.136
SDM3	SD3	–0.145
SDM4	SD3	–0.128
SDM5	SD3	–0.145
SDM6	SD3	–0.158
SDM7	SD3	–0.153
SDM8	SD3	–0.141
SDM9	SD3	–0.127
SDM10	SD3	–0.124
SDFM1	SD3	–0.132
SDFM2	SD3	–0.139
SDFM3	SD3	–0.135
SDFM4	SD3	–0.139
SDFM5	SD3	–0.127
SDFM6	SD3	–0.097
SDFM7	SD3	–0.124
SDFM8	SD3	–0.122
SDFM9	SD3	–0.094
SDFM10	SD3	–0.125
SD1	SD3	0.349

Sample 1	Sample 2	*r*
SD2	SD3	0.402
SDM1	SD4	–0.129
SDM2	SD4	–0.135
SDM3	SD4	–0.129
SDM4	SD4	–0.121
SDM5	SD4	–0.142
SDM6	SD4	–0.136
SDM7	SD4	–0.157
SDM8	SD4	–0.142
SDM9	SD4	–0.115
SDM10	SD4	–0.120
SDFM1	SD4	–0.115
SDFM2	SD4	–0.115
SDFM3	SD4	–0.110
SDFM4	SD4	–0.090
SDFM5	SD4	–0.110
SDFM6	SD4	–0.110
SDFM7	SD4	–0.108
SDFM8	SD4	–0.133
SDFM9	SD4	–0.133
SDFM10	SD4	–0.129
SD1	SD4	0.483
SD2	SD4	0.448
SD3	SD4	0.541
