# Supplementary material for: Samae Dam chicken: a variety of the Pradu Hang Dam breed revealed from microsatellite genotyping data
Source: Anim Biosci. 2024 Jun 25;37(12):2033–43. doi: 10.5713/ab.24.0161 (PMC11541018; doi:10.5713/ab.24.0161)
Supplement: Supplementary file 29 [file ab-24-0161-Supplementary-Table-S21.pdf]

Table S21. The results of analysis of molecular variance (AMOVA) of Pradu Hang Dam (PDH) and Samae Dam (SD) chickens based on 28 microsatellites loci.

Breeds	Source of variation	d.f.	Sum of squares	Variance component	Percentage of variance
**Samae Dam**	Among populations	1	66.958	4.565	29%
	Among Individuals within populations	22	134.000	0.000	0%
	Within individuals	24	274.000	11.417	71%
	Total	47	401.917	30.442	100%
**Pradu Dang Dam**	Among populations	4	267.116	3.065	24%
	Among Individuals within populations	46	492.767	1.028	8%
	Within individuals	51	441.500	8.657	68%
	Total	101	1201.382	12.749	100%
