# Supplementary material for: Samae Dam chicken: a variety of the Pradu Hang Dam breed revealed from microsatellite genotyping data
Source: Anim Biosci. 2024 Jun 25;37(12):2033–43. doi: 10.5713/ab.24.0161 (PMC11541018; doi:10.5713/ab.24.0161)
Supplement: Supplementary file 31 [file ab-24-0161-Supplementary-Table-S23.pdf]

Table S23. Pairwise population differentiation (F_{ST}) between Pradu Hang Dam chicken derived from Phitsanulok 1 (PDH1), Phitsanulok 2 (PDH2), Chiang Mai (PDH3), Nakhon Pathom (PDH4), Nonthaburi (PDH5), and Samae Dam chicken derived from Department of Livestock Uthai Thani (SD1), and Sanhawat Farm Uthai Thani (SD2) populations based on 28 microsatellite loci.

F_{ST}	SD1	SD2	PDH1	PDH2	PDH3	PDH4	PDH5
SD1	0.000						
SD2	0.360*	0.000					
PDH1	0.307*	0.440*	0.000				
PDH2	0.287*	0.340^ns^	0.351*	0.000			
PDH3	0.211*	0.237*	0.268*	0.183*	0.000		
PDH4	0.301*	0.390*	0.302*	0.306*	0.260*	0.000	
PDH5	0.282*	0.371*	0.292*	0.279*	0.213*	0.043^ns^	0.000

* *p*-value <0.05. ns, not significant.
