# Supplementary material for: Samae Dam chicken: a variety of the Pradu Hang Dam breed revealed from microsatellite genotyping data
Source: Anim Biosci. 2024 Jun 25;37(12):2033–43. doi: 10.5713/ab.24.0161 (PMC11541018; doi:10.5713/ab.24.0161)
Supplement: Supplementary file 32 [file ab-24-0161-Supplementary-Table-S24.pdf]

Table S24. Current migration rates among populations of Pradu Hang Dam chickens derived from Phitsanulok 1 (PDH1), Phitsanulok 2 (PDH2), Chiang Mai (PDH3), Nakhon Pathom (PDH4), and Nonthaburi (PDH5) (populations), and Samae Dam chickens derived from Department of Livestock Uthai Thani (SD1) and Sanhawat Farm Uthai Thani (SD2) (populations) based on 28 microsatellite loci generated by BayesAss

Origin	Destination	Posterior mean of migration rates	SD
**SD1**	SD1	0.920	0.026
**SD2**	SD1	0.012	0.012
**PDH1**	SD1	0.012	0.012
**PDH2**	SD1	0.012	0.012
**PDH3**	SD1	0.012	0.012
**PDH4**	SD1	0.012	0.012
**PDH5**	SD1	0.012	0.012
**SD1**	SD2	0.030	0.028
**SD2**	SD2	0.818	0.048
**PDH1**	SD2	0.030	0.027
**PDH2**	SD2	0.030	0.028
**PDH3**	SD2	0.030	0.028
**PDH4**	SD2	0.030	0.028
**PDH5**	SD2	0.030	0.028
**SD1**	PDH1	0.020	0.018
**SD2**	PDH1	0.019	0.018
**PDH1**	PDH1	0.883	0.038
**PDH2**	PDH1	0.020	0.019
**PDH3**	PDH1	0.020	0.019
**PDH4**	PDH1	0.020	0.019
**PDH5**	PDH1	0.020	0.019
**SD1**	PDH2	0.033	0.030
**SD2**	PDH2	0.134	0.049
**PDH1**	PDH2	0.033	0.030
**PDH2**	PDH2	0.700	0.030
**PDH3**	PDH2	0.033	0.030
**PDH4**	PDH2	0.033	0.030
**PDH5**	PDH2	0.033	0.030
**SD1**	PDH3	0.013	0.012
**SD2**	PDH3	0.013	0.012
**PDH1**	PDH3	0.013	0.012
**PDH2**	PDH3	0.013	0.013
**PDH3**	PDH3	0.923	0.027
**PDH4**	PDH3	0.013	0.012
**PDH5**	PDH3	0.013	0.012
**SD1**	PDH4	0.015	0.014
**SD2**	PDH4	0.015	0.014
**PDH1**	PDH4	0.015	0.014
**PDH2**	PDH4	0.015	0.014
**PDH3**	PDH4	0.014	0.014
**PDH4**	PDH4	0.913	0.030

Origin	Destination	Posterior mean of migration rates	SD
**PDH5**	PDH4	0.014	0.014
**SD1**	PDH5	0.033	0.030
**SD2**	PDH5	0.033	0.030
**PDH1**	PDH5	0.034	0.030
**PDH2**	PDH5	0.033	0.030
**PDH3**	PDH5	0.034	0.030
**PDH4**	PDH5	0.133	0.049
**PDH5**	PDH5	0.700	0.030
