# Supplementary material for: Samae Dam chicken: a variety of the Pradu Hang Dam breed revealed from microsatellite genotyping data
Source: Anim Biosci. 2024 Jun 25;37(12):2033–43. doi: 10.5713/ab.24.0161 (PMC11541018; doi:10.5713/ab.24.0161)
Supplement: Supplementary file 33 [file ab-24-0161-Supplementary-Table-S25.pdf]

Table S25. Bayesian estimates of mutation-scaled effective population sizes (*Θ*) and asymmetric migration rates (*M*) among Pradu Hang Dam chicken derived from Phitsanulok 1 (PDH1), Phitsanulok 2 (PDH2), Chiang Mai (PDH3), Nakhon Pathom (PDH4), Nonthaburi (PDH5), and Samae Dam chicken derived from Department of Livestock Uthai Thani (SD1), and Sanhawat Farm Uthai Thani (SD2) based on 28 microsatellite loci generated in MIGRATE-N

Parameter	2.5%	25%	Mode	75%	97.5%
**Θ_SD1**	0.000	0.000	0.001	0.001	0.003
**Θ_SD2**	0.000	0.000	0.000	0.001	0.002
**Θ_PDH1**	0.096	0.098	0.099	0.099	0.100
**Θ_PDH2**	0.000	0.000	0.001	0.001	0.002
**Θ_PDH3**	0.000	0.000	0.000	0.001	0.002
**Θ_PDH4**	0.000	0.000	0.000	0.001	0.002
**Θ_PDH5**	0.000	0.000	0.001	0.002	0.003
**M_SD2->SD1**	0.000	1.330	7.000	12.670	23.330
**M_PDH1->SD1**	0.000	7.330	15.000	22.000	32.000
**M_PDH2->SD1**	0.000	5.330	12.330	18.670	28.670
**M_PDH3->SD1**	0.000	1.330	7.670	13.330	24.000
**M_PDH4->SD1**	0.000	3.330	10.330	16.670	28.000
**M_PDH5->SD1**	0.000	2.670	9.000	14.670	25.330
**M_SD1->SD2**	5.330	45.330	57.000	68.000	80.000
**M_PDH1->SD2**	2.670	14.000	22.330	30.000	42.670
**M_PDH2->SD2**	0.000	1.330	7.670	13.330	24.670
**M_PDH3->SD2**	0.000	2.670	9.670	15.330	27.330
**M_PDH4->SD2**	0.000	0.000	2.330	8.670	20.000
**M_PDH5->SD2**	0.000	2.670	9.000	14.670	24.670
**M_SD1->PDH1**	0.000	2.670	9.000	14.670	24.670
**M_SD2->PDH1**	2.670	12.670	20.330	28.000	37.330
**M_PDH2->PDH1**	0.000	2.670	9.670	15.330	28.000
**M_PDH3->PDH1**	0.000	8.000	15.670	22.670	32.000
**M_PDH4->PDH1**	0.000	7.330	15.000	22.000	31.330
**M_PDH5->PDH1**	0.000	4.670	11.670	18.000	27.330
**M_SD1->PDH2**	178.000	241.330	255.000	271.330	298.670
**M_SD2->PDH2**	51.330	65.330	75.670	84.670	116.000
**M_PDH1->PDH2**	0.000	0.000	8.330	20.670	30.670
**M_PDH3->PDH2**	740.000	759.330	777.670	796.670	881.330
**M_PDH4->PDH2**	116.000	140.670	150.330	159.330	177.330
**M_PDH5->PDH2**	57.330	82.000	91.670	102.000	132.000
**M_SD1->PDH3**	0.000	8.000	18.330	28.670	58.000
**M_SD2->PDH3**	0.000	4.000	10.330	16.670	26.670
**M_PDH1->PDH3**	0.000	7.330	15.000	22.000	33.330
**M_PDH2->PDH3**	77.330	88.670	97.670	106.670	118.000
**M_PDH4->PDH3**	122.670	142.670	155.670	168.000	226.000
**M_PDH5->PDH3**	34.000	50.000	59.000	66.670	81.330
**M_SD1->PDH4**	6.000	17.330	25.000	33.330	44.670
**M_SD2->PDH4**	0.000	2.000	8.330	14.000	24.000
**M_PDH1->PDH4**	0.000	8.670	16.330	23.330	32.670
**M_PDH2->PDH4**	0.000	3.330	10.330	16.000	27.330
**M_PDH3->PDH4**	0.000	19.330	35.000	44.000	62.670

Parameter	2.5%	25%	Mode	75%	97.5%
**M_PDH5->PDH4**	0.000	7.330	15.000	22.000	33.330
**M_SD1->PDH5**	0.000	8.670	16.330	23.330	32.670
**M_SD2->PDH5**	0.000	7.330	15.000	22.000	30.670
**M_PDH1->PDH5**	0.000	8.670	17.000	24.670	36.670
**M_PDH2->PDH5**	9.330	24.000	35.000	46.670	90.670
**M_PDH3->PDH5**	6.670	13.330	27.000	40.000	47.330
**M_PDH4->PDH5**	255.330	302.670	322.330	339.330	357.330
