# Supplementary material for: Samae Dam chicken: a variety of the Pradu Hang Dam breed revealed from microsatellite genotyping data
Source: Anim Biosci. 2024 Jun 25;37(12):2033–43. doi: 10.5713/ab.24.0161 (PMC11541018; doi:10.5713/ab.24.0161)
Supplement: Supplementary file 34 [file ab-24-0161-Supplementary-Table-S26.pdf]

Table S26. The effective number of immigrants (N_m) from population i into population j per generation generated in MIGRATE-N.

i / j	SD1	SD2	PDH1	PDH2	PDH3	PDH4	PDH5
**SD1**		0.000	0.222	0.040	0.000	0.000	0.004
**SD2**	0.001		0.501	0.012	0.000	0.000	0.004
**PDH1**	0.002	0.000		0.001	0.000	0.000	0.004
**PDH2**	0.002	0.000	0.238		0.002	0.000	0.008
**PDH3**	0.001	0.000	0.386	0.122		0.000	0.007
**PDH4**	0.002	0.000	0.370	0.024	0.004		0.078
**PDH5**	0.001	0.000	0.288	0.014	0.001	0.000	

SD1, Samae Dam (Department of livestock Uthai Thani); SM2, Samae Dam (Sanhawat Farm Uthai Thani); PDH1,= Pradu Hang Dam (Phitsanulok 1); PDH2, Pradu Hang Dam (Phitsanulok 2);PDH3, Pradu Hang Dam (Chiang Mai); PDH4, Pradu Hang Dam (Nakhon Pathom); PDH5,Pradu Hang Dam (Nonthaburi)
